# ﻿*Pleurophragmium parvisporum* (*Ascomycota*): One name, seven stories – a case highlighting the need for verification of strains from public culture collections

**DOI:** 10.3897/imafungus.16.173033

**Published:** 2025-11-25

**Authors:** Martina Réblová, Jana Nekvindová, Lucie Bauchová, Margarita Hernández-Restrepo

**Affiliations:** 1 Czech Academy of Sciences, Institute of Botany, Department of Taxonomy, 252 43 Průhonice, Czech Republic Czech Academy of Sciences Průhonice Czech Republic; 2 Institute of Clinical Biochemistry and Diagnostics, University Hospital Hradec Králové, 500 05 Hradec Králové, Czech Republic University Hospital Hradec Králové Hradec Králové Czech Republic; 3 Westerdijk Fungal Biodiversity Institute, 3584 CT Utrecht, Netherlands Westerdijk Fungal Biodiversity Institute Utrecht Netherlands

**Keywords:** *
Ascomycota
*, *
Dactylaria
*, DNA barcode, holoblastic-denticulate conidiogenesis, new taxa, phylogenetics, repository, saprobic, systematics

## Abstract

Public repositories of living fungal strains provide essential reference points and support diverse scientific outcomes. Current best practices for preserving fungal strains emphasise the generation of DNA barcodes and the management of comprehensive metadata. However, challenges arise when type material or authentic reference strains are lacking, as this prevents direct comparison of DNA barcodes and forces identifications to rely solely on morphology. This problem is particularly pronounced for strains deposited during the pre-molecular era, especially those belonging to species with simple or convergent morphologies. In this study, we re-examined seven strains deposited in a public culture collection under the name *Pleurophragmium parvisporum*, including synonymous designations. Our approach combined cultivation experiments, comparative morphological analyses, multi-locus phylogenetic reconstruction of six nuclear markers, and biogeographic assessments. Our analyses revealed that these strains are scattered across four distinct families or orders in three classes. Two strains belong to *Thysanorea* (*Chaetothyriales*, *Eurotiomycetes*): *T. acropleurogena***sp. nov.** and a sterile strain identified as the already known *T. melanica*. Two other strains were resolved within *Wongia* (*Papulosaceae incertae sedis*, *Sordariomycetes*) and introduced as *W. pallidopolaris***sp. nov.** and *W. rhachidophora***sp. nov.** Finally, two strains represent novel taxa within the *Tubeufiales (Dothideomycetes)*, described here as *Zaanenomyces hilifer***sp. nov.** and *Skoliomycella flava***gen. et sp. nov.** Of the seven examined strains, only one conformed to the species concept of *P. parvisporum* and is here regarded as its reference strain. The phylogenetic analyses resolved *P. parvisporum* within *Neomyrmecridium* (*Myrmecridiales*, *Sordariomycetes*). Consequently, *Neomyrmecridium* was reduced to synonymy of *Pleurophragmium*, leading to the proposal of 11 new combinations (*P. asiaticum***comb. nov.**, *P. asymmetricum***comb. nov.**, *P. fusiforme***comb. nov.**, *P. gaoligongense***comb. nov.**, *P. guizhouense***comb. nov.**, *P. luguense***comb. nov.**, *P. naviculare***comb. nov.**, *P. pteridophytophilum***comb. nov.**, *P. septatum***comb. nov.**, *P. sichuanense***comb. nov.**, and *P. sorbicola***comb. nov.**), and two new names (*P. fluviale***nom. nov.** and *P. jiulongheense***nom. nov.**). In addition, three species formerly placed in *Uncispora* are transferred to *Thysanorea*, with new combinations proposed based on congruent morphology and multi-locus phylogenetic evidence: *T. hainanensis***comb. nov.**, *T. sinensis***comb. nov.**, and *T. wuzhishanensis***comb. nov.** This study refines the generic limits of *Pleurophragmium* and morphologically similar genera and reveals several previously unrecognised lineages. It highlights how misinterpretation of subtle morphological features may lead to strains being misidentified and deposited under incorrect names in public collections, where they risk perpetuating taxonomic errors.

## ﻿Introduction

Public culture collections play a critical role in mycological research by serving as sources of reference material for taxonomy, evolutionary studies, ecology, biotechnology and pathology. They provide essential reference points that offer reliable information that is critical for data-driven research. As Hawksworth (2004) emphasised, such collections are more accurately termed genetic resource collections, reflecting their function as repositories of heritable biological diversity.

Fungi are one of the most species-rich yet understudied groups of organisms on Earth. Recent estimates place global fungal diversity between 1.5 to 3.2 million species, with a working mean of approximately 2.53 million (Niskanen et al. 2023). In contrast, only about 204 040 species have been scientifically documented, representing roughly 8% of the estimated mean (Index Fungorum, accessed on 10 September 2025). The discrepancy between known and estimated diversity is even more pronounced in ex situ conservation efforts, such as culture collections, cryobanks, and germplasm storages. Some public collections are skewed toward a limited number of economically or medically important taxa, while vast lineages, especially of non-lichenised, microfungal lineages, remain poorly represented or absent. Lofgren and Stajich (2021) highlighted the need to address the ‘invisible’ fungal biodiversity crisis and positioned culture collections as key tools in fungal conservation and systematics.

The World Data Centre for Microorganisms (gcm.wdcm.org, accessed on 10 September 2025) currently lists 588 984 strains of microorganisms, including fungi, yeasts, and bacteria, representing 57 219 species preserved across 158 culture collections worldwide. The proportion of fungal species maintained in culture collections is widely recognised to be low. Earlier estimates suggest that fewer than 20 000 fungal species are preserved ex situ (Hawksworth 2004). Among existing repositories, the Westerdijk Fungal Biodiversity Institute in Utrecht, the Netherlands, maintains the most comprehensive and globally recognised collection over 130 000 strains of microorganisms, of which more than 100 000 are fungi, including yeasts, corresponding to approximately 25 000 species. This extensive archive serves as the principal repository for nomenclatural types and authentic reference strains. It provides essential material for taxonomic, ecological, and biotechnological research.

The effectiveness and reliability of the culture collections depend heavily on accurate species identification and comprehensive metadata. These collections are commonly used under the assumption that deposited strains are correctly identified, especially when they are assigned to known species. Before molecular tools became available, strain identification relied mainly on morphology. Verification practices varied between institutions, and many names were accepted without re-examination. With the increasing accessibility of DNA sequencing, however, routine generation of DNA barcodes for newly deposited strains has become feasible and is now being implemented in various collections (Vu et al. 2016, 2019). In parallel, advances in data management systems (Reimer and Yurkov 2022) are improving the reliability and traceability of reference materials. Moreover, comprehensive monographic studies are being conducted to achieve better species resolution, particularly in genera of medical, industrial, or agricultural importance, such as *Acremonium*, *Alternaria*, *Aspergillus*, *Ceratocystis*, *Fusarium*, *Penicillium*, *Phoma*, and others (de Gruyter et al. 2013; Woudenberg et al. 2013, 2015; de Beer et al. 2014; Chen et al. 2017; Houbraken et al. 2020; Sandoval-Denis et al. 2025).

However, re-identification of historical strains in public collections, especially saprobic fungi, is still not routinely performed. Some strains deposited before the molecular era remain in collections under unverified names, and these may conceal considerable phylogenetic diversity, as demonstrated in the present study. Misidentifications, especially of morphologically defined isolates, can have cascading effects in systematics, phylogenetics, biotechnology, and ecology. In response, the mycological community advocates for standardization, molecular verification, and proper voucher deposition as essential pillars for maintaining the integrity and reproducibility of fungal science (Hawksworth 2004; Aime et al. 2021; Lofgren and Stajich 2021).

Nevertheless, even the implementation of routine DNA-based authentication may prove insufficient if reference or ex-type strains of the target species are unavailable, which is often the case. Under these circumstances, the traditional approach of carefully validating the morphology of obtained strains, guided by the principle of ‘trust, but verify’, remains essential for ensuring the reliability and success of any taxonomic or systematic research.

In this study, we present specific cases encountered during our own research, which illustrate how unverified identifications of strains in public collections can obscure true phylogenetic relationships and hinder taxonomic clarity. A notable example is provided by the studies of Shenoy et al. (2006, 2010), where reliance on misidentified living strains, without morphological verification by the authors, triggered a cascade of erroneous conclusions, ultimately affecting the outcomes of several subsequent systematic investigations. Using partial nuclear large subunit (LSU) rDNA sequences, Shenoy et al. (2010) proposed that the genus *Spadicoides* was polyphyletic. The genus, typified by *S. bina*, was established by Hughes (1958) to accommodate saprobic, primarily lignicolous, dematiaceous hyphomycetes. To support their hypothesis of polyphyly, Shenoy et al. (2010) analysed four available strains: *Spadicoides atra*CBS 489.77, *S. bina*CBS 113708, *S. verrucosa* ex-type CBS 128.86, and *S. xylogena*CBS 310.31. Although *S. atra* (GenBank accession: EF204506) and *S. bina* (GenBank accession: EF204507) are morphologically similar, phylogenetic analysis did not support their congeneric placement. The strain of *S. atra* formed a sister relationship to three species of *Lentomitella* (Réblová et al. 2018), whereas *S. bina* was placed within the *Porosphaerella* clade (Müller and Samuels 1982).

Hernández-Restrepo et al. (2017) accepted the conclusions of Shenoy et al. (2010), recognising *Spadicoides* as a member of the *Cordanaceae*, where *Porosphaerella* and its asexual morph *Cordana* belong, and segregating *S. atra* from *Spadicoides* into a newly established genus, *Xenospadicoides*. In addition, a new order, *Xenospadicoidales*, was introduced to accommodate *Lentomitella* and other spadicoides-like taxa. Using freshly collected material, Réblová et al. (2018) obtained *S. bina*CBS 137794 in axenic culture by isolating ascospores of an unidentified lentomitella-like species. Their multi-locus phylogenetic analyses confirmed that *S. bina*, *S. atra*, and other *Spadicoides* species are congeneric. Based on this evidence, the genus *Spadicoides* was accepted as a member of the *Xenospadicoidales*, and *Xenospadicoides* was reduced to its synonymy. However, the identity of the strain *S. bina*CBS 113708, previously placed within the *Cordanaceae* by Shenoy et al. (2010), remained unresolved.

*Spadicoides bina* and *Cordana pauciseptata* (Preuss 1851) are morphologically strikingly similar, both produce 1-septate, brown, ellipsoidal, acropleurogenous conidia of comparable size. However, they differ fundamentally in their mode of conidiogenesis. In *Spadicoides*, conidiogenous cells are tretic, whereas in *Cordana*, conidia are borne on denticles on holoblastic conidiogenous cells from terminal and intercalary (nodal) swellings. Detailed examination and comparison of the original voucher specimen housed in the Uppsala herbarium, the original dried culture and the derived living strain CBS 113708, both deposited in the CBS culture collection, confirmed that the fungus in question is, in fact, *C. pauciseptata* (Réblová et al. 2018). It is evident that the strain was initially misidentified, yet the deposition of all related material in public collections ultimately enabled accurate identification.

Another example concerns the misidentification of a strain of *Bahusutrabeeja dwaya* (MTCC 9680), the type species of the genus, by Shenoy et al. (2010). It was placed within the *Botryosphaeriales (Dothideomycetes)*. The authors were unable to account for this placement morphologically and discussed the apparent disparity between the phylogenetic and morphological data. Based on the ex-type strain, the species is currently assigned to *Codinaea* within the *Chaetosphaeriales* (Réblová et al. 2021). A similar case involves the misidentification of a non-type strain of *Linkosia multiseptatum* (HKUCC 10825), reported by Shenoy et al. (2006) as a member of the *Rhamphoriales*. More recent molecular data from the ex-type strain of *L. multiseptatum* (CGMCC 3.20786; Wu and Diao 2022) have placed *Linkosia* within the *Chaetosphaeriales*, together with other morphologically similar sporidesmium-like fungi.

This study is focused on the case of *Pleurophragmium parvisporum* and re-evaluates the strains deposited under this name to clarify the species’ identity and assess the broader implications of strain misidentification in public collections. *Pleurophragmium* (Costantin 1888), typified by *P. bicolor* (= *P. parvisporum*, Holubová-Jechová 1972), was introduced for dematiaceous hyphomycetes. It is characterised by transversely septate, hyaline conidia that become subhyaline or pale brown at maturity, borne terminally and laterally on denticles on holoblastic conidiogenous cells, arising from simple, macronematous conidiophores. De Hoog and von Arx (1974) transferred *Pleurophragmium* to *Dactylaria* (Saccardo 1880) based on overlapping conidial morphology, and de Hoog (1985) later accommodated it as Dactylaria sect. Pleurophragmium. The concept of this species was primarily derived from observations of two strains, CBS 531.73 and CBS 770.83, which were used to describe both species-level traits and colony morphology (de Hoog 1985).

*Pleurophragmium parvisporum* is represented by seven strains in the CBS culture collection. The only available molecular data for *P. parvisporum* consist of a partial LSU sequence from the non-type strain CBS 531.73 (GenBank accession: EU107296; Bhilabutra et al., unpublished), which was generated without prior morphological verification. In a phylogenetic analysis by Réblová (2009), the placement of *Rhodoveronaea* and morphologically similar genera with holoblastic-denticulate conidiogenesis was examined using homologous LSU sequences representing species from 21 orders and families across three fungal classes. *Pleurophragmium parvisporum*CBS 531.73 grouped with *Papulosa amerospora* AFTOL-ID 748 (Spatafora et al. 2006) in the *Sordariomycetes*. Based on these results, Réblová (2009) excluded *Pleurophragmium* from *Dactylaria* and tentatively re-evaluated its placement within the *Papulosaceae*. However, the author noted that this decision was made in the absence of verified original material.

In addition to *Dactylaria*, *Pleurophragmium* also shows striking morphological similarity to *Neomyrmecridium* (Crous et al. 2018a). *Neomyrmecridium*, based on *N. septatum*, currently encompassing 13 species, is characterised by dematiaceous, macronematous, erect, unbranched conidiophores, holoblastic-denticulate conidiogenous cells, and solitary, fusoid-ellipsoidal, obovoid or naviculate, transversely septate conidia that are initially hyaline and become pale brown with age, and have a mucoid sheath. Nevertheless, the close morphological similarity between the two genera has largely been overlooked in studies proposing new *Neomyrmecridium* species (e.g. Crous et al. 2018a, b; Serano et al. 2020; Xu et al. 2023; Zhang et al. 2024, 2025; Cao et al. 2025).

During a revision of the genus *Pleurophragmium*, we aimed to clarify the systematic position of *P. parvisporum*, a morphologically distinctive yet poorly understood taxon. Although it has been reported from temperate regions of Asia (Matsushima 1975), North America (Wang 2010), and Europe (Ellis 1968, 1971; Holubová-Jechová 1972), we were unable to recollect it in nature. Moreover, our recent examination of the strain CBS 531.73 raised suspicion that it may be misidentified. In response, we acquired seven strains of this species available in the CBS culture collection. These isolates were identified based on morphological characteristics at the time of their deposition. Although ITS barcodes are now available for these strains, the absence of ex-type or verified strain of *P. parvisporum* makes accurate identification impossible without manual validation.

In this study, we present a comprehensive investigation of *P. parvisporum*, with particular emphasis on clarifying the identity of seven strains obtained from the CBS culture collection. Our approach combined cultivation experiments, comparative morphological analyses, multi-locus phylogenetic reconstruction using six nuclear markers and biogeographic assessments. The results revealed unexpected phylogenetic diversity among these strains, which were shown to represent novel and previously known species, distributed across several genera, spanning three fungal classes.

## ﻿Materials and methods

### ﻿Isolates and morphological studies

Living strains were sourced from the Westerdijk Fungal Biodiversity Institute (**CBS**) at Utrecht, the Netherlands. Strains CBS 122759, CBS 440.70, and CBS 862.68 were originally deposited as *Pleurophragmium simplex* (a synonym of *P. parvisporum*) and later reassigned to *Dactylaria parvispora* or *Dactylaria* sp. Strain CBS 531.73 was deposited as *P. parvisporum* and subsequently placed under *D. parvispora*, while strains CBS 215.96, CBS 770.83, and CBS 113561 were deposited directly as *D. parvispora*. Dried cultures were deposited in the CBS Fungarium, while additional herbarium specimens of *P. parvisporum* were deposited in the Herbarium of the Institute of Botany (**PRA**), Czech Academy of Sciences at Průhonice, Czech Republic. Biogeographic assessments were conducted using environmental ITS datasets retrieved from the GlobalFungi database (Větrovský et al. 2020). Newly described taxa were registered in MycoBank (Crous et al. 2004). Details of the strains examined, including their origin and GenBank accession numbers of the generated sequences, are summarised in Table 1.

**Table 1.** Species, isolate information and new sequences determined for this study (in bold) and additional sequences retrieved from GenBank.

Organism	Strain	Status*	Host	Substrate	Country	GenBank accessions						Reference
						ITS	LSU	SSU	*rpb2*	*tef1*	*tub2*	
*Pleurophragmium simplex*	CBS 770.83		unidentified	dead twigs	Japan	**PX283736**	**PX283746**	**PX283743**	**PX310214**	**PX310206**	–	This study
*Skoliomycella flava*	CBS 122759	T	unidentified	plant debris	Portugal	**PX283737**	**PX283747**	–	**PX310215**	**PX310207**	–	This study
*Thysanorea acropleurogena*	CBS 215.96	T	unidentified	wood	Papua New Guinea	**PX283738**	**PX283748**	–	–	–	**PX310220**	This study
*Thysanorea melanica*	CBS 862.68		n/a	wheat field soil	Netherlands	**PX283739**	**PX283749**	–	–	–	**PX310221**	This study
*Wongia pallidopolaris*	CBS 440.70	T	n/a	sandy soil	Netherlands	**PX283740**	**PX283750**	**PX283744**	**PX310216**	**PX310208**	–	This study
*Wongia rhachidophora*	CBS 531.73	T	*Bambusa* sp.	dead leaf	India	**PX283741**	**PX283751**	**PX283745**	**PX310217**	**PX310209**	–	This study
*Zaanenomyces hilifer*	CBS 113561	T	*Carex* sp.	litter	Iran	**PX283742**	**PX283752**	–	**PX310218**	**PX310210**	–	This study
*Zaanenomyces moderatricis-academiae*	CBS 148315	T	*Juncus inflexus*	dead culm	Netherlands	OK664723	OK663762	–	OK651167	**PX310211**	–	Crous et al. 2021
*Zaanenomyces quadripartis*	CBS 148310	T	*Juncus effusus*	dead culm	Netherlands	OK664721	OK663760	–	**PX310219**	**PX310212**	–	Crous et al. 2021
*Zaanenomyces versatilis*	CBS 148312	T	*Juncus inflexus*	dead culm	Netherlands	OK664730	OK663769	–	–	**PX310213**	–	Crous et al. 2021

*T denotes ex-type culture.

Colony macromorphology was examined using an Olympus SZX12 dissecting microscope (Olympus America, Inc., Melville, NY, USA). Microscopic preparations were mounted in 90% lactic acid, water, or Melzer’s reagent, with measurements taken from specimens in Melzer’s reagent. Conidial dimensions are presented as mean ± standard deviation (SD) based on 20–25 measurements. Micromorphological traits were studied with an Olympus BX51 light microscope, and micrographs were captured using an Olympus DP75 camera operated via Olympus cellSens Dimension software v. 4.3. Colony images were taken with a Canon EOS 77D digital camera equipped with a Canon EF 100 mm f/2.8L Macro IS USM lens (Canon Europe Ltd., Middlesex, UK), illuminated with 5500K 16W LED lights. All images were processed in Adobe Photoshop CS6 (Adobe Systems, San Jose, CA, USA).

To examine colony morphology, pigment production, and growth rates, strains were cultivated on four media: cornmeal dextrose agar (CMD) (cornmeal agar, Oxoid Limited, Basingstoke, UK, supplemented with 2% w/v dextrose), malt extract agar (MEA) (Oxoid), Modified Leonian’s agar (MLA) (Malloch 1981), oatmeal agar (OA), and potato-carrot agar (PCA) (Crous et al. 2019). To promote sporulation, strains were also grown on cornmeal agar (CMA) (Crous et al. 2019) supplemented with dried stems of *Urtica dioica* and incubated under alternating 12-h cycles of UV light and darkness. Colony features were recorded from 4-wk-old cultures incubated at 23 °C in darkness.

Scientific names of fungal genera reported in this study are abbreviated as follows: Botryosphaeria (B.), Camporesiomyces (C.), *Cancellidium (C.) Cordana (C.)*, Cyphellophora (C.), Helicoma (H.), Minimelanolocus (M.), Neomyrmecridium (N.), Pleurophragmium (P.), Pseudospiropes (P.), Skoliomycella (S.), Thysanorea (T.), Tubeufia (T.), Uncispora (U.), Wongia (W.), and Zaanenomyces (Z.).

### ﻿Molecular methods

Phylogenetic relationships were evaluated using six gene markers: internal transcribed spacer ITS1–5.8S–ITS2 (ITS) of the nuclear rDNA cistron (ITS barcode) (Schoch et al. 2012), nuclear large subunit (LSU) rDNA gene, the nuclear small subunit (SSU) rDNA gene, and three coding markers, i.e. the second largest subunit of RNA polymerase II (DNA-directed RNA polymerase) (*rpb2*), the intermediate section of the translation elongation factor 1-α (*tef1*), and β-tubulin (*tub2*) marked by exons 3−6. The LSU and SSU markers provide reliable phylogenetic resolution at the generic and higher taxonomic levels in fungi (e.g. Zhang et al. 2007; Schoch et al. 2009). The coding gene markers are recognised for their effectiveness in resolving interspecific relationships (Stielow et al. 2015; Meyer et al. 2019).

Protocols for DNA extraction from 2–4-d old cultures and PCR amplification of cited gene markers were conducted following the methods described by Réblová et al. (2022) and Réblová and Nekvindová (2023). Automated sequencing was carried out by Eurofins Genomics Europe Sequencing Service (Cologne, Germany). Analyses of raw sequence data and assembly of sequence contigs were performed using Sequencher v. 5.4.6 (Gene Codes Corp., Ann Arbor, MI, USA).

### ﻿Phylogenetic analyses

Preliminary similarity searches were performed using the BLASTn and megaBLAST algorithms with ITS and LSU sequences of all studied strains to identify their closest relatives. Corresponding ITS, LSU, SSU, *rpb2*, *tef1*, and *tub2* sequences of related taxa were then retrieved from GenBank sequence database at NCBI (Sayers et al. 2022) and used in subsequent analyses. Sequences of these species, together with their GenBank accession numbers and references, are listed in Table 2.

**Table 2.** Species, isolate information and sequences retrieved from GenBank.

Taxon	Strain	Status	GenBank Accession numbers						Reference
			ITS	LSU	SSU	*rpb2*	*tef1*	*tub2*	
*Acanthohelicospora pinicola*	MFLUCC 10-0116	T	KF301526	KF301534	–	–	KF301555	–	Boonmee et al. 2014
*Acanthostigma chiangmaiense*	MFLUCC 10-0125	T	JN865209	JN865197	–	–	KF301560	–	Boonmee et al. 2011, 2014
*Acanthostigma perpusillum*	UAMH 7237		AY916492	AY856892	–	–	–	–	Tsui et al. 2006
*Aciculomyces restrictus*	FMR 18994	T	ON009870	ON009950	–	–	–	ON667802	Torres-Garcia et al. 2023
*Aculeata aquatica*	MFLUCC 11-0529	T	MG922571	MG922575	–	–	–	–	Dong et al. 2018
*Atrokylindriopsis setulosa*	HMAS 245592	T	KP337330	KP337329	–	–	–	–	Ma et al. 2015
*Berkleasmium aquaticum*	MFLUCC 17-0049	T	KY790444	KY790432	–	MF535268	KY792608	–	Lu et al. 2017c, 2018b
*Berkleasmium fusiforme*	MFLUCC 17-1978	T	MH558693	MH558820	–	MH551007	MH550884	–	Lu et al. 2018a
*Berlesiella nigerrima*	MUCL 39954		AF050251	AF050251	–	–	–	–	Untereiner and Naveau 1999
*Boerlagiomyces macrosporus*	MFLUCC 12-0388		KU144927	KU764712	–	–	KU872750	–	Doilom et al. 2017
*Botryosphaeria agaves*	MFLUCC 10-0051		JX646790	JX646807	–	–	JX646855	–	Liu et al. 2012
*Botryosphaeria dothidea*	CBS 115476	E	KF766151	DQ678051	–	DQ677944	DQ767637	–	Schoch et al. 2006; Slippers et al. 2013
*Botryosphaeria wangensis*	HGUP 190007		MZ541933	MZ540051	–	OP321271	–	–	Zhang et al. 2021, 2022
*Brunneosporella aquatica*	HKUCC 3708		AF177154	AF132326	–	–	–		Ranghoo et al. 1999
*Camporesiomyces bhatii*	GMBCC 1120	T	PQ763360	PQ842543	–	PV388888	PV388894	–	Han et al. 2025
*C. bhatii*	GMBCC 1125		PQ763361	PQ842544	–	PV388889	PV388895	–	Han et al. 2025
*Camporesiomyces coffeae*	GMBCC 1130	T	PQ763358	PQ842545	–	PV388890	PV388896	–	Han et al. 2025
*C. coffeae*	GMBCC 1131		PQ763359	PQ842546	–	PV388891	PV388897	–	Han et al. 2025
*Camporesiomyces mali*	KUMCC 19-0216	T	MN792813	MN792811	–	–	MN794018	–	Hyde et al. 2020
*Camporesiomyces patagoniensis*	BBB MVB 573	T	JN127358	JN127359	–	–	–	–	Sánchez et al. 2012
*Camporesiomyces puerensis*	GMBCC 1113	T	PQ763356	PQ842541	–	PV388886	PV388892	–	Han et al. 2025
*C. puerensis*	GMBCC 1114		PQ763357	PQ842542	–	PV388887	PV388893	–	Han et al. 2025
*Camporesiomyces vaccinii*	CBS 216.90	T	AY916486	AY856879	–	–	–	–	Tsui et al. 2006
*Cancellidium atrobrunneum*	MFLUCC 20-0100	T	MT422724	MT422740	MT422726	–	MT436438	–	Hyde et al. 2021
*Cancellidium cinereum*	MFLUCC 18-0424	T	MT370353	MT370363	MT370351	MT370486	MT370488	–	Hyde et al. 2021
*Cancellidium griseonigrum*	MFLUCC 17-2117	T	MT370354	MT370364	MT370352	MT370487	–	–	Hyde et al. 2021
*Capronia camelliae-yunnanensis*	CGMCC 3.19061	T	MH807377	MH807378	–	–	–	–	Phookamsak et al. 2019
*Capronia kleinmondensis*	CBS 122671	T	MH863226	MH874753	–	–	–	–	Vu et al. 2019
*Capronia leucadendri*	CBS 122672	T	MH863227	MH874754	–	–	–	–	Vu et al. 2019
*Capronia lijiangensis*	CGMCC 3.20501	T	OK487581	OK487580	–	–	–	–	Phukhamsakda et al. 2022
*Capronia pilosella*	AFTOL-ID 657		DQ826737	DQ823099	–	–	–	–	James et al. 2006
*Cladophialophora boppii*	CBS 126.86	T	EU103997	NG_058762	–	–	–	–	Gueidan et al. 2008
*Cladophialophora carrionii*	CBS 114393		KF928452	KF928516	–	–	–	EU137151	de Hoog et al. 2007; Attili-Angelis et al. 2014
*C. carrionii*	CBS 160.54	T	AB109177	LC192080	–	–	–	EU137201	Abliz et al. 2004; de Hoog et al. 2007; Kiyuna et al. 2018
*Cladophialophora floridana*	NRRL 66282	T	AB986343	AB986343	–	–	–	–	Obase et al. 2016
*Cladophialophora chaetospira*	CBS 114747		EU035403	KF928514	–	–	–	KF928578	Crous et al. 2007; Attili-Angelis et al. 2014
*Cladophialophora matsushimae*	MFC-1P384	T	FN549916	FN400758	–	–	–	–	Koukol 2010
*Cladophialophora mycetomatis*	CBS 122637	T	FJ385276	LC192076	–	–	–	–	Badali et al. 2008; Kiyuna et al. 2018
*Cladophialophora yegresii*	CBS 114405	T	EU137322	KX822323	–	–	–	EU137209	de Hoog et al. 2007; Vasse et al. 2017
*Cyphellophora aestiva* ^a^	CBS 228.86	T	KC455244	KC455257	–	–	–	KC455227	Réblová et al. 2013
*Cyphellophora laciniata*	CBS 190.61	T	EU035416	FJ358239	–	–	–	JQ766329	Crous et al. 2007; Gueidan et al. 2008; Feng et al. 2014
*Cyphellophora suttonii*	CBS 449.91	T	KC455243	KC455256	–	–	–	KC455226	Réblová et al. 2013
*Dematiohelicomyces helicosporus*	MFLUCC 16-0213	T	KX454169	KX454170	–	MF535258	KY117035	–	Hyde et al. 2016; Lu et al. 2017b, 2018b
*Dematiohelicosporum guttulatum*	MFLUCC 17-2011	T	MH558705	MH558833	–	MH551021	MH550896	–	Lu et al. 2018a
*Dematiotubeufia chiangraiensis*	MFLUCC 10-0115	T	JN865200	JN865188	–	–	KF301551	–	Boonmee et al. 2011, 2014
*Dictyospora thailandica*	MFLUCC 16-0001	T	KY873627	KY873622	–	MH551023	KY873286	–	Brahamanage et al. 2017; Lu et al. 2018a
*Exophiala angulospora*	CBS 482.92	T	JF747046	MH874033	–	–	–	JN112426	de Hoog et al. 2011; Vu et al. 2019
*Exophiala castellani*	CBS 158.58	T	KF928458	MH869272	–	–	–	KF928586	Attili-Angelis et al. 2014; Vu et al. 2019
*Exophiala dermatitidis*	CBS 207.35	T	AF050269	KF928508	–	–	–	KF928572	Untereiner and Naveau 1999; Attili-Angelis et al. 2014
*Exophiala equina*	FMR 18335		ON009852	ON009932	–	–	–	ON491590	Torres-Garcia et al. 2023
*Exophiala heteromorpha*	CBS 232.33	T	AY857524	MH866871	–	–	–	–	Prenafeta-Boldú et al. 2006; Vu et al. 2019
*Exophiala jeanselmei*	CBS 507.90	T	MH862234	MH873915	–	–	–	–	Vu et al. 2019
*Exophiala mesophila*	CBS 402.95	T	JF747111	KX712349	–	–	–	JN112476	de Hoog et al. 2011; Teixeira et al. 2017
*Exophiala nigra*	CBS 535.94	T	MH86248	KX712353	–	–	–	–	Teixeira et al. 2017; Vu et al. 2019
*Exophiala nishimurae*	CBS 101538	T	AY163560	KX712351	–	–	–	JX482552	de Hoog et al. 2003; Woo et al. 2013; Teixeira et al. 2017
*Exophiala pisciphila*	CBS 537.73	T	NR_121269	MH872483	–	–	–	JN112493	de Hoog et al. 2011; Schoch et al. 2014; Vu et al. 2019
*Exophiala prototropha*	CBS 534.94		OR371992	–	–	–	–	–	Unpublished
*Exophiala radicis*	FMR 18645		ON009857	ON009937	–	–	–	ON667789	Torres-Garcia et al. 2023
*Exophiala salmonis*	CBS 157.67	T	MH858932	MH870616	–	–	–	JN112499	de Hoog et al. 2011; Vu et al. 2019
*Fluminicola aquatica*	MFLUCC 15-0962	T	MF374357	MF374366	MF374374	–	MF370960	–	Zhang et al. 2017
*Fluminicola saprophytica*	MFLUCC 15-0976	T	NR_153493	MF374367	MF374375	MF370954	MF370956	–	Zhang et al. 2017
*Fluminicola striata*	MFLUCC 18-0990	T	MW286496	MW287770	–	–	–	–	Dong et al. 2021
*Fonsecaea monophora*	CBS 289.93		AY366925	–	–	–	–	EU938554	de Hoog et al. 2004; Najafzadeh et al. 2009
*Fonsecaea nubica*	CBS 269.64	T	EU938592	–	–	–	–	EU938574	Najafzadeh et al. 2009
*Fonsecaea pedrosoi*	CBS 271.37	N	AB114127	KJ930166	–	–	–	EU938559	de Azevedo et al. 2015; Najafzadeh et al. 2009
*Fonsecaea pugnacius*	CBS 139214	T	KR706553	KR706549	–	–	–	KR706547	de Azevedo et al. 2015
*Helicangiospora lignicola*	MFLUCC 11-0378	T	KF301523	KF301531	–	–	KF301552	–	Boonmee et al. 2014
*Helicoarctatus aquaticus*	MFLUCC 17-1996	T	MH558707	MH558835	–	MH551024	MH550898	–	Lu et al. 2018a
*Helicodochium aquaticum*	MFLUCC 17-2016	T	MH558709	MH558837	–	MH551026	MH550900	–	Lu et al. 2018a
*Helicohyalinum aquaticum*	MFLUCC 16-0014		MH558711	MH558839	–	MH551028	MH550902	–	Lu et al. 2018a
*Helicohyalinum infundibulum*	MFLUCC 16-1133	T	MH558712	MH558840	–	MH551029	MH550903	–	Lu et al. 2018a
*Helicoma brunneisporum*	MFLUCC 17-1983	T	MH558714	MH558842	–	MH551031	MH550905	–	Lu et al. 2018a
*Helicoma longisporum*	MFLUCC 17-1997	T	MH558720	MH558846	–	MH551037	MH550911	–	Lu et al. 2018a
*Helicomyces hyalosporus*	MFLUCC 17-0051	T	MH558731	MH558857	–	MH551047	MH550922	–	Lu et al. 2018a
*Helicomyces torquatus*	MFLUCC 16-0217		MH558732	MH558858	–	MH551048	MH550923	–	Lu et al. 2018a
*Helicosporium aquaticum*	MFLUCC 17-2008	T	MH558733	MH558859	–	MH551049	MH550924	–	Lu et al. 2018a
*Helicosporium flavisporum*	MFLUCC 17-2020	T	MH558734	MH558860	–	MH551050	MH550925	–	Lu et al. 2018a
*Helicosporium luteosporum*	MFLUCC 16-0226	T	KY321324	KY321327	–	MH551056	KY792601	–	Lu et al. 2017a; 2018a
*Helicosporium setiferum*	MFLUCC 17-1994	T	MH558735	MH558861	–	MH551051	MH550926	–	Lu et al. 2018a
*Helicosporium vegetum*	CBS 254.75		–	DQ470982	–	DQ470934	DQ471105	–	Spatafora et al. 2006
*Helicosporium vesicarium*	MFLUCC 17-1795	T	MH558739	MH558864	–	MH551055	MH550930	–	Lu et al. 2018a
*Helicotubeufia guangxiensis*	MFLUCC 17-0040	T	MH290018	MH290023	–	MH290033	MH290028	–	Liu et al. 2018b
*Helicotubeufia hydei*	MFLUCC 17-1980	T	MH290021	MH290026	–	MH290036	MH290031	–	Liu et al. 2018b
*Chlamydotubeufia cylindrica*	MFLUCC 16-1130	T	MH558702	MH558830	–	MH551018	MH550893	–	Lu et al. 2018a
*Chlamydotubeufia krabiensis*	MFLUCC 16-1134	T	KY678767	KY678759	–	MF535261	KY792598	–	Hyde et al. 2017; Lu et al. 2018b
*Kamalomyces bambusicola*	MFLU 11-0228	T	–	MF506880	–	–	–	–	Phookamsak et al. 2017
*Kamalomyces thailandicus*	MFLUCC 13-0233	T	MF506884	MF506882	–	–	MF506886	–	Phookamsak et al. 2017
*Manoharachariella tectonae*	MFLUCC 12-0170	T	KU144935	KU764705	–	–	KU872762	–	Doilom et al. 2017
*Marinophialophora garethjonesii*	MFLUCC 16-1449	T	KY305174	KY305176	–	–	–	–	Li et al. 2018
*Melanchlenus eumetabolus*	CBS 264.49	T	EU041812	EU041869	–	–	–	–	Arzanlou et al. 2007
*Melanoctona tectonae*	MFLUCC 12-0389	T	KX258778	KX258779	–	–	–	–	Tian et al. 2016
*Muripulchra aquatica*	KUMCC 15-0276		KY320534	KY320551	–	MH551058	KY320564	–	Luo et al. 2017; Lu et al. 2018a
*Myrmecridium banksiae*	CBS 132536	T	JX069871	JX069855	–	–	–	–	Crous et al. 2012
*Myrmecridium dactylidis*	CBS 148281	T	OK664729	OK663768	–	–	–	–	Crous et al. 2021
*Myrmecridium flexuosum*	CBS 398.76	T	EU041768	EU041825	–	–	–	–	Arzanlou et al. 2007
*Myrmecridium fluviae*	CNUFC-YR61-1	T	KX839678	KX839677	–	–	–	–	Tibpromma et al. 2017
*Myrmecridium hiemale*	CBS 141017	T	NR_155370	KU302612	–	–	–	–	Peintner et al. 2016
*Myrmecridium hydei*	MFLUCC 23-0217	T	OR500543	OR500545	–	–	–	–	Asghari et al. 2023
*Myrmecridium iridis*	CBS 139917	T	KR476744	KR476777	–	–	–	–	Crous et al. 2015a
*Myrmecridium junci*	CBS 148274	T	OK664725	OK663764	–	–	–	–	Crous et al. 2021
*Myrmecridium juncicola*	CBS 148316	T	OK664731	OK663770	–	–	–	–	Crous et al. 2021
*Myrmecridium juncigenum*	CBS 148268	T	OK664735	OK663774	–	–	–	–	Crous et al. 2021
*Myrmecridium mexiae*	BRIP 69701	T	OM417274	OM333588	–	–	–	–	Tan and Steinrucken 2024
*Myrmecridium montsegurinum*	PRM 934684	T	KT991674	KT991664	–	–	–	–	Réblová et al. 2016b
*Myrmecridium normannianum*	CBS 149439	T	OP675888	OP681177	–	–	–	–	Tan et al. 2022
*Myrmecridium obovoideum*	HGUP 0314	T	KC136140	KC136139	–	–	–	–	Jie et al. 2013
*Myrmecridium phragmiticola*	CPC 36367	T	MT373366	MT373349	–	–	–	–	Crous et al. 2020
*Myrmecridium phragmitis*	CBS 131311	T	JQ044425	JQ044444	–	–	–	–	Crous et al. 2011
*Myrmecridium pulvericola*	DAOMC 250405	T	KU309312	KU309313	–	–	–	–	Crous et al. 2016
*Myrmecridium sambuci*	CBS 148444	T	OK664707	OK663746	–	–	–	–	Crous et al. 2021
*Myrmecridium schulzeri*	CBS 325.74	T	EU041775	EU041832	–	–	–	–	Arzanlou et al. 2007
*Myrmecridium spartii*	CBS 140006	T	KR611884	KR611902	–	–	–	–	Crous et al. 2015b
*Myrmecridium splendidum*	GZCC 19-0549	T	MW133875	OP377931	–	–	–	–	Yang et al. 2023
*Myrmecridium submersum*	CGMCC 3.27410	T	PQ038339	PQ226145	–	–	–	–	Zhang et al. 2024
*Myrmecridium thailandicum*	CBS 136551	T	KF777169	KF777222	–	–	–	–	Crous et al. 2013
*Myrmecridium yunnanense*	GZAAS 23-0586	T	OR438389	OR438853	–	–	–	–	Tian et al. 2024
*Neoacanthostigma fusiforme*	MFLUCC 11-0510	T	KF301529	KF301537	–	–	–	–	Boonmee et al. 2014
*Neodictyospora karsti*	GZAAS 19-1751	T	–	OP099534	–	OR146925	OR140408	–	Zhang et al. 2023b
*Neohelicoma fagacearum*	MFLUCC 11-0379	T	KF301524	KF301532	–	–	KF301553	–	Boonmee et al. 2014
*Neohelicomyces aquaticus*	MFLUCC 16-0993	T	KY320528	KY320545	–	MH551066	KY320561	–	Luo et al. 2017; Lu et al. 2018a
*Neohelicomyces grandisporus*	KUMCC 15-0470	T	KX454173	KX454174	–	MH551067	–	–	Luo et al. 2017; Lu et al. 2018a
*Neohelicosporium guangxiense*	MFLUCC 17-1522	T	MF467922	MF467935	–	MF535278	MF535248	–	Lu et al. 2018b
*Neohelicosporium parvisporum*	MFLUCC 17-1523	T	MF467926	MF467939	–	MF535282	MF535252	–	Lu et al. 2018b
*Neoherpotrichiella juglandicola*	CBS 147585	T	ON110815	ON111439	–	–	–	ON181438	Crous et al. 2022
*Neochlamydotubeufia fusiformis*	MFLUCC 16-0016	T	MH558740	MH558865	–	MH551059	MH550931	–	Lu et al. 2018a
*Neochlamydotubeufia khunkornensis*	MFLUCC 10-0118	T	JN865202	JN865190	–	–	KF301564	–	Boonmee et al. 2011, 2014
*Neomanoharachariella aquatica*	CGMCC 3.23539	T	OP184074	OP184063	–	OP186058	OP186047	–	Li et al. 2022
*Neotubeufia krabiensis*	MFLUCC 16-1125	T	MG012031	MG012024	–	MG012017	MG012010	–	Chaiwan et al. 2017
*Neoveronaea sinensis*	JAUCC M0840-1	T	OM832969	OM832968	–	–	–	–	Qiu et al. 2022
*Papulosa amerospora*	AFTOL-ID 748		–	DQ470950	DQ470998	DQ470901	DQ471069	–	Spatafora et al. 2006
*Parahelicomyces hyalosporus*	CGMCC 3.23535		OP184073	OP184062	–	OP186057	OP186046	–	Li et al. 2022
*Parahelicomyces talbotii*	MFLUCC 17-2021		MH558765	MH558890	–	MH551091	MH550957	–	Lu et al. 2018a
*Phaeoannellomyces elegans*	CBS 101597		KF928443	KF928507	–	–	–	KF928571	Attili-Angelis et al. 2014
*Phialophora americana*	UAMH 10875	T	EU514696	EU514696	–	–	–	EU514712	Untereiner et al. 2008
*Phialophora chinensis*	BMU 01890	T	KF881964	KJ930093	–	–	–	KF971765	Li et al. 2017
*Phialophora verrucosa*	CBS 140325	E	KF881960	KJ930073	–	–	–	KF971761	Li et al. 2017
*Platytrachelon abietis*	CBS 125235		–	JX066703	JX066707	JX066698	–	–	Réblová 2013
*Pleurohelicosporium hyalinum*	GZCC 20-0489	T	OP377816	OP377915	–	OP473089	OP472996	–	Yang et al. 2023
*Pleurohelicosporium parvisporum*	MFLUCC 17-1982	T	MH558764	MH558889	–	MH551088	MH550956	–	Lu et al. 2018a
*Pleurophragmium fluviale* ^b^	MFLUCC 15-0366	T	–	MK849804	–	–	–	–	Luo et al. 2019
*P. fluviale* ^b^	S-001	P	MK828657	MK849805	–	–	–	–	Luo et al. 2019
*P. fluviale* ^b^	S-1158	P	MK828656	MK849803	–	–	–	–	Luo et al. 2019
*Pleurophragmium asiaticum*	CBS 145080	T	MK047444	MK047494	–	–	–	–	Crous et al. 2018a
*Pleurophragmium asymmetricum*	CCM-CIBE-H304	T	MN014057	MN014055	–	–	–	–	Serrano et al. 2020
*Pleurophragmium fusiforme*	CGMCC 3.27412	T	PQ038340	PQ226146	–	–	–	–	Zhang et al. 2024
*Pleurophragmium gaoligongense*	KUNCC 10794	T	OP326185	OP326197	–	–	–	–	Xu et al. 2023
*P. gaoligongense*	KUNCC 10795		OP326186	OP326198	–	–	–	–	Xu et al. 2023
*Pleurophragmium guizhouense*	GZCC 20-0008	T	MT002305	MT002307	–	–	–	–	Hyde et al. 2020
*Pleurophragmium luguense*	KUNCC 10796	T	OP326187	OP326199	–	–	–	–	Xu et al. 2023
*P. luguense*	KUNCC 10797		OP326188	OP326200	–	–	–	–	Xu et al. 2023
*Pleurophragmium naviculare*	GZCC 20-0484	T	OP377827	OP377927	–	–	–	–	Yang et al. 2023
*P. naviculare*	MFLUCC 19-0303		OP377828	OP377928	–	–	–	–	Yang et al. 2023
*Pleurophragmium pteridophytophilum*	KUNCC 23-13858	T	PQ671274	PQ671193	–	–	–	–	Zhang et al. 2025
*Pleurophragmium septatum*	CBS 145073	T	MK047442	MK047492	–	–	–	–	Crous et al. 2018a
*Pleurophragmium sichuanense*	HUEST 24.0068	T	PP407782	–	–	–	–	–	Chen et al. 2025
*Pleurophragmium sorbicola*	CBS 143433	T	MH107901	MH107948	–	–	–	–	Crous et al. 2018b
*Pleurophragmium jiulongheense* ^c^	KUNCC 23–15577	T	PQ143313	PQ143315	–	–	–	–	Cao et al. 2025
*Pseudostanjehughesia aquitropica*	MFLUCC 16-0569	T	MF077548	MF077559	MF077537	–	MF135655	–	Yang et al. 2018
*Pseudostanjehughesia lignicola*	MFLUCC 15-0352	T	MK828643	MK849787	–	MN124534	MN194047	–	Luo et al. 2019
*Pseudotubeufia hyalospora*	GZCC 22-2010	T	OR030840	OR030833	–	–	OR046677	–	Ma et al. 2023
*Pseudotubeufia laxispora*	GZCC 22-2011	T	OR030838	OR030831	–	OR046682	OR046675	–	Ma et al. 2023
*Rhinocladiella anceps*	CBS 181.65	IN	MH858534	EU041862	–	–	–	–	Arzanlou et al. 2007; Vu et al. 2019
*Rhinocladiella aquaspersa*	CBS 313.73	T	MH860689	MH872396	–	–	–	GU079660	Badali et al. 2010; Vu et al. 2019
*Rhinocladiella atrovirens*	CBS 317.33	AS	MH855447	MH866906	–	–	–	–	Vu et al. 2019
*Rhinocladiella phaeophora*	CBS 496.78	T	MH861169	MH872933	–	–	–	GU079661	Badali et al. 2010; Vu et al. 2019
*Thaxteriellopsis lignicola*	MFLUCC 10-0124	E	JN865208	JN865196	–	–	KF301561	–	Boonmee et al. 2011, 2014
*T. lignicola*	MFLUCC 16-0024		MH558767	MH558892	–	MH551093	MH550959	–	Lu et al. 2018a
*Thysanorea asiatica*	MFLUCC 15-0237	T	KR215604	KR215610	–	–	–	–	Liu et al. 2015
*Thysanorea cantrelliae*	CBS 145909	T	MN794376	MN794353	–	–	–	–	Hernández-Restrepo et al. 2020
*Thysanorea curvata*	MFLUCC 15-0259	T	KR215605	KR215609	–	–	–	–	Liu et al. 2015
*Thysanorea hainanensis*	YMF 1.040381	T	KX495642	–	–	–	–	–	Li et al. 2015
*Thysanorea melanica*	MFLUCC 15-0415	T	KR215608	KR215613	–	–	–	–	Liu et al. 2015
*Thysanorea nonramosa*	MFLUCC 17-2378	T	MH532971	MH532970	–	–	–	–	Wang et al. 2019
*Thysanorea obscura*	MFLUCC 15-0416		KR215606	KR215611	–	–	–	–	Liu et al. 2015
*Thysanorea papuana*	CBS 212.96	T	MH862572	MH875246	–	–	–	–	Vu et al. 2019
*T. papuana* ^d^	MFLUCC 15-0966	T	MG922572	MG922576	–	–	–	–	Dong et al. 2018
*Thysanorea rousseliana*	CBS 126086		MH863784	–	–	–	–	–	Vu et al. 2019
*Thysanorea seifertii*	CBS 145910	T	MN794377	MN794354	–	–	–	–	Hernández-Restrepo et al. 2020
*Thysanorea sinensis*	YMF 1.03683	T	KU173860	KU173860	–	–	–	–	Liu et al. 2018a
*Thysanorea submersa*	KUMCC 15-0206	T	KX789212	KX789215	–	–	–	–	Hyde et al. 2016
*Thysanorea thailandensis*	MFLUCC 15-0971	T	MG922573	MG922577	–	–	–	–	Dong et al. 2018
*Thysanorea wuzhishanensis*	YMF 1.04080	T	KU173859	KU558912	–	–	–	–	Liu et al. 2018a
*Thysanorea yunnanensis*	MFLUCC 15-0414	T	KR215607	KR215612	–	–	–	–	Liu et al. 2015
*Tubeufia eccentrica*	MFLUCC 17-1524	T	MH558782	MH558907	–	MH551108	MH550974	–	Lu et al. 2018a
*Tubeufia inaequalis*	MFLUCC 17-0053	T	MH558789	MH558914	–	MH551115	MH550982	–	Lu et al. 2018a
*Valentiella maceioensis*	BSS 376		MZ042488	MZ042486	–	–	–	–	Bezerra et al. 2022
*V. maceioensis*	T171 Tm12		KF614875	KF614875	–	–	–	–	Nepel et al. 2014
*Veronaea botryosa*	CBS 254.57	T	EU041816	EU041873	–	–	–	JN112505	Arzanlou et al. 2007; de Hoog et al. 2011
*V. botryosa ^e^*	CBS 572.90	T	MH862237	MH873920	–	–	–	–	Vu et al. 2019
*Veronaea compacta*	CBS 268.75	T	EU041819	EU041876	–	–	–	–	Arzanlou et al. 2007
*Veronaea japonica*	CBS 776.83	T	EU041818	EU041875	–	–	–	–	Arzanlou et al. 2007
*Wongia aquatica*	MFLUCC 18-1607	T	MK828645	MK849788	MK828312	MN124536	MN194048	–	Luo et al. 2019
*Wongia bambusae*	CGMCC 3.24360	T	OR822001	OR822017	OR822010	OR862129	OR873427	–	Yu et al. 2024
*W. bambusae*	KUNCC 24-17699		PQ571131	PQ573785	PQ571125	PQ591896	PQ591892	–	Wang et al. 2025
*Wongia bandungensis*	TBRC-BCC 95171	T	OQ121929	OQ121947	OQ121938	OQ116752	OQ116761	–	Manawasinghe et al. 2024
*W. bandungensis*	TBRC-BCC 95343	I	OQ121930	OQ121948	OQ121939	OQ116753	OQ116762	–	Manawasinghe et al. 2024
*Wongia ficherai*	BRIP 69019	T	OM230139	OM230140	–	OM162025	–	–	Crous et al. 2022
*Wongia flava*	CGMCC 3.25434	T	OR589341	OR769700	OR743229	OR820915	OR739187	–	Wang et al. 2024
*Wongia fusiformis*	DLUCC 1767		MZ420746	MZ420761	MZ420750	–	–	–	Bao et al. 2021
*W. fusiformis*	KUNCC 23-16632		PQ571134	PQ573788	PQ571128	PQ591898	PQ591895	–	Wang et al. 2025
*W. fusiformis*	MFLUCC 21-0028		MZ412517	MZ412529	MZ413273	–	MZ442690	–	Bao et al. 2021
*W. fusiformis*	MFLUCC 21-0032	T	MZ412515	MZ412527	MZ413271	–	MZ442689	–	Bao et al. 2021
*Wongia garrettii*	DAR 79637	T	KU850474	–	–	–	KU850467	–	Khemmuk et al. 2016
*Wongia griffinii*	BRIP 60377		KU850472	KU850470	–	–	KU850466	–	Khemmuk et al. 2016
*W. griffinii*	DAR 80512	T	KU850473	KU850471	–	–	–	–	Khemmuk et al. 2016
*Wongia guttulata*	KUNCC 24-17692	T	PQ571135	PQ573789	–	PQ591899	–	–	Wang et al. 2025
*Wongia miscanthi*	BCRC FU32062	T	LC822730	LC822732	LC822736	LC822734	LC822738	–	Kuo et al. 2024
*Wongia suae*	CGMCC 3.24295	T	OQ911478	OQ911483	OQ998925	OR039047	OR039046	–	Zhang et al. 2023a
*Zaanenomyces quadripartis*	CBS 148272		OK664722	OK663761	–	–	–	–	Crous et al. 2021
*Zaanenomyces versatilis*	CBS 149453		OQ990135	OQ990086	–	–	OQ989250	–	Crous et al. 2023

^a^ ex-type strain of *Cyphellophora vermispora*. ^b^ ex-type strain of *Neomyrmecridium aquaticum*. ^c^ ex-type strain of *Neomyrmecridium triseptatum*. ^d^ ex-type strain of *Thysanorea aquatica*. ^e^ ex-type strain of *Veronaea constricta*. *ex-holotype (T); ex-epitype (E); ex-isoneotype (IN); ex-neotype (N); ex-paratype (P) cultures; authenticated strain (AS).

Sequence alignments were generated with MAFFT v. 7.487 (Katoh and Standley 2013) via the CIPRES Science Gateway v. 3.3 (Miller et al. 2010) and adjusted manually in BioEdit v. 7.1.8 (Hall 1999) when necessary. The best nucleotide substitution models for each partition (ITS, LSU, SSU, *rpb2*, *tef1*, *tub2*) were selected under the Akaike Information Criterion using MrModeltest v. 2.4 (Nylander 2004). Phylogenetic reconstructions were performed with Maximum Likelihood (ML) and Bayesian Inference (BI) methods implemented in the CIPRES Science Gateway.

Maximum Likelihood (ML) analyses were carried out in RAxML-HPC v. 8.2.12 using the GTRCAT approximation (Stamatakis 2014), with branch support estimated from 1 000 non-parametric bootstrap replicates. Bayesian Inference (BI) analyses were performed with MrBayes v. 3.2.7 (Ronquist et al. 2012), running two independent searches under default parameters. The Bayesian Metropolis-coupled Markov chain Monte Carlo (B-MCMCMC) analyses were continued until the average standard deviation of split frequencies dropped below 0.01. Trees were sampled every 1 000 generations, with 25% discarded as burn-in. ML and BI trees were compared visually to identify topological conflicts among well-supported clades. Resulting phylogenies were visualised in FigTree v. 1.4.3 (Rambaut 2010) and SeaView v. 5.0.5 (Gouy et al. 2010) and subsequently edited in Microsoft PowerPoint and CorelDRAW Graphics Suite v. 25.2.1.313 (Alludo, Ottawa, Canada).

Individual ML analyses were conducted for each single-marker alignment. Since no topological conflicts were detected among these datasets, the individual gene alignments were manually merged into four final concatenated multi-locus alignments, which were then used for subsequent phylogenetic analyses. The final multi-locus alignments are available as Suppl. material 1.

### ﻿Biogeography assessment through published environmental sequences

Biogeographic assessment of the studied strains, including the analysis of sample types, biomes, and geographic distribution, followed the methodology of Réblová et al. (2022). The evaluation was based on published environmental ITS sequences available in the GlobalFungi database (Větrovský et al. 2020), v. 5 (released on 16 November 2023), which comprises 84 972 samples from 846 studies and a total of 593 399 355 ITS sequence variants. The ITS1 and ITS2 sequences of the studied strains were extracted using the ITSx extractor within the SEED2 platform (Větrovský et al. 2018). Identification of our species in GlobalFungi was performed through an exact match similarity search, comparing all unique ITS1 and ITS2 haplotypes from our study with environmental sequences matching in both length and nucleotide composition. Extensive metadata, including location, substrate, biome, pH and climatic data such as the mean annual precipitation (MAP) and mean annual temperature (MAT), were obtained for each strain from the GlobalFungi database, and are provided in Suppl. material 2. The distribution maps were produced using the QGIS 3.44.4-Solothurn software (QGIS Development Team 2025).

## ﻿Results

Comparative morphological analyses of the seven strains of *P. parvisporum* showed that only CBS 770.83 conforms to the diagnostic characteristics of this species (Preuss 1852; Costantin 1888) and corresponds well with the detailed descriptions provided by Ellis (1968), Holubová-Jechová (1972), and Matsushima (1975). We considered the epitypification of *P. parvisporum* using the strain CBS 770.83. Holubová-Jechová (1972) examined the authentic collection of *Cordana parvispora* from the Preuss herbarium in Berlin and confirmed that it is in good condition and consistent with the original description by Preuss (1852). According to the best practices for epitypification outlined by Lendemer (2020), an epitype should only be designated when the existing type is “demonstrably ambiguous or cannot be critically identified”, which is not the case here. Additionally, the strain CBS 770.83 originates from a different continent and substrate, and therefore does not meet the requirement of geographic and ecological congruence with the type material. Instead, the strain is best regarded as a reference strain, used for DNA sequencing to facilitate molecular comparison and phylogenetic placement.

The remaining strains represent an assemblage of distinct species united by shared morphological traits that include transversely septate, acropleurogenous conidia, holoblastic-denticulate, sympodially elongating conidiogenous cells, and macronematous, mononematous conidiophores. Integration of morphological observations with molecular data allowed for the accurate identification of these strains into species and their placement within the fungal system.

### ﻿Phylogenetic analyses

**Figure 1.**
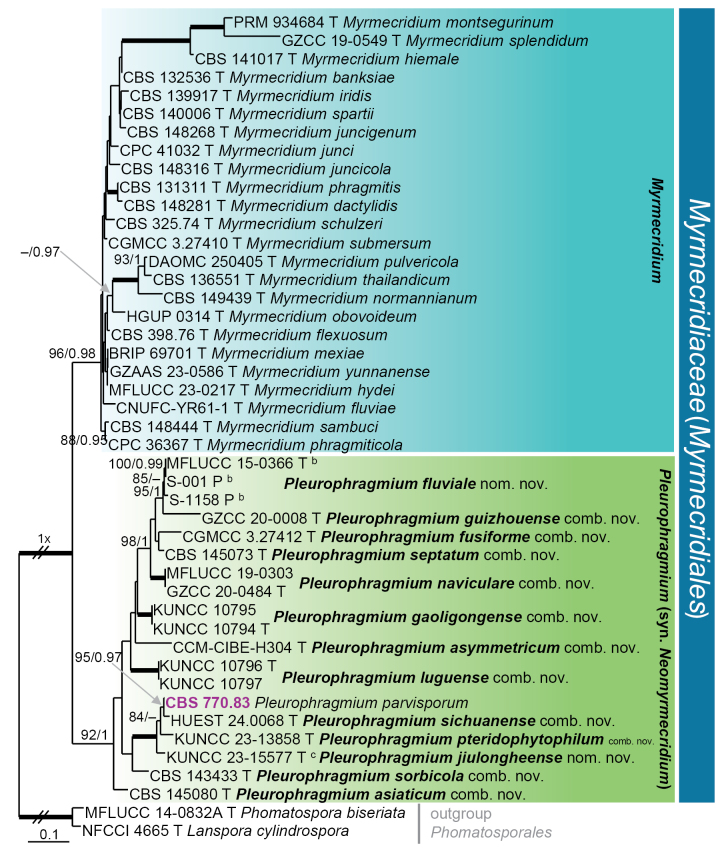
Maximum likelihood phylogenetic tree of the *Myrmecridiales* based on ITS–LSU DNA sequences. Names in bold indicate taxonomic novelties; strain with its accession number in bold and highlighted in violet colour was sequenced in this study; ^b^ = ex-type of *Neomyrmecridium aquaticum*; ^c^ = ex-type of *Neomyrmecridium triseptatum*. T and P denote ex-type and ex-paratype strains. Thickened branches indicate support ML BS = 100% and PP values = 1.0. Branch support of nodes ≥ 70% ML and ≥ 0.95 PP is indicated above or below branches. A hyphen (–) indicates values lower than 75% ML BS or 0.95 PP.

**Figure 2.**
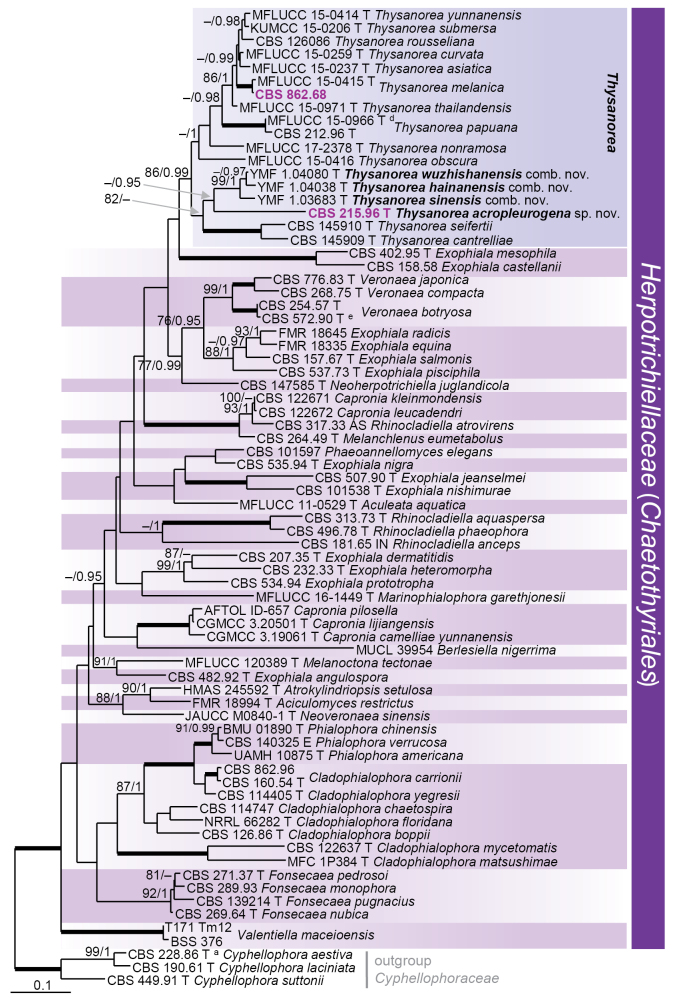
Maximum likelihood phylogenetic tree of the *Herpotrichiellaceae* based on ITS–LSU–*tub2* DNA sequences. Names in bold indicate taxonomic novelties; strains with their accession numbers in bold and highlighted in violet colour were sequenced in this study; ^a^ ex-type of *Cyphellophora vermispora*, ^d^ ex-type of *Thysanorea aquatica*, ^e^ ex-type of *Veronaea constricta*. T, E, IN and AS denote ex-type, ex-epitype, ex-isoneotype, and authentic strains, respectively. Thickened branches indicate support ML BS = 100% and PP values = 1.0. Branch support of nodes ≥ 70% ML and ≥ 0.95 PP is indicated above or below branches. A hyphen (–) indicates values lower than 75% ML BS or 0.95 PP.

**Figure 3.**
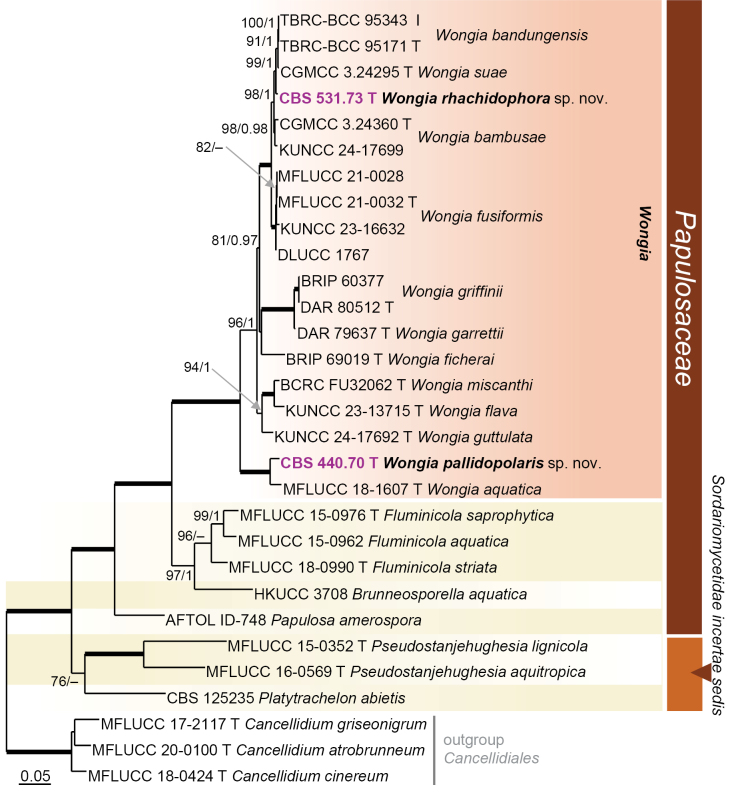
Maximum likelihood phylogenetic tree of the *Papulosaceae* based on ITS–LSU–SSU–*rpb2*–*tef1* DNA sequences. Names in bold indicate taxonomic novelties; strains with their accession numbers in bold and highlighted in violet colour were sequenced in this study. T and I denote ex-type and ex-isotype strains, respectively. Thickened branches indicate support ML BS = 100% and PP values = 1.0. Branch support of nodes ≥ 70% ML and ≥ 0.95 PP is indicated above or below branches. A hyphen (–) indicates values lower than 75% ML BS or 0.95 PP.

**Figure 4.**
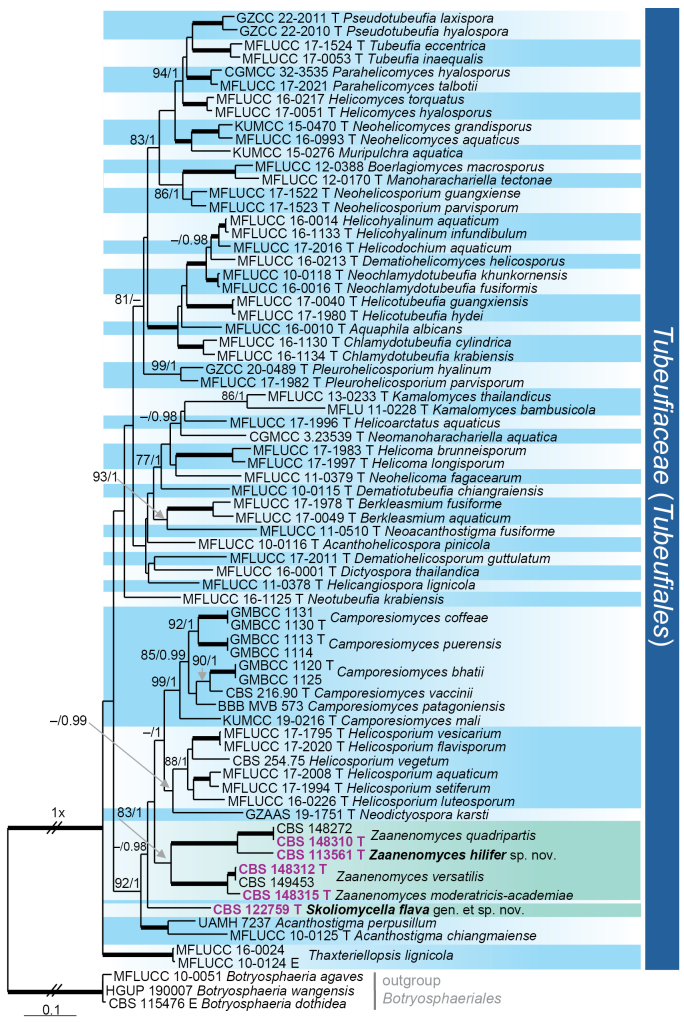
Maximum likelihood phylogenetic tree of the *Tubeufiaceae* based on ITS–LSU–*rpb2*–*tef1* DNA sequences. Names in bold indicate taxonomic novelties; strains with their accession numbers in bold and highlighted in violet colour were sequenced in this study. T and E denote ex-type and ex-epitype strains, respectively. Thickened branches indicate support ML BS = 100% and PP values = 1.0. Branch support of nodes ≥ 70% ML and ≥ 0.95 PP is indicated above or below branches. A hyphen (–) indicates values lower than 75% ML BS or 0.95 PP.

Four independent phylogenetic analyses corroborated our morphological observations and showed that the seven strains originally identified and deposited as *P. parvisporum* in fact represent seven distinct species lineages, distributed across four families and/or orders in three classes. Nodes with ≥ 75% ML bootstrap (BS) support and ≥ 0.95 Bayesian posterior probability (PP) were regarded as well supported. All phylogenetic reconstructions obtained with both ML and BI methods were largely congruent; the ML topologies are illustrated for each dataset (Figs 1–4).

The first phylogenetic analysis (Fig. 1), based on the ITS–LSU dataset, included 43 ingroup strains representing 38 species in two genera of the *Myrmecridiaceae* (*Myrmecridiales*, *Sordariomycetes*). Of the 1 408 characters (including gap positions), 524 were unique sites identified by RAxML: 266 in ITS and 258 in LSU. Two members of the *Phomatosporales*, i.e. *Lanspora cylindrospora* NFCCI 4665 and *Phomatospora biseriata* MFLUCC 14-0832A, were selected as outgroups. The best-fit model of nucleotide substitution selected for both partitions was GTR+I+G. The representative strain of *P. parvisporum* (CBS 770.83) was resolved within the order *Myrmecridiales*. *Myrmecridiales* was split into two strongly supported subclades, corresponding to *Myrmecridium* (96% ML BS/1.0 PP) and *Neomyrmecridium* (92/1.0). The *Neomyrmecridium* lineage includes the ex-type strain of *N. septatum* (CBS 145073), together with ex-type, ex-paratype and other non-type strains of 12 additional species. Within *Neomyrmecridium*, *P. parvisporum* was resolved as sister to *N. sichuanense* (HUEST 240068 ex-type) in a robust subclade (100/1.0) that also encompassed *N. pteridophytophilum* (KUNCC 23-13858 ex-type) and *N. jiulongheense* (KUNCC 23-15577 ex-type). These findings support the reduction of *Neomyrmecridium* to synonymy with *Pleurophragmium*. *Neomyrmecridium aquaticum* (here treated as a new name *P. fluviale*), represented by the ex-type strain MFLUCC 15-0366 together with two paratype strains (S-001, S-1158), and *N. guizhouense* (GZCC 20-0008 ex-type), previously regarded as conspecific by Crous et al. (2021), were resolved as distinct, well-supported lineages.

The second phylogenetic analysis (Fig. 2), conducted on the ITS–LSU–*tub2* matrix, included 71 ingroup strains representing 66 species of the *Herpotrichiellaceae* (*Chaetothyriales*, *Dothideomycetes*). Ninety-nine nucleotides were excluded from the 5′ end of the LSU dataset due to incomplete sequences in most strains. A total of 1 969 characters (including gap positions), 1 003 were unique sites identified by RAxML: 452 in ITS, 239 in LSU, 312 in *tub2*. Three species of *Cyphellophora* (*Cyphellophoraceae*, *Chaetothyriales*), i.e. *C. laciniata*CBS 190.61, *C. suttonii*CBS 449.91, and *C. aestiva*CBS 228.86, were selected as outgroup taxa. The best-fit models of nucleotide substitution selected were: GTR+I+G (ITS), SYM+I+G (LSU), and HKY+I+G (*tub2*). Two strains, CBS 215.96 and CBS 862.68, were resolved within the *Thysanorea* clade (86/99). Strain CBS 215.96 was placed on a terminal branch and represents the novel species *T. acropleurogena*, whereas CBS 862.68 clustered with *T. melanica*, supported by a 99.8% ITS sequence identity. Furthermore, the ex-type strains of three *Uncispora* species, i.e. *U. hainanensis* (YMF1.04038), *U. sinensis* (YMF1.03683), and *U. wuzhishanensis* (YMF1.04080), were nested within the *Thysanorea* clade, corroborating their transfer to *Thysanorea*. These species together formed a sister lineage (71/95) to *T. acropleurogena*. The systematic position of *U. harroldiae*, the type species of *Uncispora*, remains unknown.

The third phylogenetic analysis (Fig. 3), based on the ITS–LSU–SSU–*rpb2*–*tef1* dataset, included 27 ingroup strains representing 21 species in four genera of the *Papulosaceae* and related genera (*Sordariomycetes incertae sedis*). A total of 258 nucleotides were excluded from the 5′ and/or 3′ ends of LSU, SSU, *rpb2*, and *tef1* datasets due to incomplete sequences in most strains. Of the 4 472 characters (including gaps), 1 335 were unique sites identified by RAxML: 328 in ITS, 190 in LSU, 87 in SSU, 481 in *rpb2*, and 249 in *tef1*. Three species of *Cancellidium (Cancellidiales)*, i.e. *C. atrobrunneum* MFLUCC 20-0100, *C. cinereum* MFLUCC 18-0424, and *C. griseo-nigrum* MFLUCC 17-2117, were used as outgroup taxa. The best-fit models of nucleotide substitution selected were: SYM+I+G (ITS), GTR+I+G (LSU, *rpb2*, *tef1*), and GTR+I (SSU). *Papulosaceae* were recovered as a strongly supported lineage (100/1.0) compri­sing *Brunneosporella aquatica*, *Fluminicola* (96/90), *Papulosa amerospora* and *Wongia* (100/1.0), together with the closely related genera *Pseudostanjehughesia* (100/1.0) and *Platytrachelon*. Both strains, CBS 440.70 and CBS 531.73, clustered within the well-supported *Wongia* subclade. The strain CBS 440.70 grouped with *W. aquatica* in a basal position and is here described as a new species, *W. pallidopolaris*. Strain CBS 531.73 was resolved as a new lineage, *W. rhachidophora*, which was nested within a strongly supported subclade (100/1.0). It includes morphologically similar species such as *W. bambusae*, *W. bandungensis*, *W. fusiformis*, and *W. suae* that likely represent a species complex.

To assess the relationships of *W. rhachidophora* with these species, three loci commonly employed for species-level delimitation, ITS, *tef1*, and *rpb2*, were compared. Pairwise sequence identities indicated that *W. rhachidophora* is most closely related to *W. bandungensis* (ITS: 99.4%, *tef1*: 99.0%, *rpb2*: 99.4%) and *W. suae* (ITS: 98.4%, *tef1*: 98.7%, *rpb2*: 99.6%), while showing lower similarity to *W. bambusae* (ITS: 97.1–97.8%, *tef1*: 98.5%, *rpb2*: 98.7%), and *W. fusiformis* (ITS: 95.3%, *tef1*: 98.5%, *rpb2*: 96.9%).

The ITS region shows moderate divergence (0.6–1.6%). The ITS sequences show that *W. rhachidophora* is closest to *W. bandungensis* (99.4%). However, it still shows small differences that may or may not exceed intraspecific variation thresholds. There is also high similarity among *W. rhachidophora* and *W. suae/W. bambusae* (97–98%), but below typical species threshold (≥98.5–99%) (Vu et al. 2019; Lücking et al. 2020). In the *tef1* dataset, all values hover around 98.5–99%, showing strong relatedness across species; *W. rhachidophora* is again most similar to *W. bandungensis*. The *rpb2* sequence of *W. rhachidophora* is shorter 755 bp vs 900–1000 bp in others, which can inflate identity values slightly because less variable regions are often retained. Apart from *W. fusiformis*, all *rpb2* identities are very high (≥98.7%). The highest sequence identity is to *W. suae* and to *W. bandungensis*. Together with morphological distinctiveness (see notes to *W. rhachidophora*), the molecular data support *W. rhachidophora* as a distinct species within the species complex. In addition, the sequence comparison of *W. bandungensis* and *W. suae* indicate that ITS identity (98.6%) is right at the species boundary in fungi. Their *tef1* and *rpb2*, however, show nearly complete identity (≥99.9%), strongly indicating they are the same species.

The final phylogenetic analysis (Fig. 4), conducted on the ITS–LSU–*rpb2*–*tef1* dataset, included 71 ingroup strains representing 65 species of the *Tubeufiaceae* (*Tubeufiales*, *Dothideomycetes*). Ninety-five nucleotides were excluded from the 5′ end of the LSU dataset due to incomplete sequences in most strains. Of the total of 3 512 characters (including gaps), 1 631 were unique sites identified by RAxML: 442 in ITS, 271 in LSU, 538 in *rpb2*, and 380 in *tef1*. Three members of *Botryosphaeria (Botryosphaeriales)*, i.e. *B. agaves* MFLUCC 10-0051, *B. wangensis* HGUP190007, and *B. dothidea*CBS 115476, were selected as outgroup taxa. The best-fit models of nucleotide substitution selected were: GTR+I+G (ITS, LSU, and *tef1*) and SYM+I+G (*rpb2*). Strains CBS 113561 and CBS 122759 were resolved as members of the *Tubeufiaceae*. The resulting topology recovered 37 well-supported generic lineages within the family. Strain CBS 113561 clustered within *Zaanenomyces* clade (83/1.0) closely related to *Z. quadripartis*, supporting its recognition as a new species, *Z. hilifer*. They formed a strongly supported sister lineage (100/1.0) to a subclade (100/1.0) comprising *Z. moderatricis-academiae* and *Z. versatilis*. In contrast, strain CBS 122759 formed a distinct lineage, justifying the establishment of a new monotypic genus, *Skoliomycella*, with *S. flava* as its type species. Both *Skoliomycella* and *Zaanenomyces* were placed within a robust subclade (92/1.0) together with *Acanthostigma* (100/1.0), *Camporesiomyces* (99/1.0), *Helicosporium* (88/1.0), and *Neodictyospora karstii*.

### ﻿Morphological studies

Except for CBS 113561 (see below) and CBS 862.68 (which remained sterile), all other strains could be readily distinguished from *P. parvisporum* under culture conditions. Two strains represent two distinct species of *Thysanorea* (Arzanlou et al. 2007) in the *Herpotrichiellaceae*. *Thysanorea acropleurogena* (CBS 215.96) sporulated abundantly, producing simple or apically loosely branched conidiophores with terminal and intercalary conidiogenous cells forming an elongated denticulate rachis, and pale brown to pale olivaceous brown, predominantly 3-septate conidia. In contrast, *T. melanica* (CBS 862.68) (Liu et al. 2015) remained sterile on all tested media, and its identification relied exclusively on molecular data.

Two other strains belong to the genus *Wongia* in the *Papulosaceae*, where they represent distinct species lineages, here described as *W. pallidopolaris* (CBS 440.70) and *W. rhachidophora* (CBS 531.73). Both species produced brown to dark brown conidia, borne on terminal conidiogenous cells with one to several denticles on relatively short, robust conidiophores.

The two last strains, CBS 113561 and CBS 122759, are members of the *Tubeufiaceae*. Strain CBS 113561 produced hyaline, mostly 3-septate conidia, and simple conidiophores with a short denticulate rachis and sometimes bearing nodulose swellings. In culture, its morphology closely resembled that of *P. parvisporum*, making its distinction challenging. This strain is described here as the new species *Zaanenomyces hilifer*. In contrast, strain CBS 122759 is readily distinguished from *P. parvisporum* by flexuous to sinuous conidiophores with a pronounced zig-zag pattern, bearing solitary denticles scattered along the entire conidiophore axis, and by its 3–7-septate, subhyaline to pale olivaceous-brown conidia. It represents a novel lineage in the *Tubeufiaceae* and is introduced here as the new genus and species *Skoliomycella flava*.

### ﻿Biogeography assessment

**Figure 5.**
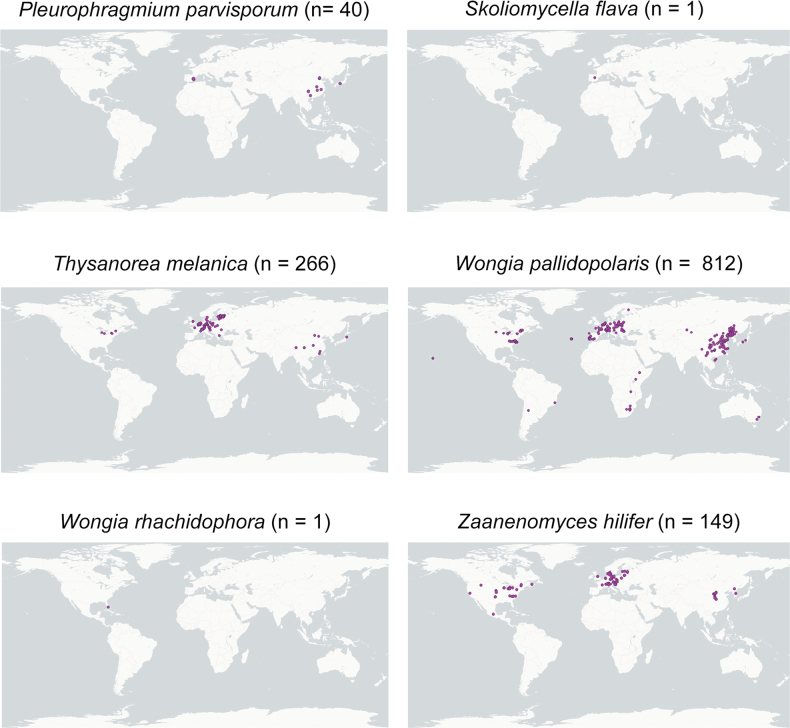
Geographical distribution of the studied species based on environmental DNA records from the GlobalFungi database. Each dot indicates a georeferenced sample in which the species was detected, with the number of samples (n) given in parentheses.

The biogeographic assessment revealed striking contrasts in distribution patterns among the studied taxa (Fig. 5). Some species, such as *T. melanica*, *W. pallidopolaris*, and *Z. hilifer*, are clearly cosmopolitan and ecologically versatile, occurring across multiple continents, biomes, and climate zones. Their strong representation in soils, particularly croplands and grasslands, points to an ecological preference for terrestrial substrates and suggests that human activities, especially agriculture, may have played a role in their global dissemination. The high abundance of *W. pallidopolaris* and *T. melanica* in European croplands is particularly notable and supports the idea that these taxa thrive in disturbed and anthropogenic habitats.

By contrast, other species appear much more restricted. *Pleurophragmium parvisporum* shows a concentration in East Asia with scattered European records, mainly from aquatic habitats, reflecting a narrower ecological niche. These findings provide a broader perspective and contrast with previously published studies, which have so far been based largely on European collections of this species (Costantin 1888; Ellis 1968; Holubová-Jechová 1972) and a few records from Japan (Matsushima 1975) and North America (Wang 2010). *Skoliomycella flava* is represented by a single environmental record in urban air from the Iberian Peninsula, while *W. rhachidophora* shows a disjunct distribution between India and subtropical North America, hinting at either true rarity or under-sampling.

Finally, *T. acropleurogena* remains known only from its type locality, with no matches in environmental sequencing datasets. These patterns collectively illustrate how some lineages are widespread yet cryptic, while others are genuinely rare or overlooked, underscoring the importance of integrating eDNA surveys with traditional taxonomy to resolve their ecology and biogeography.

### ﻿Taxonomy

#### ﻿ Pleurophragmium

Taxon classificationAnimaliaSordarialesLasiosphaeriaceae

Costantin, Les mucédinées simples: 100. 1888.

442DCF9F-B8A6-5B36-8BFA-584E11896568

##### Synonyms.

Dactylaria sect. Pleurophragmium (Costantin) de Hoog, Stud. Mycol. 26: 36. 1985.

*Neomyrmecridium* Crous, Persoonia 41: 287. 2018.

##### Type species.

*Pleurophragmium parvisporum* (Preuss) Hol.-Jech.

##### Description.

**Sexual morph.** Not observed. **Asexual morph. *Colonies*** effuse, hairy, brown to black, subhyaline, beige to pale brown when sporulating; vegetative hyphae immersed or semi-immersed. ***Conidiophores*** macronematous, mononematous, solitary or in small groups, erect, straight or slightly flexuous, unbranched or rarely branched, sometimes elongating percurrently, brown, septate. ***Conidio­genous cells*** integrated, terminal and/or intercalary resulting from the formation of a septum within the original cell during sympodial elongation, polyblastic, occasionally monoblastic, sympodially proliferating, bearing one to several denticles or forming a rachis with minute, pimple-like denticles or protrusions scattered over the surface; conidiogenesis holoblastic-denticulate. ***Conidia*** solitary, dry, acropleurogenous, obovoid or subclavate, fusiform, ellipsoidal, usually tapering towards the base, with a distinct hilum, hyaline, subhyaline or pale brown, sometimes with paler end cells, aseptate or transversely septate, wall smooth or finely ornamented, with an ephemeral mucoid sheath; conidial secession schizolytic.

##### Notes.

Morphological comparisons together with phylogenetic analyses of *P. parvisporum*CBS 770.83 support the view that genera *Neomyrmecridium* and *Pleurophragmium* are congeneric, leading to the proposal of 11 new combinations and the introduction of two new names.

Given the substantial morphological variability among species currently placed in *Pleurophragmium*, the genus is unlikely to represent a single evolutionary lineage, rendering the traditional generic concept untenable. Accordingly, the present generic description is based on the morphological variability of species whose placement is supported by molecular data and refers to *Pleurophragmium* s. str. The following list of species is organised into three categories: (i) species accepted in *Pleurophragmium* s. str.; (ii) species of uncertain status that remain in *Pleurophragmium* s. lat.; and (iii) species excluded from *Pleurophragmium* and transferred to other genera. Names in bold indicate the currently accepted classification, accompanied by full synonymy and, where appropriate, brief explanatory notes. The interspecific variability of species listed in the first two categories is summarised in the synopsis table (Table 3). Key to species of *Pleurophragmium* was provided by D’Souza and Bhat (2012).

**Table 3.** Synopsis table of species of *Pleurophragmium*.

Species accepted in *Pleurophragmium* s. str.											
Taxon	Substrate*	Size (µm)	Conidia				End cells	Ornamentation	Conidiogenous cellsConidiophores		Reference
			Septa	Shape	Sheath	Colour			Size (µm)	Size (µm)	
*Pleurophragmium asiaticum*	C	(13–)15–16(–17) × (3.5–)4.5(–5)	(2–)3	ellipsoid to obovoid, with truncate hilum at the base	present	pale brown		smooth-walled	5–35 × 4–7	50–100 × 3–5	Crous et al. 2018a
*Pleurophragmium asymmetricum*	NS	12–15 × 2–3	1	narrowly clavate or subclavate	absent	yellowish to subhyaline		smooth-walled	8–35 × 3.5–5	40–210 × 3.5–9	Serrano et al. 2020
*Pleurophragmium fluviale*	NS	14–16 × 4–6	3	obovoid, rounded at the apex, pointed at the base	absent	subhyaline		smooth-walled	n/a	211–308 × 5–7	Luo et al. 2019
*Pleurophragmium fusiforme*	NS	29–34 × 4–6	(0–)1–3	navicular to tapering, pointed at both ends	absent	pale brown	paler	smooth-walled	n/a	214–285 × 4–5	Zhang et al. 2024
*Pleurophragmium gaoligongense*	NS	16–24 × 5–7	0–3	clavate-cymbiform, truncate at the base	absent	subhyaline to pale brown		smooth-walled	34–68 × 3–6	138–226 × 4–7	Xu et al. 2023
*Pleurophragmium guizhouense*	NS	8.9–12.7 × 2.8–4.8	(2–)3	fusoid-ellipsoid, apex obtuse or tapering, with a subtruncate hilum at base	absent	subhyaline to pale brown		smooth-walled	2.2–4.3 (width)	75–140 × 2–4.5	Hyde et al. 2020
*Pleurophragmium jiulongheense*	NS	16–23 × 3.7–5.6	3	clavate to fusiform, truncate at the base	present	subhyaline to pale brown		smooth-walled	n/a	(144–)204–332 × 3.4–4.8	Cao et al. 2025
*Pleurophragmium luguense*	NS	12–15 × 4–7	(0–)2–3	obovoid, tapering at the base	absent	subhyaline to pale brown		smooth-walled	39–68 × 3–5	152–298 × 4–6	Xu et al. 2023
*Pleurophragmium naviculare*	NS	16–24 × 5.5–7.5	(1–) 3	navicular to fusiform, tapering to a hilum towards the base, obtuse at the apex	present	hyaline, becoming pale brown	paler	smooth-walled	n/a	100–200 × 4–5.6	Yang et al. 2023
*Pleurophragmium parvisporum*	NS	10–18 × 3.5–6	(0–)3(–4)	ellipsoid to subclavate, rounded at the apex, pointed at the base	absent	hyaline to very pale brown		crumpled	26–47(–51) × 4.5–5.5	(80–)118–250(–270) × (3.5–)4–5	This study
*Pleurophragmium pteridophytophilum*	NS	8.5–11 × 3–4	0–1	obovoid	absent	hyaline to pale brown		rough-walled	20–55.5 × 2.5–4(–4.5)	150–224 × 2.7–4.9	Zhang et al. 2025
*Pleurophragmium septatum*	C	(12–)14–16(–20) × (3.5–)4(–5)	(1–) 3	fusoid-ellipsoid, apex obtuse, tapering in lower third to a truncate hilum	present	hyaline, becoming pale brown		smooth-walled	30–40 × 4–5	40–70 × 4–5	Crous et al. 2018a
*Pleurophragmium sichuanense*	NS	9.5–14.5 × 4–4.5	0–1	fusiform or narrowly obovoid	absent	subhyaline to pale brown		finely verrucose	n/a	93–130 × 4–5	Chen et al. 2025
*Pleurophragmium sorbicola*	C	(7–)8–10(–15) × 4(–5)	(0–)1(–3)	obovoid, obtuse at the apex	present	hyaline, pale brown with age		smooth-walled	20–65 × 3–4	50–200 × 4–7	Crous et al. 2018b
***Pleurophragmium* s. lat.: species of uncertain status**											
Pleurophragmium angamosense	C	24.5–40 × 5–8	3–7 mostly 5	fusiform, curved or straight	absent	pale brown	paler	smooth-walled	3–3.5 (width)	200 × 3.5–4.5	Matsushima 1995
Pleurophragmium aquaticum	NS	25–30 × 6–6.5	3	fusiform to clavate, sometimes navicular, truncated at the base	absent	brown, paler around septa	paler	smooth-walled	n/a	200–390 × 5–11	Heredia et al. 2007
Pleurophragmium bitunicatum	C	20–35 × 4.5–7	3(–5)	fusiform, narrowly truncated at the base	absent	brown		smooth-walled	n/a	(50–) 100–220 × 3–5	Matsushima 1975
Pleurophragmium clavatum	NS	9.5–16.5 × 3.5–4.5	0	clavate to fusiform, narrower at the ends, tapered to a point at the base	absent	yellowish brown		verrucose	n/a	75–160 × 3.3–4	Ma et al. 2014
Pleurophragmium ellipsoideum	NS	10–17 × 6–8.5	3	ellipsoid to obovoid, rounded at the apex, pointed at the base	absent	pale brown		smooth-walled	n/a	150–260 × 3.5–6	Ma et al. 2014
Pleurophragmium harunganae	NS	12–18 × 5–7	0	fusoid, curved	absent	subhyaline to pale olivaceous		smooth-walled	n/a	150 × 4–5	Hansford 1946
Pleurophragmium indicum	NS	20–30 × 4.5–11	3	ellipsoidal to obovoid, rounded at the apex, pointed at the base, straight to slightly curved	absent	dark brown	paler	smooth-walled	15–23 × 4.5–9.5	100–160 × 7–22	D‘Souza and Bhat 2012
Pleurophragmium malaysianum	C	(20–) 40–75 × 4–5	3–10 pseudoseptate	cylindrical to clavate, rounded at the apex, protruding at the base	absent	hyaline		smooth-walled	n/a	25–50 (–100) × 3.5–5	Matsushima 1996
Pleurophragmium miniumbonatum	NS	16–19 × 6–7	(2–) 3	obovoid, pyriform to broadly clavate, sometimes slightly clavate, umbonate at the apex, truncate at the base	absent	dark brown to brown	subhyaline basal cell	smooth-walled	55–95 × 3–3.5	90–150 × 5.5–7	Castañeda 1999
Pleurophragmium naviculiforme	C	18–32 × 6.5–10	1	navicular, pointy at the apex, pointed at the base	absent	hyaline		smooth-walled	n/a	70–140 × 6–8	Matsushima 1975
Pleurophragmium obcampanuloides	C	(10–)11.5–15.5(–18) × 5–6.5(–7)	(1–)2	obpyriform to turbinate	absent	pale olivaceous, olivaceous in mass		smooth-walled	n/a	100–150 × 3.8–5	Matsushima 1995
Pleurophragmium peruamazonicum var. peruamazonicum	C	10–30 × 4–6	2	cylindrical, rounded at the apex, pointed at the base	absent	pale brown, fuscous in mass	subhyaline	smooth-walled	n/a	100–300 × 3–4	Matsushima 1993
Pleurophragmium peruamazonicum var. inflatum	C	13–20 × (5–)6–9(–10)	(1–)2	ovoid, rounded at the apex, narrow at the base	absent	pale sooty	subhyaline apical cell	smooth-walled	n/a	(50–) 200–600 × 4–5.5	Matsushima 1993
Pleurophragmium subfusiforme	C	21–38 × 5.8–9.2	3–7	fusiform, straight to sometimes curved	absent	subhyaline, pale fuscous in mass		smooth-walled	n/a	200–500 × 4–5	Matsushima 1975
Pleurophragmium taiwanense	C	12–20 × 3–4.5	3	cylindro-clavate, with a slightly inflated apical cell	absent	pale brown	hyaline	smooth-walled	n/a	70–200 (–250) × 3.5–4.5	Matsushima 1987
Pleurophragmium tricolor	NS	17–18 × 4–5	2	ellipsoidal with, rounded at the apex, pointed at the base	absent	brown, light brown basal cell	hyaline apical cell	smooth-walled	n/a	196–200 × 5–7	Rambelli 2009
Pleurophragmium varieseptatum	NS	19–22 × 4–5	1–4	cylindrical, rounded at the apex, pointed at the base	absent	pale olivaceous, olivaceous-grey in mass		smooth	n/a	70–125 × 3.5–5	Matsushima 1975
Pleurophragmium verruculosum	C	10–16.5 × 3.3–5	1–3(–3)	subclavate, clavate, pointed at the base	absent	subhyaline to pale brown		verrucose	n/a	8.2–26.4 (–15) × 1.6–4.5 (–3)	Tiwari et al. 1969
Pleurophragmium yunnanense	NS	10–15 × 6–7	(2–)3	broadly fusiform, narrowed at the ends, tapered to a point at the base	absent	pale brown		smooth	n/a	185–270 × 3.5–4.5	Ma et al. 2014

* natural substrate (NS); culture (C).

*Aquapteridospora* (*Distoseptisporales*, *Sordariomycetes*) (Yang et al. 2015), is a dematiaceous hyphomycete genus that warrants comparison with *Pleurophragmium*. Both genera share rigid, erect, darkly pigmented, simple conidiophores on the natural substrate that bear terminal and frequently intercalary holoblastic-denticulate conidiogenous cells that proliferate sympodially, as well as septate conidia that may be surrounded by a mucoid sheath. However, they differ primarily in conidial pigmentation. In *Pleurophragmium* s. str., conidia are typically hyaline to subhyaline, becoming pale brown at maturity or when in mass, occasionally showing paler end cells, seldom with a mucoid sheath, and have thin-walled septa. *Pleurophragmium fusiforme* is an exception in having pale brown conidia with paler ends. However, in *Aquapteridospora* conidia are primarily pigmented, continuously brown or dark brown, often with paler end cells, with thick-walled septa and are often embedded in a mucoid sheath. The exception is *A. hyalina* described with hyaline conidia that become subhyaline to pale brown at maturity (Ma et al. 2022). Based on molecular evidence, *Pleurophragmium bambusinum* (Dai et al. 2016) has been transferred to *Aquapteridospora* by Bao et al. (2021). In addition, several other *Pleurophragmium* species of uncertain status (see below) and which lack molecular data, show close morphological similarity with members of this genus.

*Pleurotheciella* (Réblová et al. 2012) is another genus warranting comparison with *Pleurophragmium*. Despite their close morphological resemblance in asexual characteristics, which makes them difficult to distinguish, the two genera are clearly separated phylogenetically. *Pleurotheciella* belongs to the *Pleurotheciales*, where it forms a robust and species-rich lineage.

###### ﻿Species accepted in *Pleurophragmium* s. str.

Here we list 14 species currently accepted in *Pleurophragmium* s. str. whose placement is supported by molecular data. These species are morphologically very similar, with conidial characters providing the primary basis for distinction. Several species of *Pleurophragmium* are known only from culture, with no confirmed wild type (the typical form of a species as it occurs in nature). This reliance on *in vitro* observations may complicate identification and lead to distortion of key traits, i.e. distribution of fertile regions on the conidiophore (apically or in nodulose swellings), conidiophore appearance, conidial dimensions and septation patterns, and even the presence of a mucoid sheath. In culture, the morphology of conidiophores and conidia may deviate from that observed in the wild type (Réblová et al. 2021, 2022). In addition, the conidial size of species currently referred to *Pleurophragmium* s. str. show considerable overlap, making it difficult to distinguish species based solely on conidial dimensions.

#### ﻿ Pleurophragmium asiaticum

Taxon classificationAnimaliaSordarialesLasiosphaeriaceae

(Crous) Réblová & Hern.-Restr. comb. nov.

0E28977F-1EBA-588C-87E6-B67258B945D1

860799

##### Basionym.

*Neomyrmecridium asiaticum* Crous, Persoonia 41: 291. 2018.

##### Typus.

THAILAND • Ratchaburi Province; on leaves of unidentified vine; 2008; P. W. Crous HPC 2252 (holotype CBS H-23774, culture ex-type CPC 34535 = CBS 145080).

##### Notes.

*Pleurophragmium asiaticum* resembles *P. septatum* in the characters of its conidiophores, conidiogenous cells, and subhyaline to pale brown, mostly 3-septate conidia. However, both species represent separate evolutionary lineages. It can also be compared to *P. taiwanense* (Matsushima 1987) which differs by producing cylindro-clavate conidia with a slightly inflated apical cell and significantly longer conidiophores. Both species were described from cultures, and their protologues are therefore directly comparable.

#### ﻿ Pleurophragmium asymmetricum

Taxon classificationAnimaliaSordarialesLasiosphaeriaceae

(R.F. Castañeda, Serrano & D. Sosa) Réblová & Hern.-Restr. comb. nov.

900D72A7-77CE-57BB-8DF4-80A0DEE5BF0C

860800

##### Basionym.

*Neomyrmecridium asymmetricum* R.F. Castañeda, Serrano & D. Sosa, Mycotaxon 135: 157. 2020.

##### Typus.

ECUADOR • Guayas Province, Guayaquil, Balao; 02°48.00'S, 079°40.00'W; on decaying leaves of *Theobroma cacao*; 8 Jul 2017; F. Espinoza & S. Pérez-Martínez (holotype URM 90896, ex-type culture CCMCIBE-H304 = CCMCIBE-H304-A).

##### Notes.

The species is readily distinguished by its 1-septate conidia, which are yellowish to subhyaline and vary from narrowly clavate or subclavate to almost narrowly triangular.

#### ﻿ Pleurophragmium fluviale

Taxon classificationAnimaliaSordarialesLasiosphaeriaceae

Réblová & Hern.-Restr. nom. nov.

ABC6B043-BFEA-50F3-B66B-34CB35878445

860801

##### Basionym.

*Myrmecridium aquaticum* Z.L. Luo, K.D. Hyde & H.Y. Su, Fungal Diversity 99: 501. 2019.

##### Synonym.

*Neomyrmecridium aquaticum* (Z.L. Luo, K.D. Hyde & H.Y. Su) Crous, Persoonia 47: 201. 2021.

##### Etymology.

From Latin *fluviale* (“of a river” or “riverine”). Refers to the aquatic habitat from which the species was originally collected.

##### Typus.

CHINA • Yunnan Province, Lancang River; saprobic on submerged decaying wood; Apr 2015; X. C. Tao S-448 (holotype MFLU 18-1595, isotype HKAS 92833, ex-type culture MFLUCC 15-0366 = KUMCC 15-0340).

##### Notes.

The protologue of *P. fluviale* (as *M. aquaticum*, Luo et al. 2019) included the ex-type strain MFLUCC 15-0366 and two paratype strains, MFLUCC 18-1489 = S-1158, and S-001. In the original phylogenetic analysis, all three strains were resolved as conspecific. *Myrmecridium aquaticum* was later transferred to *Neomyrmecridium* by Crous et al. (2018a), who treated *P. guizhouense* (as *Neomyrmecridium guizhouense*, Hyde et al. 2020) as its synonym, based on a phylogenetic analysis that included the ex-type strain of *P. guizhouense* (GZCC 20-0008) and both paratype strains of *P. fluviale*. However, the ex-type strain of *P. fluviale* was not included in that analysis. In the present phylogenetic analysis (Fig. 1), which includes sequences from available ex-type and paratype strains of both species, *P. fluviale* and *P. guizhouense* are shown to represent distinct species.

The name *P. fluviale* is based on *M. aquaticum*, but the epithet ‘*aquaticum*’ is unavailable in this genus due to the prior valid publication of *P. aquaticum* (Heredia et al. 2007). Therefore, a new name is proposed for *M. aquaticum*.

Among *Pleurophragmium* species, *P. fluviale* shows some similarity to *P. parvisporum* in conidial morphology. However, it clearly differs in smooth-walled conidia that remain subhyaline at maturity, and longer conidiophores.

#### ﻿ Pleurophragmium fusiforme

Taxon classificationAnimaliaSordarialesLasiosphaeriaceae

(Liang Zhang bis & Z.L. Luo) Réblová & Hern.-Restr. comb. nov.

D1C593CE-901A-55DE-9115-EA9D680F45D4

860802

##### Basionym.

*Neomyrmecridium fusiforme* Liang Zhang bis & Z.L. Luo, J. Fungi 10: 19. 2024.

##### Typus.

CHINA • Yunnan Province, Yuanjiang River basin; 23°48.20'N, 101°47.35'E; on submerged decaying wood in a freshwater stream; 22 Feb 2022; H. W. Shen S-3712 (holotype KUN-HKAS 132122, ex-type culture CGMCC 3.27412 = KUNCC 23-17153).

##### Notes.

The species resembles *P. naviculare* (Yang et al. 2023) in having navi­cular to fusiform, pale brown conidia with paler end cells, but it is distinguished by conidia that are longer, narrower, smooth-walled, and lacking a mucoid sheath.

#### ﻿ Pleurophragmium gaoligongense

Taxon classificationAnimaliaSordarialesLasiosphaeriaceae

(R.J. Xu, Q. Zhao & Boonmee) Réblová & Hern.-Restr. comb. nov.

1BF44D8F-DC14-5349-A2EC-EAD4BC60A7A2

860803

##### Basionym.

*Neomyrmecridium gaoligongense* R.J. Xu, Q. Zhao & Boonmee, Curr. Res. Environm. Appl. Mycol. 13: 492. 2023.

##### Typus.

CHINA • Yunnan Province, Lushui City, Pian Ma, Gaoligong Mountains; 25°58.15'N, 98°41.02'E; on submerged decaying wood in freshwater habitats; 3 050 m a.s.l.; 29 Apr 2021; R. J. Xu GLG-07 (holotype HKAS 124621, ex-type culture KUNCC 10794).

#### ﻿ Pleurophragmium guizhouense

Taxon classificationAnimaliaSordarialesLasiosphaeriaceae

(N.G. Liu, K.D. Hyde & J.K. Liu) Réblová & Hern.-Restr. comb. nov.

5EFF8381-14CF-5737-BFF6-967D0E92697E

860804

##### Basionym.

*Neomyrmecridium guizhouense* N.G. Liu, K.D. Hyde & J.K. Liu, Fungal Diversity 100: 187. 2020.

##### Typus.

CHINA • Guizhou Province, Dushan; on decaying wood in freshwater; 6 Jul 2018; N. G. Liu DS002 (holotype GZAAS 20-0001, ex-type culture GZCC 20-0008).

#### ﻿ Pleurophragmium jiulongheense

Taxon classificationAnimaliaSordarialesLasiosphaeriaceae

Réblová & Hern.-Restr. nom. nov.

4BF0C820-7272-5DEF-9579-E0BA20409DDA

860805

##### Basionym.

*Neomyrmecridium triseptatum* W.P. Wang & Z.L. Luo, Fungal Diversity 132: 499. 2025.

##### Etymology.

From Jiulonghe River, the geographic locality where the type specimen was collected.

##### Typus.

CHINA • Yunnan Province, Qujing City, Luoping County, Jiulonghe River; 25°32.86'N, 104°29.31'E; on submerged decaying stem of unknow species of *Bambusoideae*; 15 Jul 2023; W. P. Wang S-5133 & S-5134 (holotype HKAS 136944, ex-type culture KUNCC 23-15577).

##### Notes.

The name *P. jiulongheense* is based on *N. triseptatum* (Cao et al. 2025), but the epithet ‘*triseptatum*’ is unavailable in this genus due to prior valid publication of *P. triseptatum* (Matsushima 1975; = *Dactylaria triseptata* (Castañeda and Kendrick 1991)). Therefore, a new name is proposed for *N. triseptatum*.

#### ﻿ Pleurophragmium luguense

Taxon classificationAnimaliaSordarialesLasiosphaeriaceae

(R.J. Xu, Q. Zhao & Boonmee) Réblová & Hern.-Restr. comb. nov.

5C17EA22-1CB4-5350-8169-EB0B9F6D5CBC

860806

##### Basionym.

*Neomyrmecridium luguense* R.J. Xu, Q. Zhao & Boonmee, Curr. Res. Environm. Appl. Mycol. 13: 495. 2023.

##### Typus.

CHINA • Yunnan Province, Ninglang County, Lugu Lake; 27°40.07'N, 100°47.08'E; saprobic on submerged decaying wood in freshwater habitats; 2 672 m a.s.l.; 5 Mar 2021; H. W. Shen L1127, (holotype HKAS 124601, ex-type culture KUNCC 10796).

##### Notes.

*Pleurophragmium lugulense* resembles *P. obcampanuloides* (Matsushima 1995); although their 2–3-septate conidia are similar in size, they differ in shape, being obovoid and tapering to a point in *P. lugulense*, compared to obpyriform and more turbinate in the latter species. It can also be compared to *P. yunnanense* (Ma et al. 2014); however, *P. yunnanense* differs in broadly fusiform conidia.

#### ﻿ Pleurophragmium naviculare

Taxon classificationAnimaliaSordarialesLasiosphaeriaceae

(Jing Yang, Jian K. Liu & K.D. Hyde) Réblová & Hern.-Restr. comb. nov.

6ABE52B4-46FA-5308-B534-7DF4A540B3DA

860807

##### Basionym.

*Neomyrmecridium naviculare* Jing Yang, Jian K. Liu & K.D. Hyde, Fungal Diversity 119: 161. 2023.

##### Typus.

CHINA • Guizhou Province, Anshun City, Gaodang Village; 26°04.26'N, 105°41.88'E; on decaying wood submerged in Suoluo River; 17 Oct 2018; J. Yang GDT38-1 (holotype HKAS 124639, ex-type culture GZCC 20-0484).

#### ﻿ Pleurophragmium parvisporum

Taxon classificationAnimaliaSordarialesLasiosphaeriaceae

(Preuss) Hol.-Jech., Česká Mykol. 26: 223. 1972.

227E2970-6A3C-55AF-BD59-73A3291720D2

Figs 6, 7, 8

##### Basionym.

*Cordana parvispora* Preuss, Linnaea 25: 728. 1852.

##### Synonyms.

*Acrothecium simplex* Berk. & Broome, Ann. Mag. Nat. Hist., Ser. 3 7: 382. 1861.

Acrothecium simplex var. elatum Grove, J. Bot., 24: 203. 1886; Sacc., Syll. fung. 4: 486. 1886.

*Acrothecium parvisporum* (Preuss) Sacc., Syll. fung. 4: 485. 1886.

**Figure 6.**
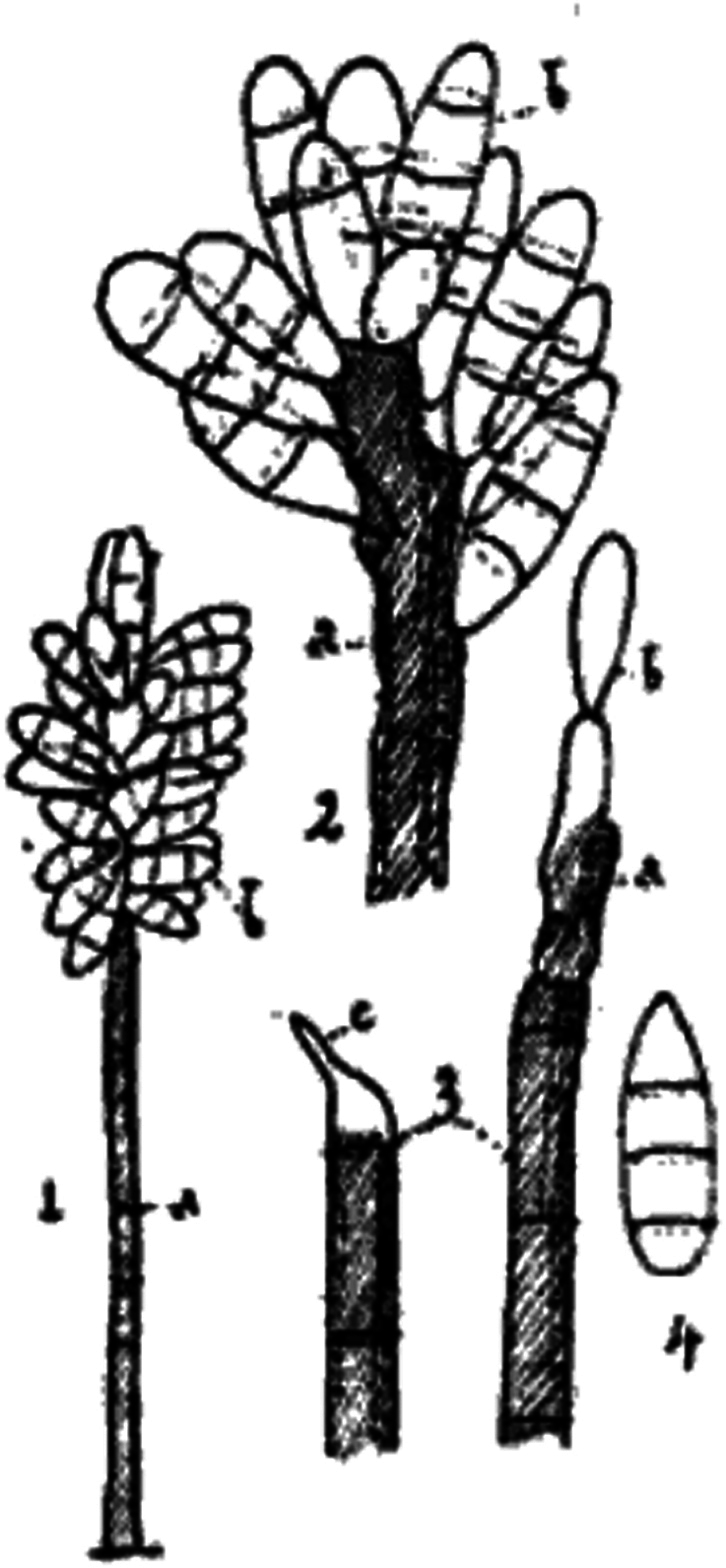
Illustration of conidiophores, conidiogenous cells and conidia of *Pleurophragmium parvisporum* (as *P. bicolor*, Costantin 1888).

*Pleurophragmium bicolor* Costantin, Mucéd. Simpl.: 100. 1888.

*Acrothecium bicolor* (Costantin) Sacc., Syll. fung. 19: 14. 1910.

*Pleurophragmium simplex* (Berk. & Broome) S. Hughes, Canad. J. Bot. 36: 798. 1958.

*Dactylaria parvispora* (Preuss) de Hoog & Arx, Kavaka 1: 58. 1974 [1973].

Synonymy adopted from Hughes (1958) and Holubová-Jechová (1972).

##### Description on the natural substrate.

**Sexual morph.** Not observed. **Asexual morph. *Colonies*** effuse, hairy, dark brown to black, beige to pale brown when sporulating; vegetative hyphae immersed or semi-immersed, brown. ***Coni­diophores*** (80–)118–250(–270) × (3.5–)4–5 µm, often inflated at the base up to 8–11 µm wide, macronematous, mononematous, solitary, erect, straight or slightly flexuous, cylindrical, unbranched, elongating percurrently, bearing a visible frill, brown, lower part usually dark brown and thick-walled, upper part pale brown, thinner-walled, smooth-walled, septate. ***Conidiogenous cells*** 26–47(–51) × 4.5–5.5 µm, integrated, terminal, sometimes intercalary resulting from the formation of a septum within the original cell, polyblastic, occasionally monoblastic, proliferating sympodially forming a rachis with numerous minute pimple-like denticles or protrusions scattered over the surface, cylindrical, tapering or slightly inflated in the upper part, pale brown, subhyaline towards the apex, smooth-walled; conidiogenesis holoblastic-denticulate. ***Conidia*** 10–18 × 3.5–6 µm (mean ± SD = 13.8 ± 2.0 × 4.8 ± 0.7 μm), solitary, dry, acropleurogenous, ellipsoidal, obovoid to subclavate, straight, rounded at the apex, tapering towards the base, with a minute basal scar, hyaline to subhyaline, very pale brown in mass, (0–)3(–4)-septate, mucoid sheath is inconspicuous, ephemeral, disintegrates as the conidium matures and remains collapsed on the conidial wall giving the conidia a longitudinally crumpled appearance, ornamentation absent at the apex; conidial secession schizolytic.

##### Culture characteristics.

On CMD colonies 46–48 mm diam., circular, flat, margin entire, mucoid, glossy, pale pink-orange, pale yellow pigment diffusing into the agar, reverse orange. On MLA colonies 44–48 mm diam., circular, flat, margin entire, mucoid, glossy, zonate, apricot at the centre, paler towards the periphery, pale ochre at the margin, yellow pigment diffusing into the agar, reverse yellow-orange. On OA colonies 52–54 mm diam., circular, flat, margin entire, mucoid, glossy, distinctly zonate, whitish- to golden-yellow with deep salmon to orange centre and margin, with an intermediate zone of sparse growth and almost no pigmentation, golden-yellow pigment diffusing into the agar, reverse pale yellow. On PCA colonies 48–50 mm diam., circular, flat, margin entire, mucoid, golden-yellow at the centre, pale yellow towards the periphery, pale yellow pigment diffusing into the agar, reverse pale yellow. Sporulation sparse on OA, absent on CMD, MLA and PCA.

##### Description in culture.

***Colonies*** on OA effuse. **Sexual morph.** Not observed. **Asexual morph. *Mycelium*** composed of hyaline, septate hyphae, 1–2.5 µm wide. Conidiophores, conidiogenous cells and conidia similar to those on the natural substrate. ***Conidiophores*** 88–148 × 3–4 µm in 4 wk, inflated at the base up to 8–9.5 µm wide, extending up to 221 µm long in 8 wk due to the repeated sympodial proliferation of the conidiogenous cells, brown to dark brown and thick-walled in the lower part, paler and thinner-walled towards the apex. ***Co­nidiogenous cells*** 12–27.5(–34) × (2.5–)3–3.5 µm, integrated, terminal, forming transverse septa during sympodial proliferation and often becoming intercalary with clusters of closely spaced minute denticles or protrusions, arranged along most of the conidiophore length, at the apex with a short rachis of minute denticles or protrusions, polyblastic, cylindrical, pale brown to pale olivaceous brown, apex tends to be paler, smooth-walled; conidiogenesis holoblastic-denticulate. ***Conidia*** (9.5–)10–14 × 3–4.5 µm (mean ± SD = 11.6 ± 1.0 × 3.6 ± 0.3 μm), hyaline to subhyaline to pale brown, 3-septate, with a wing-like mucoid sheath located around the middle, or covering the upper two-thirds of the conidium; sheath collapses upon aging, becoming inconspicuous, giving the conidia a longitudinally crumpled appearance, ornamentation absent at the apex. Occasionally the sheath disintegrates towards the upper part of the conidium, where it ruptures and partially detaches from the wall.

##### Specimens examined.

CZECH REPUBLIC • Central Bohemian Region, Týřovické skály National Nature Reserve near Týřovice; on decaying wood of a trunk of *Carpinus betulus*; 7 May 1971; V. Holubová-Jechová 8587 (PRA-24033); Ibid.; Prague, in the valley of Radotínský potok between Choteč and Radotín; on decaying stem of *Smyrnium perfoliatum*; 19 May 1990; V. Holu­bová-Jechová 8553 (PRA-24032); • Hradec Králové Region, Orlické hory Mts., W of Hadinec near Neratov; 830–880 m a.s.l.; on the inner side of decaying bark of a trunk *Fagus sylvatica*; 20 Jul 1969; V. Holubová-Jechová 8588 (PRA- 24034); • Olomouc Region, Hrubý Jeseník Mts., U Kříže forest between Bělá pod Pradědem and Vidly; on decaying wood of *Fagus sylvatica*; 8 Aug 1971; V. Holubová-Jechová 8584 (PRA-24035); • Ibid.; Bučina virgin forest above Františkova myslivna cabin near Kouty nad Desnou; on decaying wood of a branch of *Fagus sylvatica*; 4 Aug 1971; V. Holubová-Jechová 8586 (PRA-24036). JAPAN • Kyoto, Daitokuji Tempel D; on unidentified dead twigs; 28 Aug 1983; W. Gams & M. Tsuda (CBS H-3522, dried culture CBS H-3506, living culture CBS 770.83). SLOVAK REPUBLIC • Lúčanská Malá Fatra Mts., Šrámková National Nature Reserve, Kýčery; on decaying wood of *Fagus sylvatica*; 26 Sep 1983; V. Holubová-Jechová 8556 (PRA-24037).

**Figure 7.**
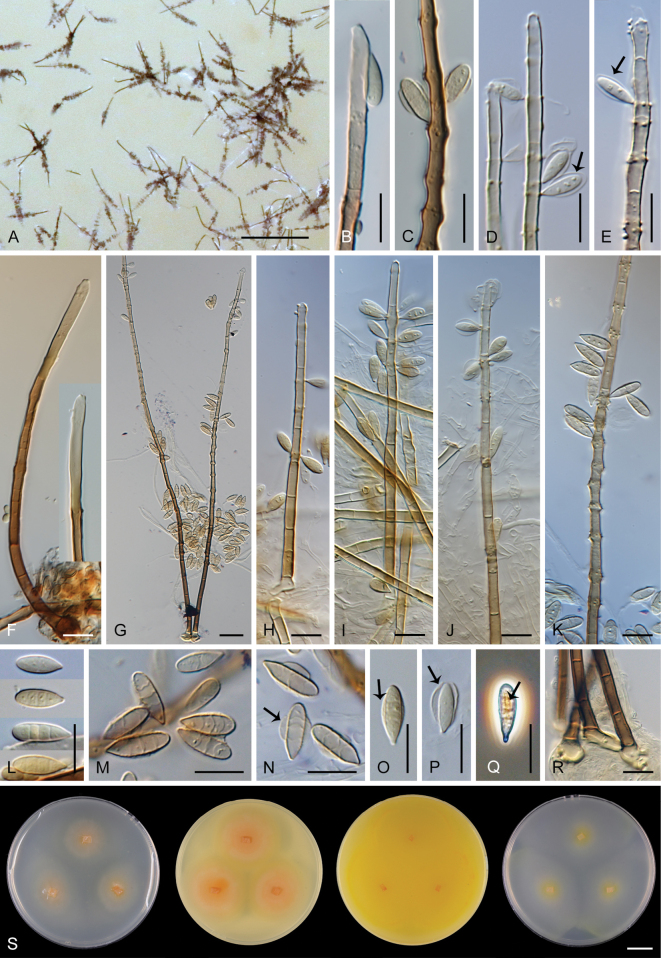
*Pleurophragmium parvisporum*. **A** Sporulating conidiophores **B–E** upper part of conidiophores with terminal and intercalary conidiogenous cells (nodulose swellings) and conidia (arrows indicate mucoid sheath covering the upper two-thirds of the conidium) **F–K** conidiophores with conidiogenous cells and conidia **L–P** conidia (in N, O arrows indicate mucoid wing-like sheath; in P arrow indicates mucoid sheath that disintegrates in the upper part and detaches from the wall **Q** conidium with longitudinally crumpled wall (arrow indicates the original collapsed sheath which causes the ornamentation) **R** basal cells of conidiophores **S** diversity of colony morphology on CMD, MLA, OA, and PCA, respectively (from left to right) after 4 wk. Images: CBS 770.83 on OA (A–E, G–K, M–Q); CBS H-3522 on natural substrate (F, L). Scale bars: 300 µm (**A**); 20 µm (**G, R**); 10 µm (**B–F, H–Q**); 1 cm (**S**).

##### Habitat and geographical distribution.

Saprobe on decaying wood and herbaceous stems of various hosts, such as *Arctium lappa*, *Brassica oleracea*, *Campanula medium*, *Carpinus betulus*, *Conium maculatum*, *Epilobium hirsutum*, *Fagus sylvatica*, *Filipendula ulmaria*, *Heracleum sphondylium*, *Lithocarpus edulis*, *Polygonum sieboldii*, *Quercus* sp., *Sambucus ebulus*, *Urtica dioica* in the Belgium, Czech Republic, Denmark, France, Germany, Japan, Mexico, Netherlands, New Zealand, Slovak Republic, UK and New Jersey and Washington, USA (Preuss 1852; Costantin 1888; Berkeley and Broome 1861; Ellis 1968; Holubová-Jechová 1972; Matsushima 1975; Wang 2010; GBIF Secretariat 2023).

According to GlobalFungi database, *P. parvisporum* was detected in 40 samples. Most records originate from Asia, primarily from Japan and China, with a smaller contribution from South Korea. Additional records come from Europe, all from Spain. The species is most frequently detected in freshwater aquatic habitats (45%), followed by anthropogenic/urban sites (50%), shrubland (17.5%), forest (15%), and cropland (2.5%) biomes. It was detected from water (45%), soil (35%), air (17.5%), and dust (2.5%). These samples were collected across a wide elevational range, 26–948 m, suggesting no strong restriction to high- or low-altitude habitats. Occurrences are associated with MAT ~15.4 °C and MAP ~1 059 mm/year.

##### Notes.

*Pleurophragmium* was originally described with a single species, *P. bicolor*, with the protologue and accompanying illustration (Costantin 1888: fig. 70) reproduced here in Fig. 6. The species was treated in detail under the synonymous names *P. simplex* by Ellis (1968), and *P. parvisporum* by Holubová-Jechová (1972) and Matsushima (1975). These authors reported a relatively broad conidial length range based on multiple collections from wood and herbaceous stems: 10–21 µm (mostly 15.2 µm) × 3.5–6 µm (mostly 4.5 µm) (Ellis 1968), and 12.5–22.5 (mostly 15–18.5 µm) × 3.5–6.5 µm (mostly 5 µm) (Holubová-Jechová 1972); for Matsushima’s measurements, see below. Collections from the Czech Republic and Japan examined in this study are consistent with these accounts, although the maximum conidial length observed was slightly shorter than in the published ranges. Our observations confirm that within a single species, conidial dimensions can be variable and are fully congruent with previous descriptions.

**Figure 8.**
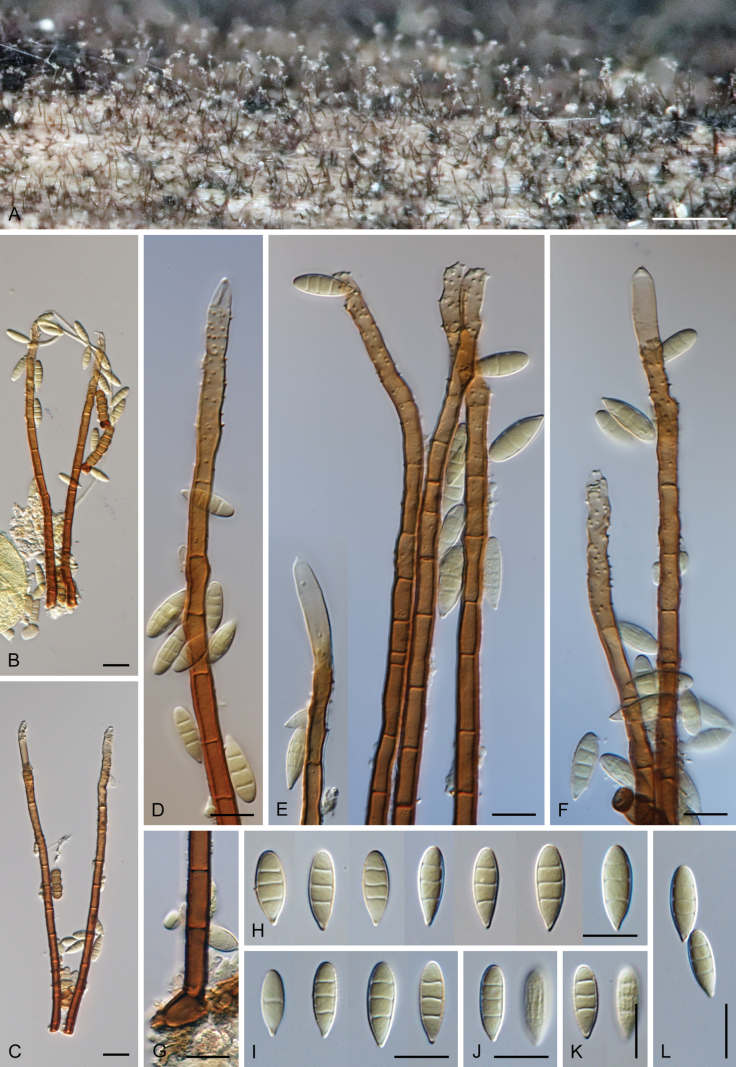
*Pleurophragmium parvisporum* (PRA-24032). **A** Sporulating conidiophores **B–F** conidiophores with conidiogenous cells and conidia **G** Basal cell **H–L** conidia. Images: on natural substrate. Scale bars: 300 µm (**A**); 20 µm (**B, C**); 10 µm (**D–L**).

In the Japanese material used in this study, conidial dimensions observed both in culture (CBS 770.83: 9.5–14 × 3–4.5 µm) and on the natural substrate (CBS H-3522: 10–13.5 × 3.5–4.5 µm) were highly similar and fall within the range of variability reported for this species from Europe (Ellis 1968; Holubová-Jechová 1972; this study). Although conidia from the Japanese material were somewhat shorter than those of the Czech specimens (10–18 × 3.5–6 µm on the natural substrate, this study), their overall morphology is indistinguishable, including the subtle characters such as the ornamentation of the outer conidial wall due to collapsing sheath, a feature not previously reported in the literature but clearly visible under DIC and PHC microscopy (Figs 7, 8). Notably, Matsushima (1975) reported three Japanese collections of *P. parvisporum* from decaying wood of *Lithocarpus edulis* and *Quercus* sp., with varying conidial sizes in culture: 14.5–25 × 4.5–6.5 µm and 10–16 × 4–6 µm. We therefore concluded that CBS 770.83 and the Czech material of *P. parvisporum* listed above are conspecific and represent a reliable reference for this species. Attempts to extract DNA from the six herbarium specimens from the Czech Republic were unsuccessful.

The ITS distribution data derived from the GlobalFungi database suggest that *P. parvisporum* has a broad ecological amplitude (Fig. 5), occurring across diverse biomes and climatic conditions, but with a notable concentration in aquatic environments in East Asia (China, Japan). Records from Spain confirm the distribution of the species in Europe, although based on published data (see above) the species has been repeatedly reported from several European countries. Records from North America, from where the species was also reported (Wang 2010), and other parts of the world (GBIF Secretariat 2023), are absent in the GlobalFungi database. This broad ecological and climatic range may indicate genuine ecological versatility, or alternatively, the existence of a species complex, as suggested by the variability in conidial length and occurrence on both herbaceous and woody hosts. To clarify its taxonomy, molecular data from additional strains collected across diverse geographical regions are essential for stabilising the species concept.

Among *Pleurophragmium* species, *P. parvisporum* is most comparable to *P. fluviale*, *P. luguense*, and *P. septatum* in its conidial morphology. These taxa share obovoid to fusoid-ellipsoid conidia that are broadly rounded at the apex, tapering to a narrow base with a hilum, predominantly 3-septate, and hyaline to subhyaline, becoming pale brown at maturity. In the absence of molecular data, distinguishing among these species would be highly challenging. For detailed comparison, see Table 3.

Interestingly, several biologically active compounds, collectively known as dactylfungins, were isolated from *P. parvisporum* (strain D500 obtained from a dead leaf in Japan) and were reported to exhibit antifungal activity against *Candida pseudotropicalis* and other fungi (Xaio et al. 1993). However, these findings should be interpreted with caution due to the simple morphology of the species and the recently recognised taxonomic ambiguity among preserved strains identified as *P. parvisporum*.

#### ﻿ Pleurophragmium pteridophytophilum

Taxon classificationAnimaliaSordarialesLasiosphaeriaceae

(Jing Y. Zhang, K.D. Hyde & Y.Z. Lu) Réblová & Hern.-Restr. comb. nov.

8242E2D6-13C8-5D51-AA89-09D763157D9E

860808

##### Basionym.

*Neomyrmecridium pteridophytophilum* Jing Y. Zhang, K.D. Hyde & Y.Z. Lu, Fungal Diversity 132: 375. 2025.

##### Typus.

CHINA • Guizhou Province, Zunyi City, Chishui County, Hushi Town, Chishui Alsophila Natural Reserve; on dead frond stalks of *Pteridaceae* sp. in terrestrial habitats; 28 Jul 2022; J. Y. Zhang BL9 (holotype GZAAS 23-0664, ex-type culture KUNCC 23-13858).

#### ﻿ Pleurophragmium septatum

Taxon classificationAnimaliaSordarialesLasiosphaeriaceae

(Crous) Réblová & Hern.-Restr. comb. nov.

E2991ED4-3F78-5646-8306-864C835570AB

860809

##### Basionym.

*Neomyrmecridium septatum* Crous, Persoonia 41: 287. 2018.

##### Typus.

THAILAND • Ratchaburi Province; on leaves of unidentified vine; 2008, P. W. Crous HPC 2252 (holotype CBS H-23768, culture ex-type CPC 34585 = CBS 145073).

#### ﻿ Pleurophragmium sichuanense

Taxon classificationAnimaliaSordarialesLasiosphaeriaceae

(Yan P. Chen & Maharachch.) Réblová & Hern.-Restr. comb. nov.

9DB3FBC1-91DB-5166-B64E-949CB559BE30

860810

##### Basionym.

*Neomyrmecridium sichuanense* Yan P. Chen & Maharachch., Phytotaxa 701(2): 134. 2025.

##### Typus.

CHINA • Sichuan Province, Chengdu City, Dujiangyan City, Longchi National Forest Park; elevation 702 m a.s.l.; 31°00.18'N, 103°38.77'E; on decaying branches of an unidentified herbaceous plant; 5 Oct 2021; Y. P. Chen & W. H. Tian LC64 (holotype HUEST 24.0068).

##### Notes.

There is a discrepancy between the protologue and the accompanying figures of *P. sichuanense* (Chen et al. 2025: fig. 3). While the conidia were described as subhyaline to pale brown, 0–1-septate, and finely verrucose, these features are not clear in the original image. The conidia appear hyaline, smooth, and 1–3-septate in the figures. Species with ornamented conidia are relatively rare in *Pleurophragmium* and currently include *P. parvisporum*, *P. sichuanense* along with two other species: *P. clavatum* (Ma et al. 2014) and *P. verruculosum* (Tiwari 1969).

#### ﻿ Pleurophragmium sorbicola

Taxon classificationAnimaliaSordarialesLasiosphaeriaceae

(Crous & R.K. Schumach.) Réblová & Hern.-Restr. comb. nov.

2FA35870-6E9C-5C3E-8772-DC54391DEC04

860811

##### Basionym.

*Myrmecridium sorbicola* Crous & R.K. Schumach., Fungal Syst. Evol. 1: 191. 2018.

##### Synonym.

*Neomyrmecridium sorbicola* (Crous & R.K. Schumach.) Crous, Persoonia 41: 287. 2018.

##### Typus.

GERMANY • near Berlin; on branch of *Sorbus aucuparia*; 17 Feb 2016; R. K. Schumacher (holotype CBS H-23405, culture ex-type CPC 30455 = CBS 143433).

###### ﻿*Pleurophragmium* s. lat.: species of uncertain status

Altogether, 19 species and varieties are recognised as members of Pleurophragmium s. lat. in this study. This section comprises a morphologically heterogeneous assemblage of species historically described in *Pleurophragmium*, but for which molecular data are lacking. While some species conform to the generic concept and remain plausible candidates for inclusion in *Pleurophragmium* s. str., others diverge considerably from it. Notably, only three species produce hyaline to subhyaline conidia, occasionally appearing fuscous in mass, whereas the remaining taxa bear pale brown, yellowish brown, brown, or dark brown conidia, often with distinctly paler end cell(s). To capture this variability and highlight inconsistencies in morphological characters, diagnostic features of *Pleurophragmium* s. lat. species currently retained in the genus are summarised in Table 3. However, the true affinities of these taxa cannot be reliably assessed until molecular data become available, emphasising the need for targeted recollection and sequencing.

We provide details on the holotype to facilitate future recollection of these species by fellow mycologists.

#### ﻿ Pleurophragmium angamosense

Taxon classificationAnimaliaSordarialesLasiosphaeriaceae

Matsush., Matshushima Mycol. Mem. 8: 30. 1995.

96FA22E8-6C1F-5AE9-89CE-827CEE7B7C36

##### Typus.

PERU • Colonia Angamos; on the decayed petiole of palm; Jul 1994; T. Matsushima (holotype MFC-4P738).

##### Notes.

The species differs from the *Pleurophragmium* concept primarily in the morphology of its conidiogenous cells and conidia. Although the cells proliferate sympodially, the conidiogenous loci are conspicuous, appearing as distinct scars 3–3.5 µm wide. Conidia are fusiform, transversely septate, smooth, and brown, with the terminal cells noticeably paler, and with dark, thick-walled septa. The original illustration by Matsushima (1995) further suggests that conidial secession may be rhexolytic, as remnants of the outer wall appear to remain attached both to the conidiogenous locus and to the basal part of the conidium. These features clearly contrast with *Pleurophragmium* s. str., in which conidiogenous loci are reduced to minute denticles and conidial secession is schizolytic.

#### ﻿ Pleurophragmium aquaticum

Taxon classificationAnimaliaSordarialesLasiosphaeriaceae

R.F. Castañeda, Heredia & R.M. Arias, Mycotaxon 101: 92. 2007.

8BE1BD93-9DB0-51D1-9255-9F775F6B00AC

##### Typus.

MEXICO • Veracruz, “Los Tuxtlas”; on decaying wood submerged in a stream; 19 May 2002; R. M. Arias & J. Y. C. Elizondo (holotype XAL CB743, isotype: MUCL 45625).

##### Notes.

Conidia formed on holoblastic-denticulate conidiogenous cells are fusiform to clavate, occasionally navicular, and often sub-umbonate at the apex. They are brown, becoming pale brown to subhyaline toward the ends and around the septa. By their shape and pigmentation, the conidia closely resemble those of *P. miniumbonatum* (Heredia et al. 2007). *Pleurophragmium aquaticum* also resembles *P. fusiforme* in having fusiform, pigmented conidia with paler ends, but the latter species differs in having narrower and uniformly pale brown conidia except the ends (without the paler bands at the septa).

#### ﻿ Pleurophragmium bitunicatum

Taxon classificationAnimaliaSordarialesLasiosphaeriaceae

Matsush., Icon. microfung. Matsush. lect.: 115. 1975.

F2FB9BD3-1697-5ED6-BBC7-AC554B48B242

##### Typus.

JAPAN • Tokyo Prefecture, Hachijo Island; on decaying leaves of unidentified dicotyledon plant; Feb 1969; T. Matsushima (holotype MFC-1669).

##### Notes.

The species is remarkably similar to members of *Aquapteridospora* (Yang et al. 2015). Another species, *P. bambusinum*, was recently transferred to *Aquapteridospora* based on molecular data (Bao et al. 2021).

#### ﻿ Pleurophragmium clavatum

Taxon classificationAnimaliaSordarialesLasiosphaeriaceae

L.G. Ma & X.G. Zhang, Mycotaxon 127: 216. 2014.

22BD8E93-873B-5C63-A9E4-97D08F9714A3

##### Typus.

CHINA • Yunnan Province, the Forbidden Forest of Banna; on dead branches of *Beilschmiedia percoriacea* (*Lauraceae*); 31 Oct 2011; L. G. Ma (holotype HSAUP H2090, isotype HMAS 243416).

##### Notes.

Ma et al. (2014) described three morphologically similar species, *P. clavatum*, *P. ellipsoideum*, and *P. yunnanense*, characterised by yellowish-brown to pale brown conidia and conidiophores that often terminate in a denticulate rachis and have distinct nodulose swellings along the whole length. These swellings, however, are not typical for *Pleurophragmium* based on observations of species on natural substrates, where the fertile region of the conidiophore is restricted to its upper part and bears terminal and intercalary conidiogenous cells. In culture, by contrast, we frequently observed such swellings in *P. parvisporum*. Conidia of *P. clavatum* are consistently aseptate, whereas in *Pleurophragmium* conidia are invariably septate, with both aseptate and multi-septate forms occurring within a single species. Molecular data are therefore needed to confirm the placement of these species in the genus.

#### ﻿ Pleurophragmium ellipsoideum

Taxon classificationAnimaliaSordarialesLasiosphaeriaceae

L.G. Ma & X.G. Zhang, Mycotaxon 127: 214. 2014.

9A20EA83-0885-50EC-ABBD-E66B9CD1CD89

##### Typus.

CHINA • Yunnan Province, the Forbidden Forest of Banna; on dead branches of *Bauhinia acuminata* L. (*Caesalpiniaceae*); 17 Oct 2008; L. G. Ma (holotype HSAUP H0042, isotype HMAS 243411).

##### Notes.

See notes under *P. clavatum*.

#### ﻿ Pleurophragmium indicum

Taxon classificationAnimaliaSordarialesLasiosphaeriaceae

D’Souza & Bhat, Mycotaxon 119: 477. 2012.

44C3E5D1-A217-5B68-AF63-E254FA18B35B

##### Typus.

INDIA • Goa, Molem Wildlife Sanctuary; on fallen dead and decaying leaves of *Dendrocalamus strictus* (*Poaceae*); 11 Mar 1999; M. A. D’Souza (holotype GUBH 367).

##### Notes.

The species is characterised by distinctly versicolorous conidia, with the middle cells dark brown, the end cells pale brown, and the septa conspicuously thick-walled and dark brown. In overall morphology, it bears resemblance to members of *Aquapteridospora* (Yang et al. 2015).

#### ﻿ Pleurophragmium harunganae

Taxon classificationAnimaliaSordarialesLasiosphaeriaceae

Hansf., Mycol. Pap. 15: 211. 1946.

133052DB-D682-548A-8B91-40F3059A7B01

##### Typus.

UGANDA • Entebbe Road, on sori of *Hemileia* on leaves of *Harungana madagascariensis*; C. G. Hansford 2803, 3013.

##### Notes.

The species is parasitic on the sori of *Hemileia (Pucciniales)*. It was described as having dematiaceous conidiophores with a terminal denticulate rachis, accompanied by additional, disconnected fertile regions along the conidiophore axis. Conidia are aseptate, curved, fusiform, and subhyaline to pale olivaceous. Based on these features, the species appears more consistent with morphologically similar genera such as *Ramichloridium* or *Myrmecridium*, pending recollection and the acquisition of molecular data to confirm its placement.

#### ﻿ Pleurophragmium malaysianum

Taxon classificationAnimaliaSordarialesLasiosphaeriaceae

Matsush., Matsushima Mycol. Mem. 9: 20. 1996.

06E24AF0-113C-58C9-8E0E-77CCD78F00C1

##### Typus.

MALAYSIA • Selangor Darul Ehsan, Ulu Gombak, The University of Malaya Field Stusy Centre; on decaying leaves of a deciduous tree; 12 Jun 1995; T. Matsushima (holotype MFC-5T054).

##### Notes.

The species is characterised by cylindrical to clavate, pseudoseptate conidia (Matsushima 1996), features that have not previously been observed in members of *Pleurophragmium* s. str.

#### ﻿ Pleurophragmium miniumbonatum

Taxon classificationAnimaliaSordarialesLasiosphaeriaceae

(R.F. Castañeda, Iturr. & Guarro) R.F. Castañeda, Mycotaxon 101: 96. 2007.

33AB0AFF-E4C8-5828-8FCC-2EFF900E76ED

##### Basionym.

*Cordana miniumbonata* R.F. Castañeda, Iturr. & Guarro, Mycotaxon 73: 5. 1999.

##### Typus.

VENEZUELA • Estado de Aragua, Parque Nacional “Henry Pittier”, Estación Rancho Grande, Camino de Interpretación de la Naturaleza “Andy Fields”, in undisturbed rain forest; on fallen decaying leaves on an unidentified plant; 25 Nov 1997; R. F. Castañeda & T. Iturriaga (holotype MUCL 40700).

##### Notes.

Heredia et al. (2007) transferred *C. miniumbonata* to *Pleurophragmium* based on its superficial resemblance to *P. aquaticum* (in the same study) and the presence of conidiophores with a terminal denticulate fertile region, a feature more consistent with *Pleurophragmium* than with *Cordana* (Preuss 1851; Hernández-Restrepo et al. 2014). The conidia of *P. miniumbonatum* are dark brown, except for the hyaline basal cell, and exhibit an umbonate apex with variable shapes ranging from obovoid and pyriform to broadly clavate. However, such dark brown conidia are atypical of *Pleurophragmium* under its current circumscription.

#### ﻿ Pleurophragmium naviculiforme

Taxon classificationAnimaliaSordarialesLasiosphaeriaceae

Matsush., Icon. microfung. Matsush. lect.: 115. 1975.

9C5C935F-FD87-5995-9272-227B351D5C9A

##### Typus.

JAPAN • Okinawa Prefecture, Iriomote island; on decaying leaves of an unidentified deciduous tree; Feb 1972; T. Matsushima (holotype MFC-4355).

##### Notes.

The species is characterised by 1-septate, navicular conidia acute at the apex and remain hyaline, both individually and in mass. The fertile region of the conidiophore is apical, relatively small, and often appears inflated and curved. Morphologically, the species resembles *P. naviculare* (Yang et al. 2023), but the latter differs in possessing a longer fertile region and smaller, verrucose conidia that become pale brown with hyaline end cells at maturity. Molecular data are needed to determine whether *P. naviculiforme* represents a species belonging to a distinct genus or falls within the interspecific variability of *Pleurophragmium*.

#### ﻿ Pleurophragmium obcampanuloides

Taxon classificationAnimaliaSordarialesLasiosphaeriaceae

Matsush., Matsushima Mycol. Mem. 8: 29. 1995.

0BD5CA4C-103C-5EDA-B145-5563CC3F11D2

##### Typus.

JAPAN • Okinawa Prefecture, Iriomote island; in litter on the forest floor; 1992; T. Matsushima (holotype MFC-2J039).

##### Notes.

Due to its unique obpyriform to turbinate conidia, which are septate, pale olivaceous, and olivaceous in mass (Matsushima 1995), this species is readily distinguished from other members of the genus.

#### ﻿ Pleurophragmium peruamazonicum

Taxon classificationAnimaliaSordarialesLasiosphaeriaceae

Matsush., Matsushima Mycol. Mem. 7: 61. 1993.

F732071E-9562-5B96-9E9F-D8F5FF6977C3

##### Typus.

PERU • Loreto, forest Rio Negro; on decaying palm leaves; Jun 1992; T. Matsushima (holotype MFC-2P079).

##### Notes.

The species is characterised by versicolorous, 3-celled conidia with a brown median cell and hyaline end cells. Its conidiophores bear an apical fertile region that often extends along the upper half of the conidiophore axis, with denticles sparsely distributed (Matsushima 1993). Morphologically, it is strikingly similar to *P. tricolor* (nom. inval., Art. 40.1; Rambelli 2009); for a detailed comparison, see Table 3. While *P. peruamazonensis* was described from culture, measurements of *P. tricolor* were derived from natural material. Consequently, features that distinguish the two taxa, such as conidial size and the extent of the apical fertile region, may simply reflect differences between *in vitro* and *in vivo* observations.

#### ﻿ Pleurophragmium peruamazonicum var. inflatum

Taxon classificationAnimaliaSordarialesLasiosphaeriaceae

Matsush., Matsushima Mycol. Mem. 7: 61. 1993.

87B32987-4DAF-5BAA-B05F-497F86E3D296

##### Typus.

PERU • Loreto, forest Rio Negro; on decaying leaves of a deciduous tree; Nov 1990; T. Matsushima (holotype MFC-0P570).

##### Notes.

The variety *inflatum* differs from the type variety by the conspicuous inflation of the basal portion of the conidium (Matsushima 1993). It is further characterised by thick-walled septa, with the two basal cells pale brown. However, it remains uncertain whether these distinctions fall within the natural intraspecific variability of the species or represent a separate species.

#### ﻿ Pleurophragmium subfusiforme

Taxon classificationAnimaliaSordarialesLasiosphaeriaceae

Matsush., Icon. Microfung. Matsush. lect.: 116. 1975.

77E41CD3-A777-5DB9-B595-20512305548D

##### Typus.

JAPAN • Mie Prefecture, University of Mie, Hirakura Exp. Forest; forest; Sep 1965; T. Matsushima (holotype MFC-1551).

##### Notes.

*Pleurophragmium subfusiforme* is readily distinguished from other species of the genus by its large (21–38 × 5.8–9.2 µm), fusiform conidia, which are 3–7-septate and sometimes slightly constricted at the septa. The conidia are subhyaline, becoming pale fuscous in mass (Matsushima 1975).

#### ﻿ Pleurophragmium taiwanense

Taxon classificationAnimaliaSordarialesLasiosphaeriaceae

Matsush., Matsushima Mycol. Mem. 5: 24. 1987.

0DD2C398-CE8C-54F3-A959-958AAFA557C3

##### Synonym.

*Pleurophragmium bicolor* Matsush., Icon. microfung. Matsush. lect.: 114. 1975. Nom. illegit. (ICN, Art. 53.1) non *Pleurophragmium bicolor* Costantin, Costantin, Mucéd. Simpl.: 100. 1888.

##### Typus.

TAIWAN • Ken-Ting Park; on rotten leaf-rachis of *Arenga engleri*; 27 May 1980; T. Matsushima (holotype MFC-10120).

##### Notes.

This species produces cylindro-clavate to somewhat turbinate conidia with a slightly inflated apical cell. Observations from culture show that the fertile regions of the conidiophore are discontinuous, occurring apically as well as at several points along the conidiophore axis, sometimes associated with nodose swellings (Matsushima 1987). Morphologically, it resembles *P. peruamazonicum* but can be distinguished by its shorter conidia and fewer septa. For additional comparison, see notes to *P. asiaticum*.

#### ﻿ Pleurophragmium tricolor

Taxon classificationAnimaliaSordarialesLasiosphaeriaceae

Rambelli, Flora Mediterranea 19: 82. 2009. Nom. inval. (ICN, Art. 40.1).

CDC0FE22-876B-5CE3-8DBE-5DC53709D9CB

##### Typus.

ITALY • Pantelleria, Montagna Grande; on dead leaves of *Arbutus unedo* (a7); (holotype PAL).

#### ﻿ Pleurophragmium varieseptatum

Taxon classificationAnimaliaSordarialesLasiosphaeriaceae

Matsush., Icon. microfung. Matsush. lect.: 117. 1975.

DBA4607D-1914-5959-99AC-31CAE8961D7B

##### Typus.

JAPAN • Kyoto City; on decaying stem of *Phyllostachys edulis*; Feb 1966; T. Matsushima (holotype MFC-1964).

##### Notes.

The species is readily distinguished by its cylindrical, pale olivaceous conidia, which display considerable variability in both size and septation, ranging from one to four septa (Matsushima 1975).

#### ﻿ Pleurophragmium verruculosum

Taxon classificationAnimaliaSordarialesLasiosphaeriaceae

D.P. Tiwari, Indian Phytopath.: 513. 1970.

A37A9233-759D-5E62-BB3B-BB2D198C38C3

##### Typus.

INDIA • Madhya Pradesh, Sagar; rhizosphere soil of *Piper betle*; Dec 1965; D. P. Tiwari (holotype IMI 134426, isotype ITCC New Delhi 1375).

##### Notes.

Verrucose conidia are rare in *Pleurophragmium* and are known only from a few species, namely *P. clavatum*, *P. sichuanense*, and *P. verruculosum*. Ornamented conidia are also observed in *P. pteridophytophilum* as “rough-walled” (Zhang et al. 2025), and in *P. parvisporum* (this study), where the conidial wall appears somewhat longitudinally crumpled. Among these taxa, molecular data are currently available only for *P. sichuanense* and *P. parvisporum*. Morphologically, *P. verruculosum* is most similar to *P. clavatum*, but can be distinguished by its septate conidia and shorter conidiophores.

#### ﻿ Pleurophragmium yunnanense

Taxon classificationAnimaliaSordarialesLasiosphaeriaceae

L.G. Ma & X.G. Zhang, Mycotaxon 127: 215. 2014.

785A4CDE-49F9-5078-B053-049FB1B22D41

##### Typus.

CHINA • Yunnan Province, the Forbidden Forest of Banna; on dead branches of *Machilus salicina* (*Lauraceae*); 18 Oct 2008; L. G. Ma (holotype HSAUP H0086, isotype HMAS 243412).

##### Notes.

The species produces pale brown, broadly fusiform conidia tapering at both ends, which are mostly 3-septate (Ma et al. 2014). In conidial size and septation, it resembles *P. ellipsoideum* from the same study, but the latter differs in having ellipsoidal conidia that are broadly rounded at the apex. For additional information, see the notes under *P. clavatum*.

###### ﻿Species excluded from *Pleurophragmium* and described in other genera

Here, we provide a list of species that have been excluded from *Pleurophragmium* and subsequently transferred to other genera by various authors. Synonymy follows the records in MycoBank, unless stated otherwise.

#### ﻿ Aquapteridospora bambusinum

Taxon classificationAnimaliaDistoseptisporalesAquapteridosporaceae

(D.Q. Dai & K.D. Hyde) D.F. Bao, J. Fungi 7(669): 10. 2021.

408303D8-2675-5F6B-A878-C5B552C2B02B

##### Basionym.

*Pleurophragmium bambusinum* D.Q. Dai & K.D. Hyde, Fungal Diversity 82: 92. 2016.

#### ﻿ Dactylaria arecae

Taxon classificationAnimaliaHelotialesHelotiaceae

(Matsush.) R.F. Castañeda & W.B. Kendr., Univ. Waterloo, Biol. Ser. 35: 26. 1991.

CAD0D78D-A63E-5D13-8C69-C30B0CF758D0

##### Basionym.

*Pleurophragmium arecae* Matsush., Matsushima Mycol. Mem. 5: 23. 1987.

#### ﻿ Dactylaria cylindrospora

Taxon classificationAnimaliaHelotialesHelotiaceae

(Matsush.) R.F. Castañeda & W.B. Kendr., Univ. Waterloo, Biol. Ser. 35: 27. 1991.

14200AFF-1CD8-503E-8ECF-35B7A9E4A9F6

##### Basionym.

*Pleurophragmium cylindrosporum* Matsush., Icon. microfung. Matsush. lect.: 115. 1975.

#### ﻿ Dactylaria triseptata

Taxon classificationAnimaliaHelotialesHelotiaceae

(Matsush.) R.F. Castañeda & W.B. Kendr., Univ. Waterloo, Biol. Ser. 35: 47. 1991.

0E769E43-3E10-5DDB-89B7-9BE7BB16BE8E

##### Basionym.

*Pleurophragmium triseptatum* Matsush., Icon. microfung. Matsush. lect.: 116. 1975.

#### ﻿ Helminthosporium flumeanum

Taxon classificationAnimaliaPleosporalesMassarinaceae

Sacc., Syll. fung. 25: 821. 1931.

4121B26A-6990-5C9D-8378-92780432B313

##### Basionym.

*Pleurophragmium flumeanum* (Sacc.) S. Hughes, Canad. J. Bot. 36: 797. 1958.

#### ﻿ Minimelanolocus leptotrichus

Taxon classificationAnimaliaChaetothyrialesHerpotrichiellaceae

(Cooke & Ellis) R.F. Castañeda & Heredia, Cryptog. Mycol. 22: 10. 2001.

33BCBD76-C1C0-5D33-8D5E-E5A572D46AE6

##### Basionym.

*Helminthosporium leptotrichum* Cooke & Ellis, Grevillea 8(45): 13. 1879.

##### Synonyms.

*Brachysporium leptotrichum* (Cooke & Ellis) Sacc., Syll. fung. 4: 425. 1886.

*Pleurophragmium leptotrichum* (Cooke & Ellis) S. Hughes, Canad. J. Bot. 36: 797. 1958.

*Pseudospiropes leptotrichus* (Cooke & Ellis) M.B. Ellis, More dematiaceous *Hyphomycetes*: 2244. 1976.

#### ﻿ Minimelanolocus subulifer

Taxon classificationAnimaliaChaetothyrialesHerpotrichiellaceae

(Corda) R.F. Castañeda & Heredia, Cryptog. Mycol. 22: 9. 2001.

E9228506-14EB-5330-B45B-130F63931E67

##### Basionym.

*Helminthosporium subuliferum* Corda, Icon. Fung. 1: 13. 1837.

##### Synonyms.

*Pleurophragmium subuliferum* (Corda) S. Hughes, Canad. J. Bot. 36: 798. 1958.

*Pseudospiropes subuliferus* (Corda) M.B. Ellis, More dematiaceous *Hyphomycetes*: 220. 1976.

#### ﻿ Myrmecridium schulzeri

Taxon classificationAnimaliaMyrmecridialesMyrmecridiaceae

(Sacc.) Arzanlou, W. Gams & Crous, Stud. Mycol. 58: 84. 2007

F222EECF-1EC8-51FF-A1DD-1B0BC874E9B6

##### Basionym.

*Psilobotrys schulzeri* Sacc., Hedwigia 23: 126. 1884.

##### Synonyms.

*Chloridium schulzeri* (Sacc.) Sacc., Syll. fung. 4: 322. 1886.

*Ramichloridium schulzeri* (Sacc.) de Hoog, Stud. Mycol. 15: 64. 197.

*Rhinocladiella schulzeri* (Sacc.) Matsush., Icon. microfung. Matsush. lect.: 124. 1975.

*Acrotheca acuta* Grove, J. Bot. 54: 222. 1916.

*Pleurophragmium acutum* (Grove) M.B. Ellis, More dematiaceous *Hyphomycetes*: 164. 1976.

*Rhinotrichum multisporum* Doguet, Rev. Mycol. 17: 78. 1952.

*Acladium multisporum* (Doguet) Bat. & Oliveira, Anais Congr. Soc. Bot. Brasil: 347. 1964.

#### ﻿ Myrmecridium schulzeri var. tritici

Taxon classificationAnimaliaMyrmecridialesMyrmecridiaceae

(M.B. Ellis) Arzanlou, W. Gams & Crous, Stud. Mycol. 58: 84. 2007.

AF5CC3E3-8DFA-5416-A32A-AAB6B76734FB

##### Basionym.

*Pleurophragmium tritici* M.B. Ellis, More dematiaceous *Hyphomycetes*: 165. 1976.

##### Synonym.

Ramichloridium schulzeri var. tritici (M.B. Ellis) de Hoog, Stud. Mycol. 15: 68. 1977.

#### ﻿ Pseudospiropes costaricensis

Taxon classificationAnimaliaHelotialesHelotiaceae

(E.F. Morris) de Hoog & Arx, Kavaka 1: 59. 1974.

065FFB14-E481-5705-8558-A1A31C1FD43E

##### Basionym.

*Pleurophragmium costaricensis* E.F. Morris, Mycologia 64: 893. 1972.

##### Notes.

Iturriaga and Korf (1990) proposed the dematiaceous hyphomycete genus *Pseudospiropes* (Ellis 1971), typified by *P. nodosus*, as a generic synonym of the apothecial ascomycete genus *Strossmayeria* (Schulzer 1881) (*Helotiales*). The connection between the sexual and asexual morphs was initially established experimentally for two species of *Strossmayeria* sp. by Iturriaga and Korf (1984), and later demonstrated for *P. josserandii*, *P. nodosus*, *P. simplex*, several additional *Pseudospiropes* species, and taxa referred to as the “*P. nodosus* type” and the “*P. simplex* complex” (Iturriaga and Korf 1990). The remaining species of *Pseudospiropes* still require critical revision. Based on Morris’s (1972) original description and illustration of *P. costaricensis*, we suggest that this species is more appropriately accommodated in *Strossmayeria* than *Pleurophragmium*, pending revision of the holotype and the availability of its molecular data.

#### ﻿ Spiropes capensis

Taxon classificationAnimalia

(Thüm.) M.B. Ellis, Mycol. Pap. 114: 5. 1968.

12660EDB-D28D-5AA9-86A0-F0A7FFE2AB6C

##### Basionym.

*Helminthosporium capense* Thüm., Flora 59: 570. 1876.

##### Synonyms.

*Cercospora capensis* (Thüm.) Sacc., Syll. fung. 4: 469. 1886.

*Pleurophragmium capense* (Thüm.) S. Hughes, Canad. J. Bot. 36: 796. 1958.

For additional synonyms, see Ellis (1968).

#### ﻿ Spiropes dorycarpus

Taxon classificationAnimalia

(Mont.) M.B. Ellis, Mycol. Pap. 114: 11. 1968.

37E81EC7-0122-5B69-BDFA-436A14FC89D5

##### Basionym.

*Helminthosporium dorycarpum* Mont., Ann. Sci. Nat., Bot. 17: 120. 1842.

##### Synonym.

*Pleurophragmium dorycarpum* (Mont.) S. Hughes, Canad. J. Bot. 36: 797. 1958.

For additional synonyms, see Ellis (1968).

#### ﻿ Spiropes effusus

Taxon classificationAnimalia

(Pat.) M.B. Ellis, Mycol. Pap. 114: 10. 1968.

3C0B6D2B-66F7-5476-AFC6-87A30C1E5139

##### Basionym.

*Podosporium effusum* Pat., Sci. Surv. Porto Rico & Virgin Islands 8(1): 103. 1926.

##### Synonyms.

Helminthosporium dorycarpum var. amazoniae S. Hughes, Mycol. Pap. 50: 24. 1953.

Pleurophragmium dorycarpum var. amazoniae (S. Hughes) S. Hughes, Canad. J. Bot. 36: 797 1958.

#### ﻿ Spiropes guareicola

Taxon classificationAnimalia

(F. Stevens) Cif., Sydowia 9: 303. 1955.

5518A38D-06C1-5A03-A106-8DAA195F263E

##### Basionym.

*Helminthosporium guareicola* F. Stevens, Bot. Gaz. 65(3): 241. 1918.

##### Synonyms.

*Pleurophragmium guareicola* (F. Stevens) S. Hughes, Canad. J. Bot. 36: 797. 1958.

*Helminthosporium flagellatum* H.S. Yates, Philipp. J. Sci., C 13(6): 383. 1918.

*Helminthosporium spirotrichum* Sacc., Bull. Orto Bot. Regia Univ. Napoli 6: 61. 1921.

Cladosporium elegans var. singaporense Sacc., Bull. Orto Bot. Regia Univ. Napoli 6: 60. 1921.

#### ﻿ Spiropes helleri

Taxon classificationAnimalia

(F. Stevens) M.B. Ellis, Mycol. Pap. 114: 14. 1968.

3AE5AC88-3003-5EB9-90C3-BAE632EC5CD8

##### Basionym.

*Helminthosporium helleri* F. Stevens, Bot. Gaz. 65: 242. 1918.

##### Synonyms.

*Helminthosporium maculosum* Sacc., Atti Accad. Sci. Ven.-Trent.-Istr., Sér. 3 10: 91. 1919. [1917].

*Pleurophragmium maculosum* (Sacc.) S. Hughes, Canad. J. Bot. 36: 797. 1958.

*Helminthosporium leucosykes* H.S. Yates, Philipp. J. Sci., C 13(6): 382. 1918.

#### ﻿ Spiropes palmetto

Taxon classificationAnimalia

(W.R. Gerard) M.B. Ellis, Mycol. Pap. 114: 16. 1968.

95594F82-BBBC-5064-8FAA-45BEEA1D5902

##### Basionym.

*Helminthosporium palmetto* W.R. Gerard, Grevillea 17(83): 68. 1889.

##### Synonym.

*Pleurophragmium palmetto* (W.R. Gerard) S. Hughes, Canad. J. Bot. 36: 798. 1958.

#### ﻿ Spiropes scopiformis

Taxon classificationAnimalia

(Berk.) M.B. Ellis, Mycol. Pap. 114: 30. 1968.

416B757E-7FDE-5ACD-B72F-E7774CFEBE35

##### Basionym.

*Cladosporium scopiforme* Berk. [as ‘*scopaeforme*’], Hooker’s J. Bot. Kew Gard. Misc. 6: 208. 1854.

##### Synonyms.

*Helminthosporium scopiforme* (Berk.) Subram., J. Indian Bot. Soc. 35: 450. 1956.

*Pleurophragmium scopiforme* (Berk.) S. Hughes, Canad. J. Bot. 36: 798. 1958.

*Cladosporium congestum* Berk. & Broome, J. Linn. Soc., Bot. 14: 9. 1875.

#### ﻿ Strossmayeria atriseda

Taxon classificationAnimaliaHelotialesHelotiaceae

(Saut.) Iturr., Mycotaxon 36: 404. 1990.

775A6A0F-AF08-5C54-A73A-51708A2416F6

##### Basionym.

*Peziza atriseda* Saut., Flora, Regensburg 28: 133. 1845.

##### Synonyms.

*Tapesia atriseda* (Saut.) Poetsch & Schied., System. Aufzähl. samenlos. Pflanzen (Krypt.): 158. 1872.

*Helminthosporium nodosum* Wallr., Fl. crypt. Germ. 2: 165. 1833.

*Brachysporium nodosum* (Wallr.) Sacc., Syll. fung. 4: 425. 1886.

*Pleurophragmium nodosum* (Sacc.) S. Hughes, Canad. J. Bot. 36: 797. 1958.

*Pseudospiropes nodosus* (Sacc.) M.B. Ellis, Dematiaceous *Hyphomycetes*: 258. 1971.

*Helminthosporium subapiculatum* Peck, Bull. New York State Mus. 150: 55. 1911.

#### ﻿ Strossmayeria basitricha

Taxon classificationAnimaliaHelotialesHelotiaceae

(Sacc.) Dennis, British cup fungi and their allies: An introduction to the Ascomycetes: 73: 1960.

75606A4D-851D-5471-A235-78A6B32E5C27

##### Basionym.

*Belonidium basitrichum* Sacc., Atti Soc. Veneto-Trentino Sci. Nat. Padova 4(1): 135. 1875.

##### Synonyms.

*Helminthosporium cylindricum* Wallr. [as ‘*Helmisporium*’], Fl. crypt. Germ. 2: 164. 1833. Nom. illegit. (ICN, Art. 53.1) non *Helminthosporium cylindricum* Corda [as ‘*Helmisporium*’], Deutschlands Flora, Abt. III. Die Pilze Deutschlands 3 (11): 21, t. 11. 1831.

*Pleurophragmium cylindricum* S. Hughes, Canad. J. Bot. 36: 797. 1958.

*Pseudospiropes simplex* (Kunze ex Nees) M.B. Ellis, Dematiaceous *Hyphomycetes*: 260. 1971.

For additional synonyms, see Hughes (1958), Ellis (1971) and Iturriaga and Korf (1990).

#### ﻿ Thysanorea rousseliana

Taxon classificationAnimaliaChaetothyrialesHerpotrichiellaceae

(Mont.) Hern.-Restr. & Crous, Fungal Syst. Evol. 6: 18. 2020.

16687322-07E0-5E38-A55B-7F297CECDF9B

##### Basionym.

*Helminthosporium rousselianum* Mont., Ann. Sci. Nat., Bot. Sér. 3, 12: 300. 1849.

##### Synonyms.

*Pleurophragmium rousselianum* (Mont.) S. Hughes, Canad. J. Bot. 36: 798. 1958.

*Pseudospiropes rousselianus* (Mont.) M.B. Ellis, More dematiaceous *Hyphomycetes*: 221. 1976.

*Spiropes rousselianus* (Mont.) de Hoog & Arx, Kavaka 1: 59. 1974.

*Minimelanolocus rousselianus* (Mont.) R.F. Castañeda & Heredia, Cryptog. Mycol. 22: 10. 2001.

#### ﻿ Virgariella hippotrichoides

Taxon classificationAnimaliaPhacidialesHelicogoniaceae

(Corda) de Hoog, Stud. Mycol. 15: 205. 1977.

0641C641-C4F7-5318-B5F7-8DD5C069612C

##### Basionym.

*Chloridium hippotrichoides* Corda, Icon. Fung. 1: 17, t. 4: 238. 1837.

##### Synonym.

*Pleurophragmium hippotrichoides* (Corda) M.B. Ellis, More dematiaceous *Hyphomycetes*: 164. 1976.

#### ﻿ Skoliomycella

Taxon classificationAnimalia

Réblová & Hern.-Restr. gen. nov.

28A84564-EC8F-5CF4-BB0E-476A4FACB7D7

860717

##### Etymology.

From Greek *skolios* (crooked, bent, or twisted) and the Latinised diminutive suffix -*mycella*, derived from Greek *mykēs* (fungus). Referring to a “small crooked fungus” with characteristically bent, geniculate or flexuous conidiophores observed in culture.

##### Type species.

*Skoliomycella flava* Réblová & Hern.-Restr.

##### Description.

**Sexual morph.** Not observed. **Asexual morph. *Colonies****in vitro* effuse. ***Mycelium*** composed of hyaline or lightly pigmented, septate hyphae. ***Conidiophores*** macronematous, mononematous, sometimes reduced to a single conidiogenous cell, erect, cylindrical, unbranched, flexuous to sinuous, sometimes becoming geniculate exhibiting a zig-zag pattern, percurrently elongating, pigmented, septate. ***Conidiogenous cells*** integrated, terminal and intercalary, monoblastic or polyblastic, extending sympodially, with denticles; conidiogenesis holoblastic-denticulate. ***Conidia*** solitary, dry, acropleurogenous, oblong to fusiform to ellipsoidal, pigmented, transversely septate; conidial secession schizolytic.

##### Notes.

*Skoliomycella*, typified by *S. flava*, known to reproduce only asexually, is proposed here as a new monotypic genus in the *Tubeufiaceae*. In overall morphology, it resembles *Camporesiomyces* (Hyde et al. 2020; Han et al. 2025) and *Zaanenomyces* (Crous et al. 2021), which, together with *Skoliomycella* and several other genera, form a well-supported subclade within the *Tubeufiaceae*.

#### ﻿ Skoliomycella flava

Taxon classificationAnimalia

Réblová & Hern.-Restr. sp. nov.

B31D378C-879F-5661-9575-D111A23F89EC

860718

Fig. 9

##### Etymology.

From Latin *flavus* (yellow). Referring to the yellow pigment released by the colonies into the surrounding agar.

##### Typus.

PORTUGAL • Minho province, Melgaço, Fonte de São João; unidentified plant debris; Nov 2007; J. Capilla, R. F. Castañeda-Ruiz & C. Silvera (holotype CBS H-25776 dried culture, ex-type culture CBS 122759 = FMR 9646).

##### Culture characteristics.

On CMD colonies 9–10 mm diam., circular, raised, margin entire or slightly undulate, floccose to velvety becoming mucoid, zonate, olivaceous-brown, ochre at the margin surrounded by isabelline halo, with a prominent submerged growth, reverse beige-yellow. On MLA colonies 10–12 mm diam., circular, convex, margin undulate, velvety, zonate, whitish-purple to whitish-brown with an outer thin purple zone, ochre-cream at the margin, with a submerged growth, pale yellow pigment diffusing into the agar, reverse ochre. On OA colonies 9–10 mm diam., circular, flat, margin undulate, velvety, off-white to whitish-brown, brown towards the periphery, with pale ochre-brown halo, pale ochre pigment diffusing into the agar, reverse ochre-brown. On PCA colonies 8–10 mm diam., circular, convex, margin undulate, velvety, zonate, cream to beige-brown, with thin purple-brown and golden-ochre zones at the margin, pale yellow pigment diffusing into agar, reverse ochre. Sporulation absent on CMD, moderate on MLA, OA and PCA.

##### Description in culture.

***Colonies*** on MLA effuse. **Sexual morph.** Not observed. **Asexual morph. *Mycelium*** composed of hyaline, subhyaline to pale brown, branched, septate hyphae, 1.5–2.5 µm wide, occasionally slightly swollen. ***Conidiophores*** (22–)35–102 × 3–5 µm, macronematous, mononematous, sometimes reduced to a single conidiogenous cell without or with 1–2 supporting cells, scattered or loosely aggregated, erect, cylindrical, some cells slightly inflated, unbranched, sometimes percurrently elongating, flexuous to sinuous, sometimes becoming geniculate exhibiting a zig-zag pattern due to local bending above the septum, each bend is associated with the formation of a single denticle on the ‘outside’ giving the appearance of irregular or dichotomous branching, pale brown to golden brown, smooth-walled, septate. ***Conidiogenous cells*** 10–24 × 3–4 µm, integrated, terminal, form transverse septa during sympodial extension and become intercalary, occasionally lateral growing directly on hyphae, monoblastic or polyblastic with 1–4 peg-like denticles, cylindrical or subulate, subhyaline to pale olivaceous-brown when in the terminal position, golden-brown when intercalary, smooth-walled; conidiogenesis holoblastic-denticulate. ***Conidia*** 18.5–36(–45) × (3–)4–5.5 µm (mean ± SD = 27.1 ± 5.6 × 4.9 ± 0.1 μm), solitary, dry, acropleurogenous, oblong to cylindrical, elongate fusiform to narrowly ellipsoidal, tapering at both ends, truncate at the base 1–1.5 µm wide, with a conspicuous basal scar, usually straight, occasionally slightly curved, often with guttules or granules visible inside the cells, smooth-walled, aseptate and hyaline when young, at maturity with 3–7 transverse septa and subhyaline to pale olivaceous-brown, olivaceous-grey in mass; conidial secession schizolytic.

**Figure 9.**
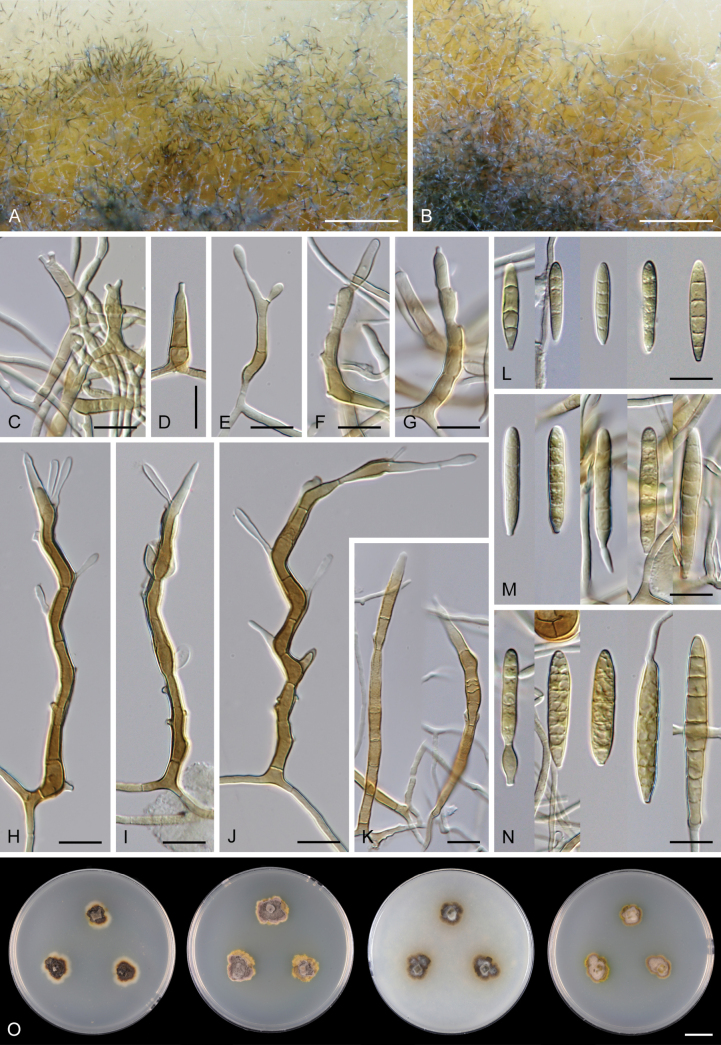
*Skoliomycella flava* (ex-type CBS 122759). **A, B** Sporulating conidiophores **C–G** conidiogenous cells with conidia **H–K** conidiophores with conidiogenous cells and conidia **L–N** conidia **O** diversity of colony morphology on CMD, MLA, OA, and PCA, respectively (from left to right) after 4 wk. Images: on MLA (A–N). Scale bars: 300 µm (**A, B**); 10 µm (**C–N**); 1 cm (**O**).

##### Habitat and geographical distribution.

*Skoliomycella flava* is a saprobe occurring on plant remnants. To date, two confirmed records originate from the Iberian Peninsula, specifically from Portugal (this study) and Spain (GlobalFungi), within a temperate, Mediterranean climate. According to GlobalFungi, it was detected in a single air sample from an anthropogenic biome, MAT ~14.5 °C, MAP ~575 mm/year.

##### Notes.

*Skoliomycella flava* is readily distinguished from the morphologically similar species attributed to *Camporesiomyces* (Hyde et al. 2020) and *Zaanenomyces* (Crous et al. 2021) by the absence of a rachis in the fertile apical portion of the conidiogenous cell. Instead, its conidiophores are flexuous to sinuous, sometimes exhibit a zig-zag pattern, with a single denticle and/or a slightly prolonged conidiogenous cell formed at each bend on the outer side of the conidiophore, giving the impression of irregular or dichotomous branching.

The detection of *S. flava* in urban air highlights its capacity for aerial dispersal, although it occurs at very low relative abundance. However, its global distribution remains uncertain, as no additional records outside the Iberian Peninsula are available. Whether *S. flava* represents a rare, geographically restricted lineage, or whether its scarcity reflects limited sampling, or under-detection due to low environmental abundance, remains to be determined.

#### ﻿ Thysanorea

Taxon classificationAnimaliaChaetothyrialesHerpotrichiellaceae

Arzanlou, W. Gams & Crous, Stud. Mycol. 58: 80. 2007. emend. Hern.-Restr. & Crous, Fungal Syst. Evol. 6: 17. 2020.

EF3E7F08-2869-51A0-BE5B-22FAA699ACC0

##### Description.

See Arzanlou et al. (2007) and Hernández-Restrepo et al. (2020).

##### Notes.

*Thysanorea* is characterised by micro- to macronematous, dematiaceous conidiophores that are simple or apically branched, bearing terminal or intercalary, holoblastic, polyblastic conidiogenous cells. Conidia are solitary, acropleurogenous, transversely septate, pale brown, and variable in shape, most commonly oblong, obovoid, or fusiform. In contrast, the associated synasexual morph is phialidic.

Three species previously classified in *Uncispora* were shown to be congeneric with *Thysanorea* and are here formally transferred to the genus, with new combinations proposed below.

#### ﻿ Thysanorea acropleurogena

Taxon classificationAnimaliaChaetothyrialesHerpotrichiellaceae

Réblová & Hern.-Restr. sp. nov.

CC282616-9A68-5EC3-9718-A2E0E627A1AB

860719

Fig. 10

##### Etymology.

From Greek *akros* (apex), *pleurá* (side) and *genēs* (born, produced). Referring to the mode of conidium development, in which conidia are formed both terminally and laterally on the conidiogenous cells.

##### Typus.

PAPUA NEW GUINEA • Madang Province, foothills of Finisterre Range, 40.8 km along road to Lae; 200 m a.s.l.; on unidentified wood; 2 Nov 1995; A. Aptroot 36665 (holotype CBS H-25780 dried culture, ex-type culture CBS 215.96).

##### Culture characteristics.

On CMD colonies 44–45 mm diam., circular, flat, margin entire, lanose, pinkish-brown to brown, darker at the margin, reverse dark brown. On MLA colonies 36–38 mm diam., circular, convex, margin entire, floccose to lanose, olivaceous-grey to mouse grey, dark olivaceous at the margin, reverse dark olivaceous. On OA colonies 40–41 mm diam., circular, flat, margin entire, lanose at the centre and on the inoculation block, cobwebby towards the periphery, with a well-defined grey-brown central zone, dark brown towards the periphery, with a diffuse lighter halo at the margin, reverse dark brown. On PCA colonies 40–42 mm diam., circular, flat, margin entire, lanose, grey-brown, cobwebby and dark brown towards the periphery, reverse dark brown. With a prominent submerged growth on all media. Sporulation on PCA, absent on CMD, MLA and OA.

##### Description in culture.

***Colonies*** on PCA effuse. **Sexual morph.** Not observed. **Asexual morph. *Mycelium*** composed of subhyaline to pale olivaceous brown, septate hyphae, 1.5–2.5 µm wide. ***Conidiophores*** 98–145 × 3.5–4.5 µm, basal cells sometimes slightly inflated 5–6.5 µm wide, macronematous, mononematous, loosely scattered to densely fasciculate, mostly erect, straight to slightly flexuous, cylindrical, subtly undulate, unbranched to loosely branched in the upper part, pale to medium brown to pale olivaceous brown, darker at the base, smooth-walled, septate. ***Conidiogenous cells*** 23.5–28(–40) × 3.5–5(–5.5) µm, integrated, terminal, forming transverse septa during sympodial proliferation and often becoming intercalary, arranged either along most of the conidiophore length or restricted to the upper part, forming a rachis with numerous, closely spaced, minute denticles, polyblastic, cylindrical, pale brown to pale olivaceous brown, end cells tend to be paler, smooth-walled; conidiogenesis holoblastic-denticulate. ***Conidia*** (13.5–)15–21 × (3.5–)4–5.5 µm (mean ± SD = 17.2 ± 1.8 × 4.5 ± 0.3 μm), solitary, dry, acropleurogenous, subcylindrical to oblong or fusiform to fusiform-clavate, tapering towards both ends, truncate at the base 1–1.5 µm wide, with a conspicuous basal scar, straight or slightly curved, pale brown to pale olivaceous brown, darker at the base, dark olivaceous brown in mass, smooth-walled, (1–)3-septate; conidial secession schizolytic.

**Figure 10.**
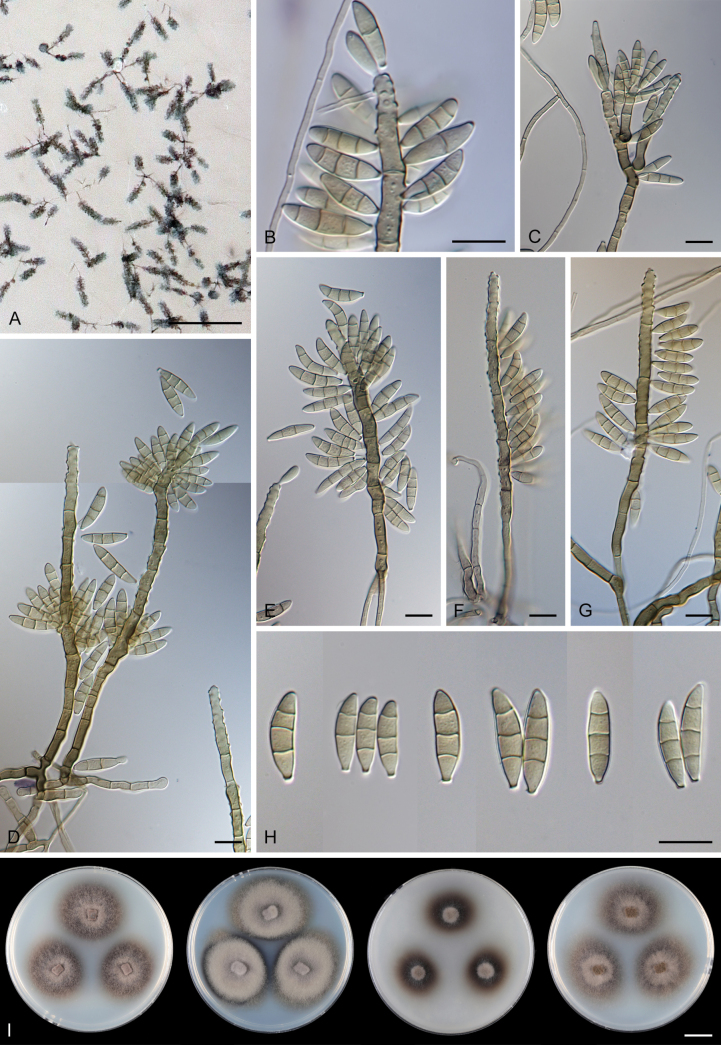
*Thysanorea acropleurogena* (ex-type CBS 215.96). **A** Sporulating conidiophores **B, C** upper part of the conidiophore conidiogenous cells forming a rachis and attached conidia **D–G** conidiophores, conidiogenous cells and conidia **H** conidia **I** diversity of colony morphology on CMD, MLA, OA, and PCA, respectively (from left to right) after 4 wk. Images: on PCA (**A–H**). Scale bars: 300 µm (**A**); 10 µm (**B–H**); 1 cm (**I**).

##### Habitat and geographical distribution.

The species is a saprobe occurring on decaying wood and is currently known only from Papua New Guinea. No identical ITS sequences were recovered in the GlobalFungi database.

##### Notes.

Phylogenetic analyses (Fig. 2) revealed that *T. acropleurogena* forms a sister relationship to a subclade comprising three closely related species, *T. hainanensis*, *T. sinensis*, and *T. wuzhishanensis*. They belong to a subclade that also includes *T. seifertii* and *T. cantrelliae*. Among known species, *T. acropleurogena* is closest to *T. obscura* and *T. seifertii* in having lightly pigmented, subcylindrical to oblong, predominantly 3-septate conidia. However, both species are readily distinguishable in conidial characters and having unbranched conidiophores. *Thysanorea obscura* differs from the new species in subhyaline to yellowish brown, larger conidia, 20–31 × 5–8 µm (Matsushima 1983). *Thysanorea seifertii* produces similar pale brown, subcylindrical to clavate or oblong conidia, but these are considerably shorter, 7–15 × 1.5–3 µm (Hernández-Restrepo et al. 2020). In addition, *T. seifertii* produces a phialidic synasexual morph in culture, a feature so far unique among *Thysanorea* species. In the phylogenetic tree, all three species are resolved as distinct lineages.

The original material, CBS H-6267, from which the axenic culture was derived, consists of multiple twigs. However, we could not locate the target fungus. Several twigs are covered with aerial mycelium, while others carry fertile conidiophores of a dendryphiella-like fungus. Accordingly, a dried culture was designated as the holotype of *T. acropleurogena*.

*Thysanorea acropleurogena* appears to represent a rare species, confirmed thus far from a single collection on decaying wood. The absence of identical ITS sequences in the GlobalFungi database indicates that it has not yet been detected in environmental sequencing datasets, suggesting that the species may be geographically restricted or currently overlooked due to under-sampling.

#### ﻿ Thysanorea hainanensis

Taxon classificationAnimaliaChaetothyrialesHerpotrichiellaceae

(Jian. Y. Li & Z.F. Yu) Réblová & Hern.-Restr. comb. nov.

1E14B3C9-5889-5953-801E-CEB5BEC79653

860812

##### Basionym.

*Uncispora hainanensis* Jian. Y. Li & Z.F. Yu, Mycotaxon 129: 474. 2015.

##### Typus.

CHINA • Hainan Province, Wuzhishan National Nature Reserve; 754 m a.s.l., isolated from decayed leaves; Dec 2011; G. Z. Yang (holotype YMF 1.04038, ex-type culture YMF1.040381).

#### ﻿ Thysanorea melanica

Taxon classificationAnimaliaChaetothyrialesHerpotrichiellaceae

(Hong Y. Su, Udayanga & K.D. Hyde) Hern.-Restr. & Crous, Fungal Syst. Evol. 6: 18. 2020.

819FCD0C-39F4-57FE-A842-3F3B8B7AB33D

##### Basionym.

*Minimelanolocus melanicus* Hong Y. Su, Udayanga & K.D. Hyde, Fungal Biol. 119: 1056. 2015.

##### Culture characteristics.

On CMD colonies 51–52 mm diam., circular, flat to slightly raised in the centre, margin diffuse, entire, zonate, floccose to velvety, white-beige in the centre, brown to dark brown towards the margin, reverse dark brown to black. On MLA colonies 49–51 mm diam., circular, flat, raised at the centre, margin entire, zonate, lanose to floccose centrally, becoming mucoid and glossy towards the periphery, lanose at the margin, cream to buff in the central region, sharply contrasting with the surrounding, dark grey to blackish mucoid mycelium, olivaceous-grey at the margin, reverse dark olivaceous-grey to black. On OA colonies 47–50 mm diam., circular, flat, margin diffuse, entire, floccose to slightly lanose at the centre, becoming cobwebby towards the periphery, whitish to pale pinkish-buff, surrounded by a wide zone of submerged, dark olivaceous-grey mycelium that diffuses into the agar, paler at the margin, reverse uniformly dark olivaceous-grey to black. On PCA colonies 41–42 mm diam., circular, flat to slightly raised in the centre, margin diffuse, entire, floccose to somewhat lanose, central zone pale pink-brown-buff, surrounded by a dark brown submerged zone, paler at the margin, reverse dark olivaceous-brown to nearly black. Sporulation absent on all media.

##### Description in culture.

***Colonies*** on PCA effuse. **Sexual morph.** Not observed. **Asexual morph. *Mycelium*** composed of subhyaline to pale brown, septate hyphae, 1.5–3 µm wide. Conidiophores, conidiogenous cells and conidia absent.

##### Specimen examined.

THE NETHERLANDS • North Holland Province, Wieringermeer Polder, Van Bemmelen Hoeve; isolated from wheat field soil; May 1966; W. Gams (living culture CBS 862.68).

##### Habitat and geographical distribution.

*Thysanorea melanica* was originally described from decaying wood in China (Liu et al. 2015), with an additional record from wheat field soil in the Netherlands (this study). According to the GlobalFungi database, the species has been detected in 266 environmental samples across three continents. Its ITS dataset is strongly Eurocentric, with ~90% of detections originating from Europe, where it is recorded in multiple countries, showing notable hotspots in Estonia, Switzerland, and Germany. Asia contributes ~8% (driven largely by China), and North America is sparsely represented by ~2%. It is most frequently detected in cropland (50%), followed by grassland (20%), forest (17%), anthropogenic habitats (9.4%), woodland (2.3%) and shrubland (0.4%) biomes. Most records are from soil samples including topsoil and rhizosphere soil (93.2%), with minor representation in roots and shoots. Occurrences are associated with MAT ~8.4 °C and MAP ~749 mm/year.

##### Notes.

Although strain CBS 862.68 did not sporulate on any of the culture media tested, molecular data enabled its placement in the genus *Thysanorea* and as conspecific with *T. melanica* (Fig. 2). *Thysanorea melanica* is nested within a well-supported subclade comprising six additional species. It is characterised by unbranched, dark brown conidiophores bearing terminal and intercalary conidiogenous cells, and by pale brown conidia that are 1–3-septate when young and 4–6-septate at maturity, measuring (9–)13–37(–45) × (2.5–)3.5–6.5(–8) μm (Liu et al. 2015).

*Thysanorea melanica* is a cosmopolitan, soil-dwelling saprobe, occasionally associated with plant tissues, that thrives across a wide range of environments with a preference for temperate to cool-temperate climates with moderate rainfall. Its frequent occurrence in croplands, grasslands, and forests highlights its ecological versatility and ability to persist in both natural and anthropogenic ecosystems. The predominance of records from cropland soils further implies that agricultural activities may have facilitated its spread and persistence, potentially through soil transport or crop-associated dispersal. This hypothesis is consistent with the occurrence of CBS 862.68 strain, which was isolated from wheat field soil.

#### ﻿ Thysanorea sinensis

Taxon classificationAnimaliaChaetothyrialesHerpotrichiellaceae

(G.Z. Yang & Z.F. Yu) Réblová & Hern.-Restr. comb. nov.

DDFEA18B-D0BE-5D2E-A304-10A4297E2556

860813

##### Basionym.

*Uncispora sinensis* G.Z. Yang & Z.F. Yu, Mycotaxon 116: 172. 2011.

##### Typus.

CHINA • Yunnan province, Mengla County, Xishuangbanna Tropical Botanical Garden; on submerged leaves of an unidentified dicotyledonous plant; Sep 2010; G. Z. Yang (holotype YMF 1.03683; ex-type culture YMF 1.03683).

#### ﻿ Thysanorea wuzhishanensis

Taxon classificationAnimaliaChaetothyrialesHerpotrichiellaceae

(L.P. Chen & Z.F. Yu) Réblová & Hern.-Restr. comb. nov.

D061A206-093B-568E-AEDD-0DB554E51173

860814

##### Basionym.

*Uncispora wuzhishanensis* L.P. Chen & Z.F. Yu, Sydowia 70: 255. 2018.

##### Typus.

CHINA • Hainan Province, Wuzhishan National Nature Reserve; 754 m a.s.l.; on submerged decaying leaves in a stream; 30 Jun 2011; Z. F. Yu (holotype YMF 1.04080).

#### ﻿ Wongia

Taxon classificationAnimalia

Khemmuk, Geering & R.G. Shivas, IMA Fungus 7: 249. 2016.

9ADDBA3E-B024-5074-8CCF-B9C18F1E97AB

##### Description.

See Khemmuk et al. (2016).

##### Notes.

To date, 11 species have been described in *Wongia*. The asexual morphs produce macronematous conidiophores with holoblastic, polyblastic, sympodially elongating conidiogenous cells that generate septate, subhyaline to dark brown, transversely septate or rarely aseptate conidia (e.g. Bao et al. 2021; Crous et al. 2022; Manawasinghe et al. 2024; Wang et al. 2025).

#### ﻿ Wongia pallidopolaris

Taxon classificationAnimalia

Réblová & Hern.-Restr. sp. nov.

FA202411-F0D0-5522-86B7-1E75F222A506

860720

Fig. 11

##### Etymology.

From Latin *pallidus* (pale), and *polaris* (of or relating to the poles). Referring to the conidial pigmentation, in which the apical and basal cells are distinctly paler than the central, more pigmented cells, creating a noticeable bipolar contrast.

##### Typus.

THE NETHERLANDS • Gelderland Province, Wageningen; isolated from sandy soil under continuous wheat; Jan 1970; J. H. van Emden No. 4118, 30 (holotype CBS H-25781 dried culture, ex-type culture CBS 440.70).

##### Culture characteristics.

On CMD colonies 40–41 mm diam., circular, flat, margin entire to slightly fimbriate, diffuse, lanose, with a subtle concentric zoning, beige to grey-beige at the centre, brown at the margin, reverse dark brown. On MLA colonies 38–40 mm diam., circular, raised, margin entire, lanose, composed of camel brown and pale olivaceous brown concentric zones, reverse dark brown. On OA colonies 49–51 mm diam., circular, raised, margin entire, lanose, composed of beige, camel brown, dark brown and cinnamon concentric zones, brown at the margin, aerial hyphae at the centre and margin bearing numerous colourless exudates, reverse brown. On PCA colonies 44–45 mm diam., circular, convex, margin entire, lanose, whitish-grey at the centre surrounded with a thin smoke-grey zone, pale beige to light fawn towards the margin, reverse dark brown. Sporulation abundant on CMD, MLA, and PCA, absent on OA.

##### Description in culture.

***Colonies*** on MLA effuse. **Sexual morph.** Not observed. **Asexual morph. *Mycelium*** composed of pale brown, septate, sparsely branched hyphae, 1.5–3 µm wide. ***Conidiophores*** 24–70 × 3.5–5(–5.5) µm, macronematous, mononematous, solitary or aggregated, erect, straight to slightly flexuous, apically almost sinuous, cylindrical, unbranched, occasionally proliferating sympodially, brown, dark brown in the lower part, smooth-walled, septate. ***Conidiogenous cells*** 12–30 × (4.5–)5–6 µm, integrated, terminal, mono- or polyblastic, with one to several denticles, extending sympodially, cylindrical, tapering, sometimes slightly swollen at the apex, pale brown, paler at the apex, smooth-walled; conidiogenesis holoblastic-denticulate. ***Conidia*** (20–)22–28(–30) × 5.5–6.5(–5.5) µm (mean ± SD = 25.2 ± 1.7 × 6.1 ± 0.3 μm), solitary, dry, acropleurogenous, ellipsoid to fusiform to fusiform-clavate, tapering towards both ends, truncate at the base 2–2.5 µm wide, with a conspicuous basal scar, mostly straight, occasionally slightly curved, brown to dark brown, end cells paler then the middle ones, apical cell often with a dark brown tip, smooth-walled to finely roughened, thick-walled, the outer wall partly detaches from the conidium, the detached segments appear as apical or side pocket or wings, sometimes the outer wall is detached around the base imitating a minute frill, 3-septate, mucoid sheath absent; conidial secession schizolytic.

##### Habitat and geographical distribution.

The examined strain was isolated from sandy agricultural soil in the Netherlands. According to the GlobalFungi database, *W. pallidopolaris* was detected in 812 environmental samples. It is cosmopolitan, widely distributed in temperate to subtropical regions, with most records from North America, Europe, and Asia. The main hotspots are in the USA (Michigan, New York and North Carolina), China (Provinces Fujian, Guizhou, Hebei, Jiangxi, Jilin and Yunnan), and central Europe (particularly Switzerland and the Netherlands), while additional, less frequent records originate from Australia and Africa. The species is primarily soil-associated (86.2%), with occasional detection in roots, shoots and rare occurrence in air or water. It is predominantly associated with cropland (59.1%) and grassland (28%) ecosystems, followed by occasional occurrence in the anthropogenic habitats (6.4%), forest (4.2%) and woodland, aquatic, shrubland and wetland biomes. In both USA and China, *W. pallidopolaris* is strongly associated with cropland ecosystems, particularly cereals and legumes (*Zea mays*, *Glycine max*, *Oryza sativa*, *Chenopodium quinoa*). However, in China the species exhibits a broader ecological amplitude, occurring not only in croplands but also in forest soils, rhizospheres, and even aerosols, whereas in the USA it appears to be more restricted to cropland and anthropogenic soils. Occurrences are associated with MAT ~11.8 °C and MAP ~926 mm/year.

##### Notes.

*Wongia pallidopolaris* closely resembles *W. aquatica* (Luo et al. 2019) in having 3-septate, brown conidia with paler end cells. However, *W. aquatica* differs in possessing shorter conidia, measuring 17–21 × 5–7 µm. Phylogenetically, both species form a strongly supported sister relationship within a monophyletic clade that is basal to all remaining *Wongia* species (Fig. 3).

**Figure 11.**
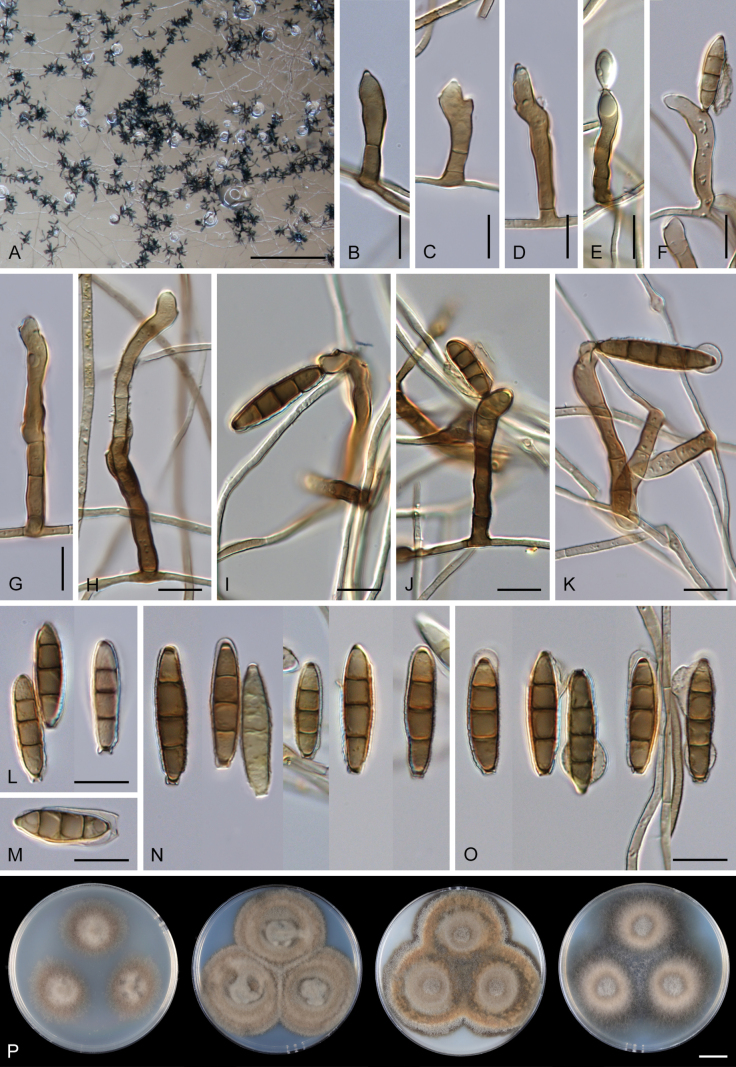
*Wongia pallidopolaris* (ex-type CBS 440.70). **A** Sporulating conidiophores **B–K** conidiophores, conidiogenous cells and conidia **L–O** conidia **P** diversity of colony morphology on CMD, MLA, OA, and PCA, respectively (from left to right) after 4 wk. Images: on MLA (A–O). Scale bars: 300 µm (**A**); 10 µm (**B–O**); 1 cm (**P**).

Most conidia lacked any sheath-like structure; however, in a few cases, a similar feature was observed (Fig. 11O). It appeared on one or both sides near the base or apex, only at the apex, or occasionally at several points on the same conidium. This structure is slightly pigmented and resembles an outer wall layer that detaches in one or several places rather than a mucoid sheath. In other *Wongia* species, a mucoid sheath has not been reported. It is possible that in culture, where osmotic conditions differ from those in nature, the outer conidial wall deteriorates and partially detaches. In contrast, a similar structure observed in *P. parvisporum* represents a true mucoid but ephemeral sheath that is practically invisible on conidia from natural material yet clearly visible in culture, positioned laterally around the middle of the conidium (Fig. 7N–P) or covering the upper two-thirds (Fig. 7D, E).

Based on eDNA data, *W. pallidopolaris* is regarded as a cosmopolitan soil saprobe with strong ecological associations to cropland and grassland ecosystems. Its prevalence in agricultural soils suggests that human activity, particularly through agricultural practices, may have facilitated its dissemination. Its occasional detection in forest soils, rhizospheres, and even aerosols indicates that it can exploit a broader range of habitats. Despite its widespread occurrence in environmental samples, *W. pallidopolaris* represents a morphologically cryptic fungal lineage that has likely been overlooked in traditional surveys.

#### ﻿ Wongia rhachidophora

Taxon classificationAnimalia

Réblová & Hern.-Restr. sp. nov.

3E24ABB5-E9EF-5AAD-96AE-16572B4835CC

860721

Fig. 12

##### Etymology.

From Greek *rhachis* (spine, axis) and *phoros* derived from *phora* (bearing). Referring to the fertile, rachis-like upper part of the conidiophore, which bears conidia along its length.

##### Typus.

INDIA • Bangalore, Arboretum of Forestry Department; on dead leaf of *Bambusa* sp.; Jun 1973; W. Gams (holotype CBS H-11671 dried culture, ex-type culture CBS 531.73, paratype CBS H-5414).

##### Culture characteristics.

On CMD colonies 45–51 mm diam., circular, flat, powdery, sienna, margin paler, cinnamon to fawn, diffuse, fimbriate to slightly lobate, reverse brown with different tones of dark brick, brick to sienna in concentric zones. On MLA colonies 65–67 mm diam., circular, flat, margin entire to fimbriate, velvety to powdery, centre rosy buff, cinnamon to saffron towards the periphery, reverse brown. On OA colonies 58–60 mm diam., circular, flat, margin entire to fimbriate to floccose, velvety, with concentric brown zones of different tones such as fulvous, sienna to cinnamon, ochreous towards the margin, reverse dark brown (umber) in the centre with ochreous margin. On PCA colonies 53–54 mm diam., circular, flat, margin entire fimbriate to diffuse, velvety to sandy, centre fulvous to ochreous, isabelline towards the margin, with numerous exudates at the centre, reverse of the same colour. Sporulation abundant on all media.

##### Description in culture.

***Colonies*** on OA effuse. **Sexual morph.** Not observed. **Asexual morph. *Mycelium*** composed of hyaline to pale brown, smooth hyphae, verrucose close to the conidiophore base, 1–2 μm wide. ***Conidiophores*** up to 63 μm long, 2.5–3.5 μm wide at the base, macronematous, mononematous, solitary, erect, straight to slightly flexuous, cylindrical, unbranched, pale brown to brown-orange. ***Conidiogenous cells*** 13–52 × 3–4 μm, integrated, terminal, sometimes forming transverse septa during sympodial proliferation and becoming intercalary, polyblastic, denticulate, cylindrical to subcylindrical, tapering, pale brown to brown, smooth-walled; conidiogenesis holoblastic-denticulate. ***Conidia*** 8.5–18.5 × 3–5.5 μm (mean ± SD = 14.2 ± 2.4 × 4.0 ± 0.4 μm), solitary, dry, acropleurogenous, oblong-ellipsoidal to ellipsoidal-clavate, rounded at the apex, slightly tapering towards the base, truncate at the base, 0.5­–1 μm wide, smooth-walled, 0–3 septate, hyaline when young, subhyaline to pale brown when mature, pale grey-brown in mass; conidial secession schizolytic.

**Figure 12.**
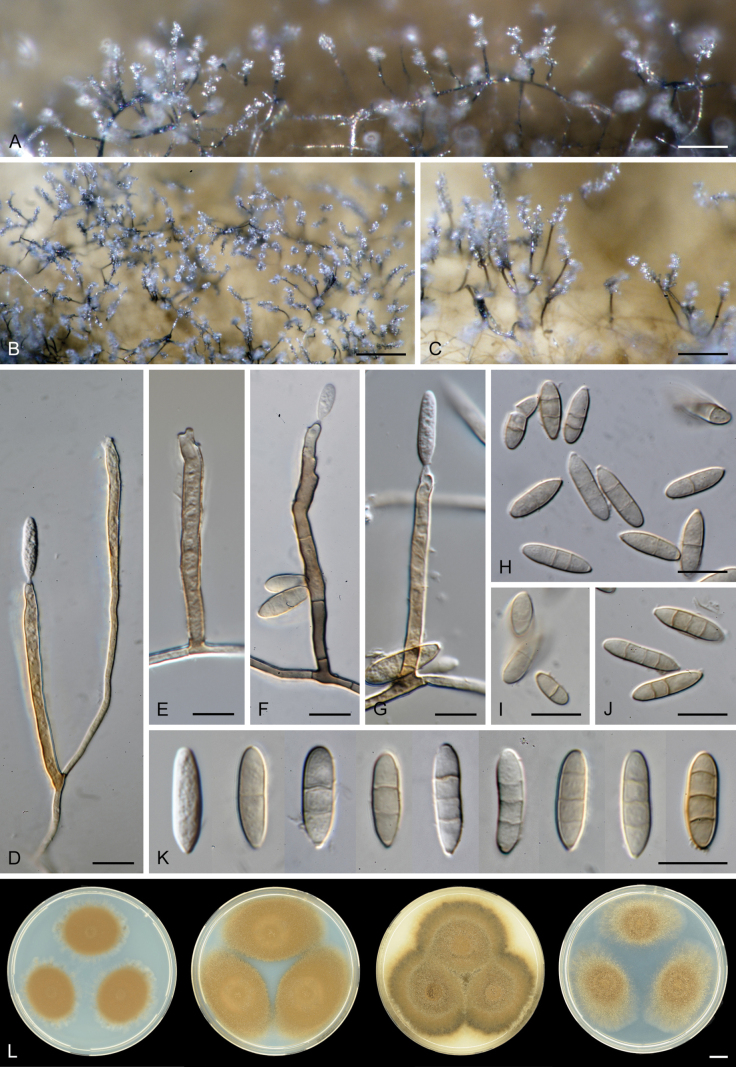
*Wongia rhachidophora* (ex-type CBS 531.73). **A–C** Sporulating conidiophores **D–G** conidiophores, conidiogenous cells and conidia **H–K** conidia **L** diversity of colony morphology on CMD, MLA, OA, and PCA, respectively (from left to right) after 4 wk. Images: on OA (A–K). Scale bars: 50 µm (**A, C**); 100 µm (**B**); 10 µm (**D–K**); 1 cm (**L**).

##### Habitat and geographical distribution.

The species is a saprobe, currently known from two records, including a dead leaf of *Bambusa* sp. in India (this study) and a sample isolated from soil in a pine rock land ecosystem in the coastal savanna biome in Miami, USA (GlobalFungi). Occurrences are associated with MAT ~24.4 °C and MAP ~1441 mm/year.

##### Notes.

In the phylogenetic analyses, *W. rhachidophora* was placed within a species complex comprising also *W. bambusae*, *W. bandungensis*, *W. fusiformis*, and *W. suae* (Fig. 3). Among these, *W. fusiformis* is readily distinguished by its 1–2(–3)-septate, fusiform to clavate, mid-brown conidia, often with paler end cells, measuring 14–18 × 4–5 µm (Bao et al. 2021). *Wongia rhachidophora* is also well differentiated within this complex by its 3-septate, uniformly brown conidia, 8.5–18.5 × 3–5.5 μm. In conidial size and shape it most closely resembles *W. fusiformis*, but the two are resolved as distinct lineages. The remaining three species are particularly difficult to separate morphologically, as they share 1–2(–3)-septate, hyaline to pale brown, oblong to ellipsoidal-clavate conidia. The conidia of *W. suae*, 8–11 × 3–4 µm (Zhang et al. 2023a), and *W. bambusae*, 8–13 × 3–5 µm (Yu et al. 2024), overlap in size, making them almost indistinguishable without molecular data. The conidial length of *W. bandungensis* slightly overlaps with the former two species, but extends to a longer upper range, 11.3–14 × 4.3–5.2 µm (Manawasinghe et al. 2024). A detailed phylogenetic comparison of *W. rhachidophora* with morphologically similar taxa of this species complex is provided in the Results chapter.

*Wongia rhachidophora* appears to be a rare saprobe, with a disjunct distribution between Oceania and subtropical North America. Its presence in a pine rock land biome underscores its adaptability to soil of seasonally dry savannas. However, given the limited records, it is unclear whether this species is truly rare and geographically restricted, or more widespread but overlooked in global fungal diversity surveys.

#### ﻿ Zaanenomyces

Taxon classificationAnimaliaTubeufialesTubeufiaceae

Crous & Osieck, Persoonia 47: 223. 2021.

74A8AE8F-A353-57A2-8545-FD7EE1680A66

##### Description.

See Crous et al. (2021).

##### Notes.

*Zaanenomyces* (Crous et al. 2021) was established for dematiaceous hyphomycetes characterised by erect, macronematous conidiophores and holoblastic, polyblastic, sympodially elongating conidiogenous cells. The fertile region of the conidiogenous cell is transformed into a rachis bearing pimple- or peg-like denticles, each producing hyaline, obclavate, transversely septate conidia. The genus is typified by *Z. quadripartis* and includes three additional species, *Z. hilifer* (this study), *Z. moderatricis-academiae* and *Z. versatilis* (Crous et al. 2021).

#### ﻿ Zaanenomyces hilifer

Taxon classificationAnimaliaTubeufialesTubeufiaceae

Réblová & Hern.-Restr. sp. nov.

6A9BC106-F504-561D-8388-57001FD688D4

860722

Fig. 13

##### Etymology.

From Latin *hilum* (scar, mark) and *fer* (bearing, carrying) derived from *ferre* (to bear, to carry). Referring to the conidial morphology, and the distinct basal hilum.

##### Typus.

IRAN • Golestān Province, Fenderesk District, Alborz Mountains near Shirābād village, Shirābād Waterfall; on *Carex* sp. litter; Jun 2003; W. Gams & R. Zare (holotype CBS H-25777 dried culture, ex-type culture CBS 113561).

##### Culture characteristics.

On CMD colonies 13–14 mm diam., circular, slightly raised, margin entire, cobwebby becoming mucoid, slightly furrowed, salmon to salmon-brown, pink-beige at the margin, reverse pink-beige. On MLA colonies 16–17 mm diam., circular, convex, margin entire, cobwebby, furrowed, salmon-brown with whitish patches, margin salmon, reverse beige with pinkish-orange hue. On OA colonies 14–16 mm diam., circular, flat, cobwebby to velvety, faint orange with diffuse margin, reverse of the same colour. On PCA colonies 12–13 mm diam., circular, flat, margin entire to fimbriate, mucoid and salmon-brown at the centre, cobwebby to velvety and whitish-salmon towards the periphery, reverse of the same colour. Sporulation abundant on OA and PCA, moderate on MLA, absent on CMD.

##### Description in culture.

***Colonies*** on MLA effuse. **Sexual morph.** Not observed. **Asexual morph. *Mycelium*** composed of hyaline to subhyaline, branched, septate, thin-walled hyphae, often swollen, 1.5–3.5 µm wide. Some of the swollen hyphal segments differentiate into thick-walled, subglobose to globose cells, which become basal cells of the conidiophores. ***Conidiophores*** 51.5–98.5 × 3.5–5 µm, basal cells bulbous or lobate, 5–8.5(–10) µm wide, macronematous, mononematous, scattered or loosely aggregated, erect, cylindrical or slightly subulate, with or without nodulose swellings, unbranched, brown to golden brown, basal cell at first subhyaline to pale brown, brown at maturity, smooth-walled, septate. ***Conidiogenous cells*** 6.5–21 × 2.5–3.5 µm, integrated, terminal, sometimes forming transverse septa during sympodial proliferation and becoming intercalary with clusters of closely spaced denticles, monoblastic or polyblastic, denticulate, cylindrical, sometimes with a slight swelling at the apex, pale brown, subhyaline towards the apex, smooth-walled; conidiogenesis holoblastic-denticulate. ***Conidia*** 12–19 × 3–4.5 µm (mean ± SD = 15.2 ± 1.9 × 4.0 ± 0.3 μm), solitary, dry, acropleurogenous, fusiform to narrowly ellipsoidal, subobtuse to acute at the apex, tapering towards the base, often slightly acuminate, truncate at the base ca. 1 µm wide, with a conspicuous basal scar, slightly curved to straight, smooth-walled, with (1–)3(–4) transverse septa, not constricted at the septa, hyaline, also hyaline in mass; conidial secession schizolytic.

##### Habitat and geographical distribution.

The species is a saprobe on litter of *Carex* sp. in Iran. According to the GlobalFungi database, it is widespread in Europe in numerous countries, with hotspots in Switzerland, Czech Republic, and Germany, and secondary occurrences in North America (USA, Canada) and Asia (China). It was detected in 149 samples isolated predominantly from soil (80.5%), with occasional recovery from deadwood (8.7%), roots (6%), air (2.7%) and shoots (2.1%). It is most often found in grasslands (49%), with notable representation in shrubland (16.1%), forest (16.1%) and cropland (11.4%) biomes, and less frequently in anthropogenic habitats, woodlands, wetlands and aquatic systems. The species thrives in temperate climates, MAT ~10.3 °C, MAP ~770 mm/year.

**Figure 13.**
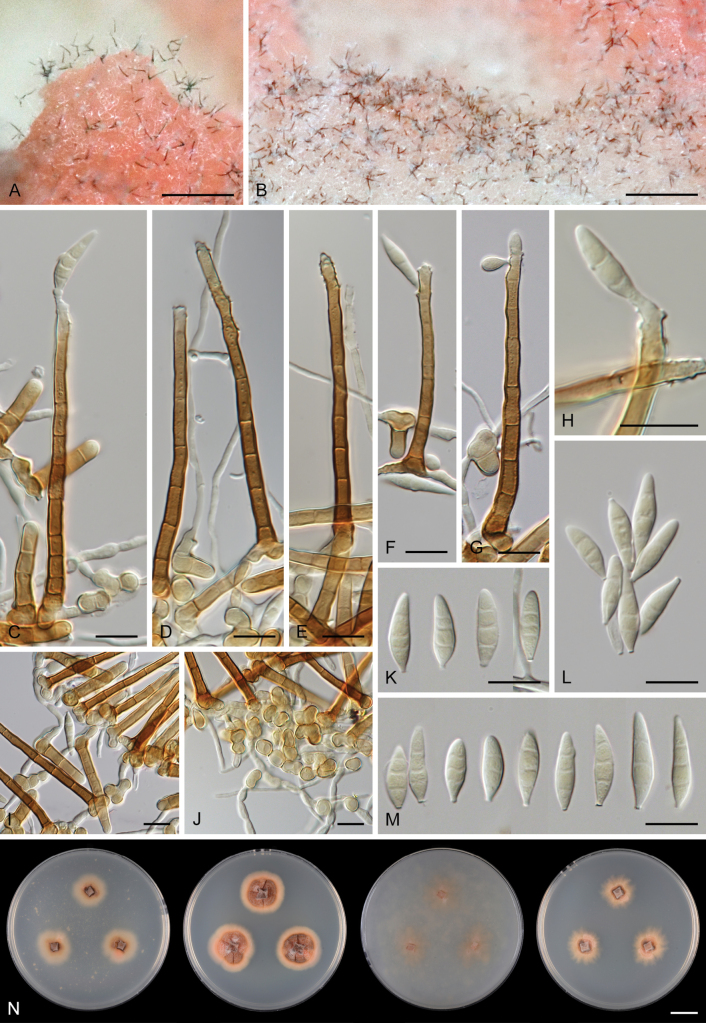
*Zaanenomyces hilifer* (ex-type CBS 113561). **A, B** Sporulating conidiophores **C–G** conidiophores, conidiogenous cells and conidia **H** upper part of the conidiogenous cell with the attached conidium **I** lobate basal cells of conidiophores **J** pigmented, thick-walled cells on vegetative hyphae from which arise conidiophores **K–M** conidia **N** diversity of colony morphology on CMD, MLA, OA, and PCA, respectively (from left to right) after 4 wk. Images: on MLA (A–M). Scale bars: 300 µm (**A, B**); 10 µm (**C–M**); 1 cm (**N**).

##### Notes.

*Zaanenomyces hilifer* is readily distinguished from the other three species described by Crous et al. (2021) by its smaller, fusiform to narrowly ellipsoidal conidia, 12–19 × 3–4.5 μm, which are predominantly 3-septate. The closely related *Z. quadripartis* differs in having longer conidia, (12–)23–30(–35) × (2.5–)3 µm. Both species possess macronematous conidiophores with occasional nodulose swellings along the stipe and a conspicuous terminal rachis. Moreover the basal cell in *Z. hilifer* is bulbous to lobate, while basal cell of *Z. quadripartis* is swollen or with rhizoids (Crous et al. 2021). *Zaanenomyces versatilis* is characterised by straight to geniculately curved, semi-macronematous conidiophores, and longer conidia, (16–)43–50(–55) × (2.5–)3(–3.5) µm, which are subcylindrical, pale brown, (3–)7–10(–12)-septate. In addition, *Z. moderatricis-academiae* also produces longer, (32–)40–52(–57) × 2.5–3 µm, 4–10-septate, pale brown conidia on micronematous conidiophores, often reduced to single, mostly monoblastic conidiogenous cells. These morphological differences, together with molecular evidence, support the recognition of *Z. hilifer* as a distinct species.

*Zaanenomyces hilifer* is a widely distributed, soil-dwelling saprobe with strong representation in temperate biomes, especially grasslands and croplands. Its presence across Europe, North America, and Asia highlights a cosmopolitan distribution pattern, likely facilitated by anthropogenic soil movement and plant associations. Despite being recently described, eDNA sequencing suggests it is neither rare nor geographically restricted, but instead an ecologically versatile species that may be overlooked due to its cryptic morphology.

## ﻿Discussion

The accurate identification of fungi forms the foundation of fungal taxonomy and supports a wide range of scientific outcomes. These include the preservation of strains in public culture collections and dried specimens in fungaria, as well the development of curated databases, biodiversity assessments, red lists, atlases, identification keys, and monographs. Repositories that curate data derived from living strains or herbarium specimens serve as reliable reference points and a veritable minefield for scientists engaged in data-centric work.

A major challenge arises when fungal strains are deposited without verification by existing ex-type or authentic reference material, which would otherwise enable straightforward comparison of their DNA barcodes. In such cases, when comparative material is not available, identifications often depend solely on traditional morphology. While this approach may suffice for species with well-defined, diagnostic traits, it can be misleading when species exhibit simple, convergent, or poorly distinctive morphological traits that recur across distantly related lineages. Morphological interpretation, especially of asexual characteristics, itself is not always straightforward. Subtle features, such as the mode of conidiogenesis, the presence and type of conidiogenous loci, or the development of conidial septa, can be overlooked or misinterpreted, particularly by non-specialists. Such inaccuracies in interpreting morphological features pose the risk of misidentified strains entering public collections. This study illustrates such challenges in the case of *P. parvisporum*.

### ﻿*Myrmecridiaceae*: clarifying the identity of *P. parvisporum*

The genus *Pleurophragmium* was introduced by Costantin (1888) with the main diagnostic feature being “septate spores inserted laterally”, followed by a detailed description and illustration of the single accepted species *P. bicolor* (Fig. 6). However, this species had already been described under two earlier names. Hughes (1958) proposed *Acrothecium simplex* (Berkeley and Broome 1861) and *P. bicolor* are conspecific, and transferred several other species into *Pleurophragmium*, mainly from *Helminthosporium* (Link 1809). Later, Ellis (1968) re-assigned these taxa to *Spiropes* (Ciferri 1955), distinguishing it from *Pleurophragmium* by the presence of a flat double scar at the base of the conidium and on the conidiogenous cell, whereas in *Pleurophragmium* the conidia are borne on tapering denticles. Holubová-Jechová (1972) concluded that the morphologically similar *A. simplex*, *Cordana parvispora* (Preuss 1852), and *P. bicolor* are conspecific, and consequently proposed the new combination *P. parvisporum* for the type species of *Pleurophragmium*.

Von Arx (1970) noted the striking morphological similarity between *Pleurophragmium*, *Scolecobasidium* (Abbott 1927), and *Dactylaria* (Saccardo 1880). Subsequently, due to overlapping conidial morphology, de Hoog and von Arx (1974) transferred *P. parvisporum* to *Dactylaria*, treating *Pleurophragmium* as its generic synonym, and later as one of the four sections proposed within the genus (de Hoog 1985). However, this synonymy was not widely accepted by other authors.

Although *Pleurophragmium* and *Dactylaria*, typified by *D. purpurella*, share septate, hyaline conidia borne on holoblastic-denticulate conidiogenous cells and macronematous conidiophores, they can be distinguished by several characters. *Pleurophragmium* typically develops erect, rigid, pigmented, and often thick-walled conidiophores (particularly on natural substrates). The conidiogenous cells frequently form sympodially extending rachis or nodes with small, pimple-like denticles, and conidia often become lightly pigmented at maturity. *Dactylaria* represents a complex of morphologically similar species that are unlikely phylogenetically homogeneous. It is characterised by short, hyaline to brown conidiophores, cylindrical to tapering denticles, and hyaline, septate conidia that are usually fusiform, naviculate, or cylindrical, although conidial shape may vary considerably within the genus. The heterogeneity of the *Dactylaria* complex has been addressed (de Hoog and Hennebert 1983; de Hoog 1985; de Hoog et al. 1983; de Hoog and von Arx 1985; de Hoog and van Oorschot 1985), and a list of accepted species together with an identification key was provided by Goh and Hyde (1997). Molecular data place *D. purpurella* in close affinity with *Scolecobasidium humicola* (syn. *Ochroconis humicola*, Bussaban et al. 2005) in the *Helotiales*. In contrast, other species historically assigned to *Dactylaria* with available DNA sequences appear unrelated to *D. purpurella* (e.g. Bussaban et al. 2005; Crous et al. 2016; Réblová et al. 2016a; Vu et al. 2019), underscoring the need for further molecular and morphological studies to resolve the taxonomy of this complex.

Based on the molecular and morphological evidence presented in this study, the genus *Pleurophragmium* is reinstated as a separate taxon in the *Myrmecridiaceae* (*Myrmecridiales*, *Sordariomycetes*). It is distantly related to *Dactylaria*, which is accommodated in the *Helotiales (Leotiomycetes)* (Bussaban et al. 2005). *Neomyrmecridium* (Crous et al. 2018a) is treated as a generic synonym of *Pleurophragmium*, and 14 species are accepted in *Pleurophragmium* s. str. Molecular data are still required from additional 19 species treated here as *Pleurophragmium* s. lat.

Since its original description by Costantin (1888), *Pleurophragmium* has evolved into a heterogeneous and broadly circumscribed genus. To date, 44 species and varieties, including synonyms (MycoBank), were historically assigned to *Pleurophragmium*. Over time, 21 *Pleurophragmium* species and varieties have been reassigned to other genera, including *Aquapteridospora* (Bao et al. 2021), *Dactylaria* (Castañeda-Ruiz and Kendrick 1991), *Helminthosporium* (Hughes 1958), *Minimelanolocus* (Castañeda-Ruiz et al. 2001), *Myrmecridium* (Arzanlou et al. 2007), *Pseudospiropes* (de Hoog and von Arx 1974), *Spiropes* (Ellis 1968), *Strossmayeria* (Iturriaga and Korf 1990), *Thysanorea* (Hernández-Restrepo et al. 2020), and *Virgariella* (de Hoog and Hermanides-Nijhof 1977).

The *Myrmecridiales* was established by Crous et al. (2015a) to accommodate a single genus, *Myrmecridium*, originally proposed to include *Ramichloridium schulzeri* and its varieties. Later, Crous et al. (2018a) introduced *Neomyrmecridium* as a distinct lineage within the order, distinguished from *Myrmecridium* primarily by its septate conidia.

Species of *Pleurophragmium* s. str., now including those formerly placed in *Neomyrmecridium*, are morphologically very similar and display a broad yet overlapping range of conidial characteristics. Conidia are usually septate, although aseptate forms may also occur but always within the same species, likely reflecting variation in conidial ontogeny. Pigmentation spans from hyaline to subhyaline, pale brown with age, sometimes with paler end cells, and the surface ranges from smooth-walled to verrucose or irregularly ornamented (crumpled-like) due to the collapsing mucoid sheath. In the protologue of *Neomyrmecridium* (Crous et al. 2018a), conidia were described as having the upper two-thirds encased in a mucoid sheath, but this feature appears to be uncommon and may vary depending on the mounting medium used for observation. It has been reported in *P. asiaticum* and *P. septatum* in culture (Crous et al. 2018a) and occasionally in *P. luguense* (Xu et al. 2023) and *P. sorbicola* (Crous et al. 2018b) on natural substrate. Our observations suggest that this trait varies among species, and even within a species, depending on whether conidia are formed *in vivo* or *in vitro*, and that the age of material also plays a role. For example, we observed mucoid sheath in cultured conidia of *P. parvisporum* (CBS 770.83) but not in conidia from natural substrates, including herbarium material from which the culture was derived. The sheath appears to be ephemeral and is likely to collapse as the conidium matures. In culture, the sheath has a sharp, well-defined margin and shows variation in shape. It originates laterally on the conidium at both sides, as wing-like extensions or pockets. It can expand apically to envelop the upper two-thirds of the conidium and may eventually rupture at the apex. The morphological comparison of *Pleurophragmium* spp., corroborated by molecular data, demonstrates that conidial septation represents interspecific variability rather than a diagnostic character that would support recognition of an independent genus-level lineage.

A comprehensive revision of *Pleurophragmium* lies beyond the scope of this study. Although *Neomyrmecridium* was only recently established and molecular data are available for all its described species, the lack of ex-type cultures, recently recollected material, or molecular data for many other species historically placed in *Pleurophragmium* continues to hinder a robust taxonomic reassessment of the genus. To clarify the taxonomy of *Pleurophragmium*, we conducted a morphological comparison of the accepted species, which provides additional insights into the morphological affinities and distinctions of its species (Table 3). Based on this evaluation, we did not identify any *Neomyrmecridium* species that could be regarded as conspecific with already known *Pleurophragmium* species, although some taxa merit closer examination.

### ﻿*Herpotrichiellaceae*: novel lineages in *Thysanorea*

The holoblastic-denticulate mode of conidiogenesis, with conidia often borne on a sympodially elongating rachis, occurs in several genera of the *Herpotrichiellaceae*, including *Aciculomyces* (Torres-Garcia et al. 2023), *Fonsecaea* (Najafzadeh et al. 2009), *Neoveronaea* (Qiu et al. 2022), *Rhinocladiella* (Melin and Nannfeldt 1934; de Hoog and Hermanides-Nijhof 1977; Müller et al. 1987), *Veronaea* (Ciferri and Montemartini 1957), and *Thysanorea*. In addition, Hernández-Restrepo et al. (2020) reported a phialidic synasexual morph of *Thysanorea seifertii*, which is produced only in culture. *Fonsecaea* and *Rhinocladiella* also display marked pleomorphism, exhibiting considerable variation in asexual morphology, including several types of synasexual morphs, and conidial ontogeny under varying conditions (Schol-Schwarz 1968; McGinnis 1983; de Hoog and Hermanides-Nijhof 1977; Tsuneda et al. 1986). The pleomorphic nature of several genera in this family further complicates identifications based solely on morphology and has historically contributed to their polyphyly within the *Herpotrichiellaceae*.

The genus *Thysanorea*, based on *T. papuana*, was established for fungi with mature conidiophores bearing several tiers of branchlets arranged apically in a compact cluster, imparting a distinctly arboreous appearance (Arzanlou et al. 2007). The sympodially elongating conidiogenous cells are terminal or intercalary on branchlets, occasionally discrete, with a conspicuous denticulate rachis. Recent studies have shown that the traditionally diagnostic branching pattern of *Thysanorea* conidiophores, used to distinguish the genus from *Periconiella* (Saccardo and Berlese 1885), can vary with culture conditions and may be less pronounced in nature or in young cultures (Kirschner 2016; Wang et al. 2019; Hernández-Restrepo et al. 2020).

Hernández-Restrepo et al. (2020) pointed out that *Thysanorea* is closely related to *Minimelanolocus* (Castañeda-Ruiz 2001). However, the placement of the *M. navicularis*, the type species of the genus, remains uncertain due to the absence of DNA sequence data, with its presumed phylogenetic position in the *Herpotrichiellaceae* inferred from other species currently accepted in the genus (e.g. Liu et al. 2015; Wang et al. 2019). Hernández-Restrepo et al. (2020) highlighted morphological differences between *M. navicularis*, and species currently assigned to *Minimelanolocus* based on molecular data. Consequently, these authors transferred sequenced *Minimelanolocus* species to *Thysanorea* and emended the genus.

In addition, molecular data support the transfer of three *Uncispora* species to *Thysanorea*, for which we propose new combinations. Our phylogenetic analyses resolved ex-type strains of *U. hainanensis* (Li et al. 2015), *U. sinensis* (Yang et al. 2011), and *U. wuzhishanensis* (Liu et al. 2018a), within the *Thysanorea* clade. This evidence expands the morphological concept of *Thysanorea* by incorporating new conidial characteristics. These taxa share distinctive apically tapering, cylindrical conidia, curved in the upper portion or terminating in a hooked apical cell, and are morphologically incompatible with *U. harroldiae*, the type species of *Uncispora* (Sinclair and Morgan-Jones 1979). The latter is characterised by holoblastic, determinate conidiogenous cells with a single flat locus, whereas the analysed species possess polyblastic, holoblastic cells that elongate sympodially, and conidia are borne on denticles. The phylogenetic position of *Uncispora* s. str. remains unresolved. These findings emphasise the need for careful identification of *Uncispora* species, particularly considering the diagnostic value of conidiogenous cell morphology and shape of conidia, but also suggest the polyphyly of *Uncispora*, warranting a narrower circumscription of the genus based on freshly recollected material.

### ﻿*Papulosaceae*: expanding the diversity of *Wongia*

Although *Wongia* was originally established for two sexually reproducing species (Khemmuk et al. 2016), several subsequently described taxa have been defined solely by asexual features (Luo et al. 2019; Bao et al. 2021; Manawasinghe et al. 2024; Yu et al. 2024; Wang et al. 2024). Both new species, *W. pallidopolaris* and *W. rhachidophora*, conform to the generic concept of *Wongia* in producing macronematous, unbranched, dark brown conidiophores with polyblastic, sympodially proliferating conidiogenous cells and septate, brown conidia. Their phylogenetic placement within the genus expands the known diversity of *Wongia*.

Currently, the genus *Wongia* comprises 13 species. *Wongia pallidopolaris* and *W. aquatica* formed a strongly supported subclade and include species with relatively short conidiophores (≤ 90 µm long) and dark brown conidia with distinctly paler end cells. By contrast, all other known species, including *W. rhachidophora*, represent morphologically distinct and phylogenetically strongly supported group characterised by subhyaline to pale brown conidia that are mostly evenly pigmented, and only rarely have end cells slightly paler than the median cells. Within the second subclades are grouped the only species known that reproduce also sexually, namely *W. ficherai*, *W. griffinii*, and *W. guttulata* (Khemmuk et al. 2016; Wang et al. 2024). They are characterised by perithecial, non-stromatic ascomata, filiform paraphyses, unitunicate asci with a non-amyloid apical ring, and 3-septate, dark brown ascospores with pale brown to subhyaline end cells.

*Wongia rhachidophora* was nested within a complex comprising four other closely related species. While *W. suae*, *W. bandungensis*, and *W. bambusae* are morphologically indistinguishable, differing only by subtle variation in conidial size, *W. rhachidophora* and *W. fusiformis* are clearly separated from them by conidial colour, septation, and shape. Comparisons of ITS, *tef1*, and *rpb2* sequences further support *W. rhachidophora* as a distinct species. In contrast, *W. bandungensis* and *W. suae* show almost complete sequence identity in *tef1* and *rpb2* and differ by 1.4% in ITS. Given their morphological indistinguishability and minimal genetic divergence, these two taxa may represent very recently diverged populations or are likely conspecific, pending further population-level sampling.

### ﻿*Tubeufiaceae*: new genera and species with holoblastic-denticulate conidiogenesis

*Skoliomycella flava* and *Z. hilifer* are new additions to the family *Tubeufiaceae*. The holoblastic conidiogenesis on denticulate conidiogenous cells is a feature that is widespread and convergent across the family. *Skoliomycella* and *Zaanenomyces* are members of a well-supported subclade comprising fungi characterised by holoblastic (mostly holoblastic-denticulate) conidiogenesis and three conidial morphotypes, including *Acanthostigma* (De Notaris 1863; Réblová and Barr 2000; Boonmee et al. 2014) and *Helicosporium* (Nees von Esenbeck 1816; Linder 1929; Lu et al. 2018a) with helicosporous conidia, *Neodictyospora* (Zhang et al. 2023b) with dictyosporous conidia, and *Camporesiomyces* (Hyde et al. 2020) and *Zaanenomyces* (Crous et al. 2021) with straight, rarely helicosporous conidia. Among these genera, *Skoliomycella* is morphologically most similar to *Camporesiomyces* and *Zaanenomyces*, but differs in having sinuous to geniculate conidiophores bearing scattered denticles along their entire length.

*Zaanenomyces*, typified by *Z. quadripartis*, was established by Crous et al. (2021) for dematiaceous hyphomycetes with simple, erect conidiophores, terminal conidiogenous cells that extend sympodially forming a rachis and dry, solitary, hyaline, narrowly obclavate, septate conidia. All three *Zaanenomyces* species have been recorded from dead culms of *Juncus* spp. (Crous et al. 2021). Among these, *Z. hilifer* is closely related to *Z. quadripartis*. Both species exhibit comparable conidiophore and conidiogenous cell architecture; however, they can be distinguished by differences in conidial morphology. In our preliminary phylogenetic analyses of ITS–LSU sequences (data not shown), *Z. moderatricis-academiae* (ex-type CBS 148312, and CBS149453) and *Z. versatilis* (ex-type CBS 148315), formed either a lineage distinct from *Z. quadripartis–Z. hilifer* subclade, or a monophyletic but statistically unsupported clade in the ML analyses. Therefore, additional loci (*rpb2* and *tef1*) were sequenced for these three species to complete their dataset. In the final four-gene analyses (Fig. 4), all four species were recovered as a monophyletic clade with moderate statistical support in the ML analysis.

Given the variable topology of the *Zaanenomyces* clade observed in several phylogenetic analyses, ex-type strains of three *Zaanenomyces* species were cultivated and examined for cultural characteristics and micromorphology. Comparative analysis showed that *Z. moderatricis-academiae* and *Z. versatilis* differ from *Z. hilifer* and *Z. quadripartis* in several distinct morphological and cultural features. *Zaanenomyces moderatricis-academiae* and *Z. versatilis* have much paler, often hyaline and micronematous conidiophores, particularly pronounced in the former species, and their conidia germinate rapidly. In contrast, *Z. hilifer* and *Z. quadripartis* produce brown, macronematous conidiophores with a conspicuous, pigmented basal cell. Colony morphology also varies; colonies of *Z. moderatricis-academiae* and *Z. versatilis* are uniformly dark from the centre to periphery, whereas *Z. quadripartis* and *Z. hilifer* display a more distinct, paler margin and certain zonation.

The instability in phylogenetic resolution of the *Zaanenomyces* clade across analyses suggests that its current circumscription may not adequately capture the full extent of morphological variability among its species. This warrants further investigation through expanded taxon sampling, the inclusion of additional molecular loci, and consideration of a possible revision of the generic concept. Correspondingly, our morphological observations indicate that *Z. moderatricis-academiae* and *Z. versatilis* form a morphologically cohesive subgroup within *Zaanenomyces*, distinguishable from the lineage comprising *Z. hilifer* and *Z. quadripartis*. This raises the possibility that they may represent a separate, yet closely related taxon.

Morphologically, *Zaanenomyces* is remarkably similar to *Camporesiomyces* (Hyde et al. 2020), making both genera difficult to distinguish. *Camporesiomyces* was originally described for two sexually reproducing species and the asexual *C. vaccinii* (syn. *Helicoma vaccinii*, Carris 1989), which has helicosporous conidia borne on denticulate conidiogenous cells. Han et al. (2025) expanded the genus by adding three species defined solely by asexual characteristics, namely dematiaceous, macronematous conidiophores often in small clusters, holoblastic-denticulate conidiogenous cells, and hyaline to pale brown, septate, straight conidia, thereby broadening the generic concept to include a non-helicosporous conidial shape. The ex-type strain of *H. vaccinii* (ATCC 66068) is also preserved as CBS 216.90, and the latter is the source of the DNA sequences available in GenBank (Tsui et al. 2006). In this study, we examined CBS 216.90 *in vitro*, and we confirm that it matches the protologue and produces the characteristic helicosporous conidia. However, helicoid conidial morphology was omitted from the protologue of *Camporesiomyces* (Hyde et al. 2020) and was not adequately addressed by Han et al. (2025), who suggested that the occurrence of two conidial types within the genus might be the result of geographical isolation in *C. vaccinii*.

*Camporesiomyces vaccinii* is readily distinguished from *Helicoma* by its conidiophores and conidial morphology. In *C. vaccinii*, conidiophores terminate in an integrated, denticulate conidiogenous cell, and the conidia are coiled but asymmetrical with a distinct basal cell that is elongated, tapering, and truncate at the base. In contrast, *Helicoma* develops setiform conidiophores with numerous intercalary conidiogenous cells, usually bearing one or two denticles, and produces symmetrical, coiled conidia with cells that often become progressively smaller from the centre toward both ends. The conidiophore architecture of *C. vaccinii* is consistent with other *Camporesiomyces* species that form straight conidia. Notably, the straight, distinctly truncate basal cell of the otherwise coiled conidium of *C. vaccinii* may reflect an intermediate stage in the morphogenetic diversification of conidial forms within *Camporesiomyces*, pointing to a broader morphological plasticity in the genus than previously recognised.

Conidial morphology within the *Tubeufiaceae* is highly diverse. The helicoid, septate, hyaline to pale brown conidial type is the most common morphotype that occurs in the majority of genera in the family. In addition to the straight, hyaline, septate conidia of *Camporesiomyces*, *Skoliomycella*, and *Zaanenomyces*, other forms include darkly pigmented dictyoconidia of monodictys-like asexual morphs, described in the life cycles of *Chlamydotubeufia* (Boonmee et al. 2011), *Dictyospora* (Brahmanage et al. 2017), *Manoharachariella* (Bagyanarayana et al. 2009), *Muripulchra* (Luo et al. 2017), *Neochlamydotubeufia* (Lu et al. 2018a), *Neodictyospora* (Zhang et al. 2023b), and *Tubeufia amazonensis* (Samuels et al. 1987). Whereas the helicosporous and straight conidia are produced on holoblastic-denticulate conidiogenous cells, the dictyoconidia arise from holoblastic conidiogenous cells with a flat, non-denticulate locus. *Tubeufia amazonensis* also produces a pycnidial, asteromella-like synasexual morph (synanamorph). The systematic placement of *T. amazonensis* remains uncertain. Based on ascomatal wall characters, Crane et al. (1998) transferred this species to *Thaxteriella*, a genus characterised by helicosporous conidia. However, no molecular data are currently available for *Thaxteriella* spp., leaving its phylogenetic position unresolved.

## ﻿Conclusions

This study highlights the importance of routine molecular and morphological verification of strains obtained even from well-curated culture collections. The case of *Pleurophragmium parvisporum* demonstrates how subtle morphological characters, if misinterpreted, can obscure true phylogenetic relationships. Our findings indicate that historical misidentifications persist and may perpetuate taxonomic inaccuracies if left unverified.

Phylogenetic analyses revealed that seven strains deposited under the name *P. parvisporum* represent a polyphyletic assemblage distributed across four families and/or orders in three classes, namely in the *Dothideomycetes (Tubeufiales)*, *Eurotiomycetes (Chaetothyriales)*, and *Sordariomycetes* (*Myrmecridiales* and *Papulosaceae*). Only one strain, CBS 770.83, conforms to the species concept of *P. parvisporum*, confirming its placement within the *Myrmecridiales*. These results led to the synonymisation of *Neomyrmecridium* with *Pleurophragmium* and the proposal of one new genus, several new species, new names and combinations.

## Supplementary Material

XML Treatment for Pleurophragmium

XML Treatment for Pleurophragmium asiaticum

XML Treatment for Pleurophragmium asymmetricum

XML Treatment for Pleurophragmium fluviale

XML Treatment for Pleurophragmium fusiforme

XML Treatment for Pleurophragmium gaoligongense

XML Treatment for Pleurophragmium guizhouense

XML Treatment for Pleurophragmium jiulongheense

XML Treatment for Pleurophragmium luguense

XML Treatment for Pleurophragmium naviculare

XML Treatment for Pleurophragmium parvisporum

XML Treatment for Pleurophragmium pteridophytophilum

XML Treatment for Pleurophragmium septatum

XML Treatment for Pleurophragmium sichuanense

XML Treatment for Pleurophragmium sorbicola

XML Treatment for Pleurophragmium angamosense

XML Treatment for Pleurophragmium aquaticum

XML Treatment for Pleurophragmium bitunicatum

XML Treatment for Pleurophragmium clavatum

XML Treatment for Pleurophragmium ellipsoideum

XML Treatment for Pleurophragmium indicum

XML Treatment for Pleurophragmium harunganae

XML Treatment for Pleurophragmium malaysianum

XML Treatment for Pleurophragmium miniumbonatum

XML Treatment for Pleurophragmium naviculiforme

XML Treatment for Pleurophragmium obcampanuloides

XML Treatment for Pleurophragmium peruamazonicum

XML Treatment for Pleurophragmium peruamazonicum var. inflatum

XML Treatment for Pleurophragmium subfusiforme

XML Treatment for Pleurophragmium taiwanense

XML Treatment for Pleurophragmium tricolor

XML Treatment for Pleurophragmium varieseptatum

XML Treatment for Pleurophragmium verruculosum

XML Treatment for Pleurophragmium yunnanense

XML Treatment for Aquapteridospora bambusinum

XML Treatment for Dactylaria arecae

XML Treatment for Dactylaria cylindrospora

XML Treatment for Dactylaria triseptata

XML Treatment for Helminthosporium flumeanum

XML Treatment for Minimelanolocus leptotrichus

XML Treatment for Minimelanolocus subulifer

XML Treatment for Myrmecridium schulzeri

XML Treatment for Myrmecridium schulzeri var. tritici

XML Treatment for Pseudospiropes costaricensis

XML Treatment for Spiropes capensis

XML Treatment for Spiropes dorycarpus

XML Treatment for Spiropes effusus

XML Treatment for Spiropes guareicola

XML Treatment for Spiropes helleri

XML Treatment for Spiropes palmetto

XML Treatment for Spiropes scopiformis

XML Treatment for Strossmayeria atriseda

XML Treatment for Strossmayeria basitricha

XML Treatment for Thysanorea rousseliana

XML Treatment for Virgariella hippotrichoides

XML Treatment for Skoliomycella

XML Treatment for Skoliomycella flava

XML Treatment for Thysanorea

XML Treatment for Thysanorea acropleurogena

XML Treatment for Thysanorea hainanensis

XML Treatment for Thysanorea melanica

XML Treatment for Thysanorea sinensis

XML Treatment for Thysanorea wuzhishanensis

XML Treatment for Wongia

XML Treatment for Wongia pallidopolaris

XML Treatment for Wongia rhachidophora

XML Treatment for Zaanenomyces

XML Treatment for Zaanenomyces hilifer

## ﻿Additional information

### Use of AI

No use of AI was reported.

### Adherence to national and international regulations

All the fungal strains used in this study have been legally obtained, respecting the Convention on Biological Diversity (Rio Convention).

### Author ORCIDs

Martina Réblová https://orcid.org/0000-0001-5229-1709

Jana Nekvindová https://orcid.org/0000-0002-2861-5483

Lucie Bauchová https://orcid.org/0009-0001-2658-9103

Margarita Hernández-Restrepo https://orcid.org/0000-0001-5157-3392

### Data availability

All of the data that support the findings of this study are available in the main text or Supplementary Information.

## Supplementary materials

Supplementary material 1 Alignments of DNA sequences Data type txt Explanation note Alignments of sequences of members of the *Myrmecridiales*, *Herpotrichiellaceae*, *Papulosaceae* and *Tubeufiaceae*. https://binary.pensoft.net/file/1477357This dataset is made available under the Open Database License (http://opendatacommons.org/licenses/odbl/1.0/). The Open Database License (ODbL) is a license agreement intended to allow users to freely share, modify, and use this Dataset while maintaining this same freedom for others, provided that the original source and author(s) are credited.Martina Réblová

Supplementary material 2 Biogeographical distribution, sample type, habitat and other detailed metadata for newly described species inferred from the GlobalFungi database Data type xlsx https://binary.pensoft.net/file/1477358This dataset is made available under the Open Database License (http://opendatacommons.org/licenses/odbl/1.0/). The Open Database License (ODbL) is a license agreement intended to allow users to freely share, modify, and use this Dataset while maintaining this same freedom for others, provided that the original source and author(s) are credited.Martina Réblová
